# Towards more effective beryllium chelation: an investigation of second-sphere hydrogen bonding†

**DOI:** 10.1039/d0ra08706h

**Published:** 2020-11-04

**Authors:** Tyson N. Dais, David J. Nixon, Penelope J. Brothers, William Henderson, Paul G. Plieger

**Affiliations:** School of Fundamental Sciences, Massey University Private Bag 11 222 Palmerston North 4442 New Zealand p.g.plieger@massey.ac.nz; School of Chemical Sciences, University of Auckland Private Bag 92019 Auckland 1142 New Zealand; Chemistry, School of Science, University of Waikato Private Bag 3105 Hamilton 3240 New Zealand

## Abstract

A comparative study between three experimentally known beryllium chelators (EDTA, NTP, and 10-HBQS) and two tetradentate tripodal di-pyridine-based receptors (HL and HL-NH_2_), specifically designed to bind Be^2+^ cations, has been undertaken in the aqueous phase at the B3LYP/6-311++G(d,p) computational level. The relative binding energies of these five ligand systems to a variety of first row and pre-transition metal cations have been calculated, specifically to investigate their binding strength to Be^2+^ and the binding enhancement that a second sphere hydrogen bonding interaction could afford to the pyridyl based systems. The complexes of EDTA were calculated to have the highest average binding energy; followed by those of NTP, HL-NH_2_, HL, and finally 10-HBQS. The calculated binding energy of the HL-NH_2_Be complex, which includes second sphere interactions, was found to be almost 9% greater than the HL Be complex, with an average binding energy increase of 13.5% observed across all metals upon inclusion of second sphere hydrogen bonding.

## Abstract 2

Secondary-sphere H-bonding is shown to strengthen the binding of tetra-dentate pyridyl-derived ligands towards beryllium.

## Graphical abstract

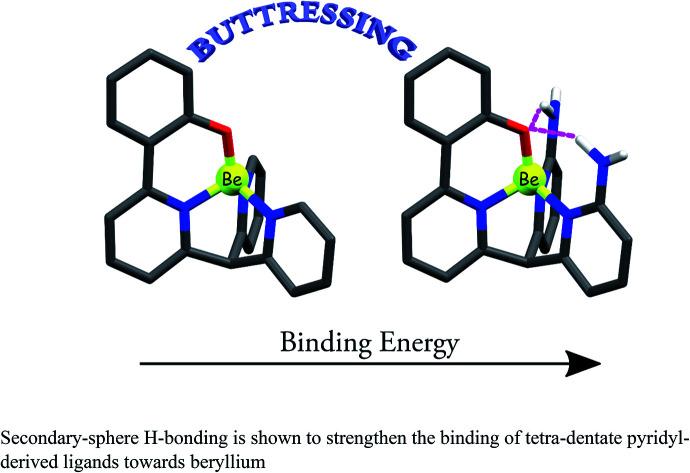

## Introduction

In an increasingly technological age, understanding the chemistry of the elements incorporated into new advances is vital for ensuring the safe use and disposal of products in both industry and consumer settings. Beryllium remains a crucial part of automotive, aviation, nuclear, and consumer industries,^1–7^ due to its unique combination of high rigidity, low density, thermal stability and conductivity.^1–4,8^ However, beryllium is a class A carcinogen^9,10^ and is considered to be the most toxic non-radioactive element, as well as the cause of life-threatening chronic beryllium disease.^1,11,12^

Beryllium is the least electropositive alkali metal and tends to favour a tetrahedral coordination geometry, ligated by medium-to-hard donors such as oxygen and nitrogen.^13–16^ Despite a renewed renaissance in beryllium coordination chemistry,^7,17–21^ an as yet unresolved issue remains, that is, the development of ligands which exhibit both high selectivity and have sufficiently high binding constants to be useful in the detection and remediation of beryllium. The handling of beryllium poses serious potential health and safety risks, making its study, even within a laboratory environment, problematic. Beryllium-ligand interactions can be studied *via* mass spectrometry^22–24^ as it only requires a minute quantity of the compound to be handled at any time, and even then typically in the solution state. Other common alternatives include the use of other less problematic elements as chemically equivalent models, or the use of computational chemistry.^25–29^ Molecular modelling is a powerful tool for studying metal–ligand systems and can provide insights in the prediction of complex geometries, giving further information on the binding sites while also predicting the reactivity and spectroscopic properties of the system. Computational methods have been shown to provide good insight into the coordination chemistry and spectroscopic properties of beryllium and other related small metal cation containing complexes. We have had previous success predicting energetics of beryllium complexes using theoretical modelling,^30,31^ and in particular using DFT methods to obtain reliable geometries for a number of different ligand systems.^25,28,32^

**Fig. 1:**
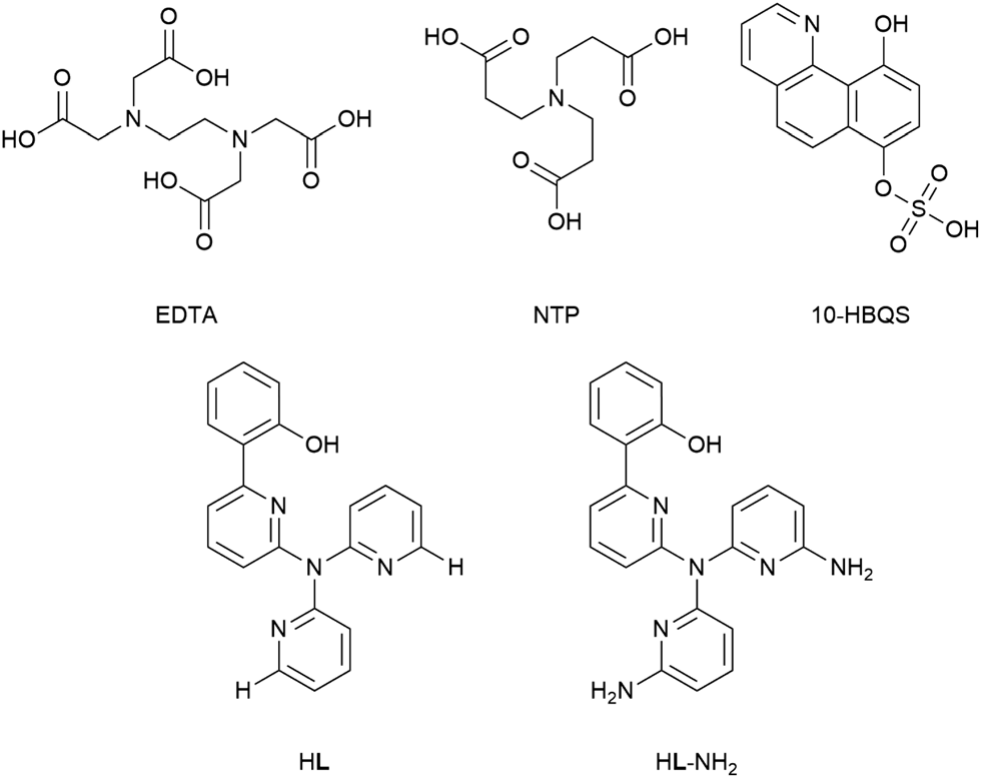
The five ligands examined in this study.

A previous report by Plieger *et al.*^25^ examined a series of pyridyl containing ligands with a variety of appended “buttressing-groups” which provided the functionality necessary for a second coordination sphere. We now report a comparative study on the relative binding enhancement achieved through the inclusion of second sphere bonding interactions. In this study three well-known beryllium chelators, ethylenediaminetetraacetic acid (EDTA), nitrilotripropionic acid (NTP), and 10-hydroxybenzo[*h*]quinoline-7-hydrogen sulfate (10-HBQS) were investigated and compared with two pyridyl containing ligands, HL and HL-NH_2_; the latter of these pyridyl ligands is capable of additional second sphere interactions (Fig. 1). The three experimentally known chelators are known to bind to Be^2+^ and have been used in chemical studies as well as therapeutic and qualitative hazard testing.^33–35^

## Computational details

Literature has shown that, although controversial, when paired with a sufficiently large basis set, B3LYP^36^ can be used to accurately determine the geometry and energetics of small low-nuclearity complexes,^18,25,29,32,35,37,38^ and accurately account for the effects of intra- and intermolecular hydrogen bonds.^39–41^ Benchmarking calculations were performed with three other density functionals: B3LYP-D3,^42^ M06-2X,^43^ and ωB97X-D.^44^ Calculations were performed using the unbuttressed complex (HL Be) and the buttressed complex (HL-NH_2_Be) using the 6-311++G(d,p) basis set. Each functional was found to give the same trend in binding energy, where the magnitude of the trend varied by only a few percent on average. As this work involved a large array of ligands and metals, and was desirable to be further extended, economical calculations were required. Therefore, the B3LYP functional was chosen as it provided an efficient use of computer time as well as giving quantitative results of the expected trends. As such, we have used B3LYP^36^ with the 6-311++G(d,p) basis set and the SCRF-IEFPCM solvation model to carry out all geometry optimisations, frequency, and single point energy calculations. The relative binding energies (*E*_bind_) of the metals to each ligand were obtained as the difference between the energy of the complex and those of the two interacting sub-units in their respective equilibrium conformations, corrected for zero-point energy (ZPE) (eqn (1)).

1 *E*_bind_ = *E*_complex_ − (*E*_ligand_ + *E*_metal_)

## Results and discussion

### Geometric indices

**Fig. 2:**
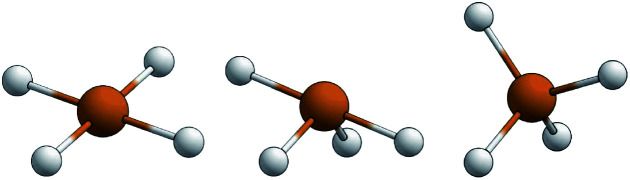
Representations of the idealised square planar geometry (left), seesaw geometry (middle) and tetrahedral geometry (right).

The structural parameters *τ*_4_ and 
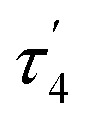
 (eqn (2) and (3), respectively) distinguish the geometry found at the centre of a four-coordinate complex, and take values from 0 to 1.^45,46^ These indices quantify how close the geometry is to an ideal: square planar geometry 
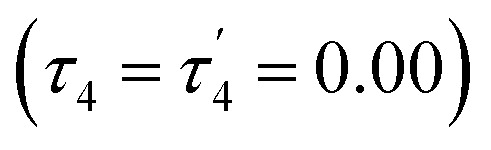
, seesaw geometry (*τ*_4_ ≈ 0.43, 
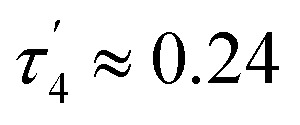
), or tetrahedral geometry 
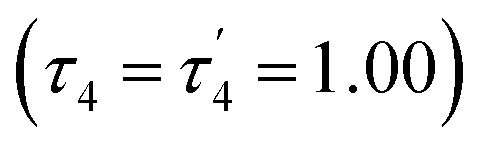
 (Fig. 2).2
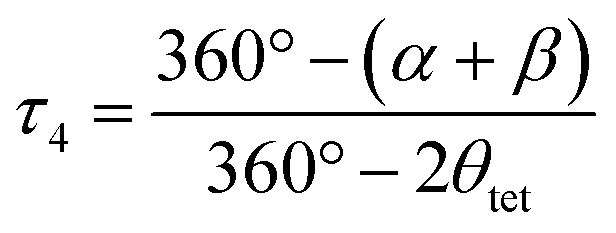
3
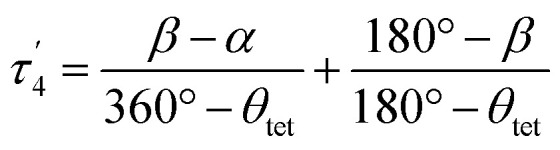


In eqn (2) and (3)*α* and *β* are the two greatest valence angles at the coordination centre, and *θ*_tet_ is the ideal tetrahedral angle (109.5°).

### Ligand structure

**Fig. 3:**
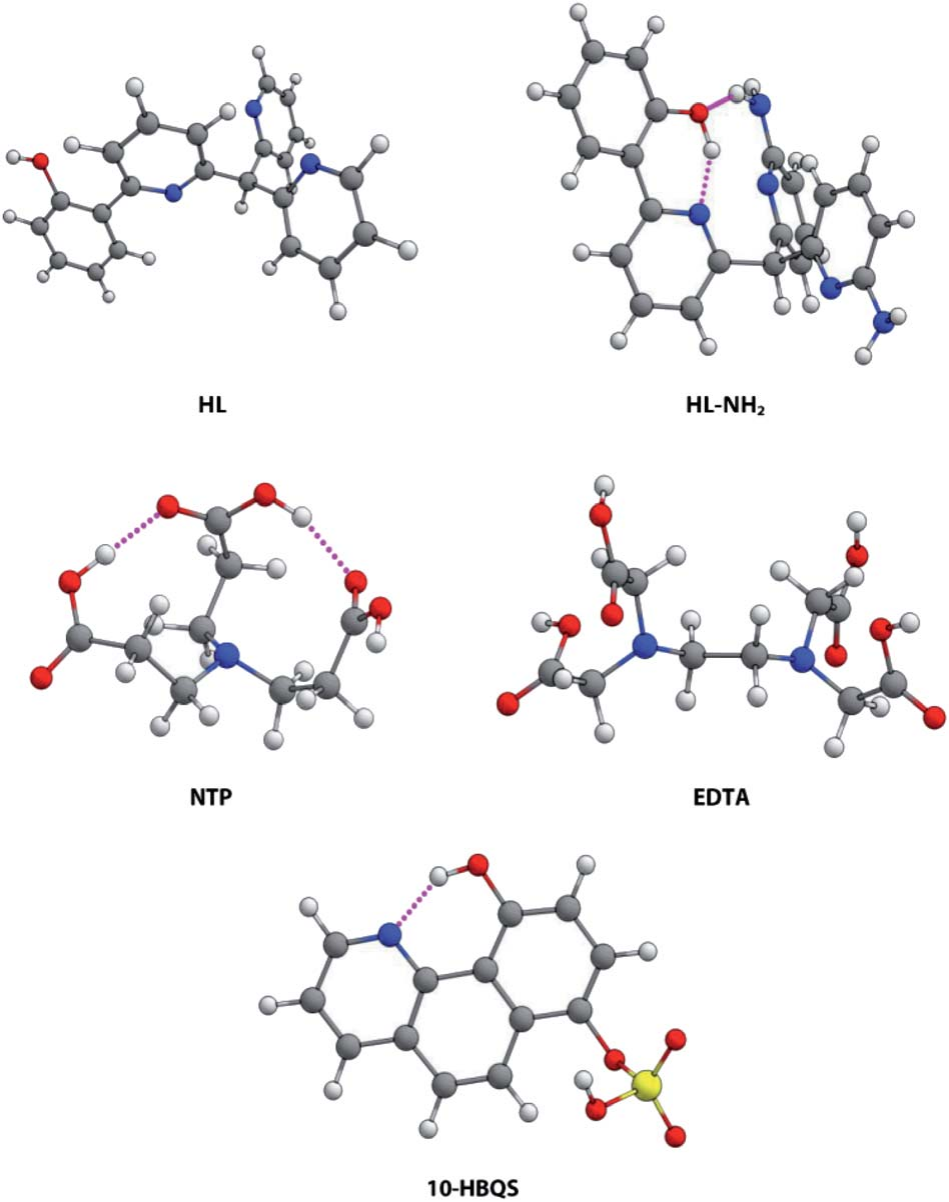
Aqueous phase geometry optimised chelators HL, HL-NH_2_, NTP, EDTA, and 10-HBQS; at B3LYP/6-311++G(d,p).

Of the five ligands investigated, EDTA adopts the most flexible motif, owing to the ethylene bridge connecting its two tertiary nitrogens. This allows for a large bite angle with up to six donor atoms, suitable for the full encapsulation and chelation of a wide range of metal cation sizes. Hydrogen bonding between the carboxylic acids (NH⋯O, 1.758 and 1.859 Å) dictate some pre-organisation in NTP, which forms a partial cavity. Upon deprotonation NTP has seven donor atoms, four of which are able to coordinate due to their position, resulting in the formation of tetrahedral complexes. For the chelator 10-HBQS, a hydrogen bond exists between its aromatic nitrogen and the proton of the nearby phenol (1.791 Å) which are its only two available donor atoms. The sulfate residue is not in a position to aid in chelation, but has been included to mirror the real world experimental ligand (Fig. 3).

### Metal binding study

**Fig. 4:**
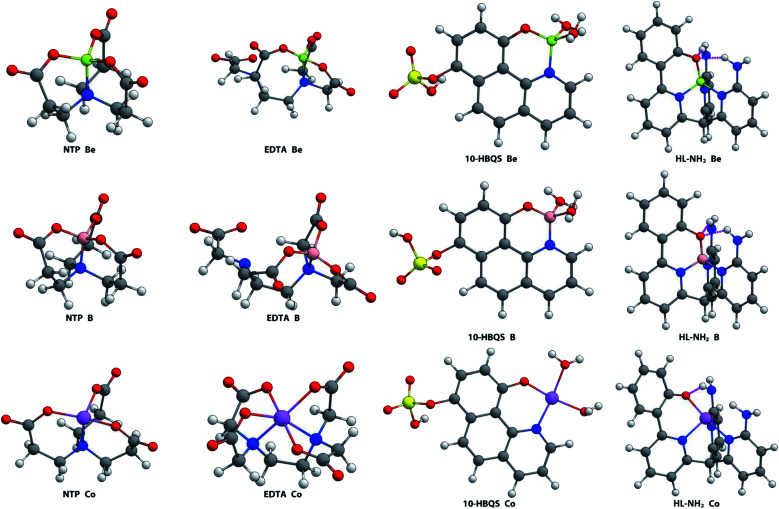
Aqueous phase geometry optimised complexes at B3LYP/6-311++G(d,p).

While a vast range of metals were investigated, B^3+^ and Co^2+^ were found to form the two most comparable complexes to that of Be^2+^ (Fig. 4). The boron and beryllium complexes all formed tetrahedral-type geometries. The ligand 10-HBQS is unique in this set in that it does not offer sufficient donors, but instead requires water molecules to complete the coordination sphere. With a weaker chelation effect, potential for binding strength is not maximised. NTP provides close to perfect tetrahedral arrangements for B^3+^ and Be^2+^, having *τ*_4_ values of 0.98 and 0.97 respectively. This contrasts with EDTA forming significantly more distorted tetrahedra (*τ*_4_ = 0.92 and 0.81, respectively), and to a lesser extent, the less hindered 10-HBQS (*τ*_4_ = 0.93 and 0.91, respectively). In addition, these geometries are more favourable than that of HL (*τ*_4_ = 0.93 and 0.83 respectively) and HL-NH_2_ (*τ*_4_ = 0.92 and 0.84 respectively). These known chelators (NTP, EDTA, and 10-HBQS) all have, on average, significantly shorter bond lengths to stronger donors (carboxylic acids *vs.* pyridyl amines).

The Co^2+^ cation adopted a different coordination geometry upon binding to each ligand. To NTP, it forms a pseudo-tetrahedral arrangement of donors (*τ*_4_ = 0.74), but its bond lengths are all significantly shorter than those of the B^3+^ and Be^2+^ complexes (Table 1), which is a consequence of its larger ionic radius. Cobalt was found to form an octahedral complex with EDTA, again due to its large ionic radius and the presence of 6 readily accessible donor groups, and thus is expected to have a higher binding energy to EDTA than Be^2+^, a result supported by experimental data.^47,48^ A square planar complex was observed to form with Co^2+^ and 10-HBQS, with a *τ*_4_ value of 0.10 being very close to that of the ideal square planar geometry. Finally, to HL-NH_2_, Co^2+^ took a conformation closest to a seesaw geometry (*τ*_4_ = 0.65 and 
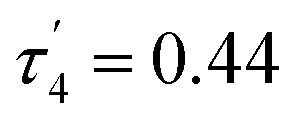
), in this complex only one hydrogen bond forms (O⋯H = 2.04 Å). With the smaller cations, B^3+^ and Be^2+^, a second hydrogen bond forms between the phenolic oxygen and primary amine buttresses of HL-NH_2_.

**Table:** Averaged donor – metal bond lengths (in Å) for the B^3+^, Be^2+^, and Co^2+^ complexes

Bond length (Å)	B^3+^	Be^2+^	Co^2+^
HL_O–M_	1.394	1.530	1.852
HL_N–M_	1.577	1.760	1.998
HL-NH_2__O–M_	1.414	1.549	1.858
HL-NH_2__N–M_	1.587	1.733	2.010
HL-NH_2__H–bond_	2.007	2.116	2.043
NTP_O–M_	1.460	1.612	1.961
NTP_N–M_	1.619	1.791	2.019
EDTA_O–M_	1.504	1.680	2.162
EDTA_N–M_			2.000
10-HBQS_O–M_	1.379	1.531	1.840
10-HBQS_N–M_	1.528	1.697	1.943
10-HBQS_O_water_–M_	1.555	1.696	2.015

Many of the metals in this study were observed to adopt octahedral geometries, however the large Na^+^ and K^+^ cations (with ionic radii of 1.02 and 1.38 Å, respectively) did not fit well into the binding cavity of these chelators and formed unwieldy conformations. The binding energy of each complex was determined and are shown in Table 2. The strongest binding was found in cases where the metal cation has a small ionic radius and high charge, *i.e.* a high charge density. Further, it was found that the binding energy is relatively small when the ionic radius was large or in cases where the tetrahedral arrangement had significant octahedral distortions. EDTA was calculated to be the strongest binding agent across all metals investigated, while 10-HBQS proved to be the weakest chelator having the lowest calculated binding energy for all cations except K^+^, Mn^2+^, V^2+^, and V^3+^. While the presence of the buttressing group in HL-NH_2_ showed enhancement of binding over HL in most cases, NTP still provided stronger binding for the more highly charged metal cations. In the cases where HL-NH_2_ exhibits little enhancement over HL, the geometry was found to be distorted towards a seesaw conformation with only one hydrogen bond present.

Aqueous phase binding energy (kJ mol^−1^) of each complex calculated at B3LYP/6-311++G(d,p)

**Table:** 

Chelator	Al^3+^	B^3+^	Be^2+^	Ca^2+^	Co^2+^	Co^3+^	Cr^2+^	Cr^3+^	Cu^2+^	Fe^2+^
HL	−326.85	−735.30	−357.47	−198.39	−387.81	−537.10	−198.09	−414.46	−365.71	−351.56
HL-NH_2_	−358.03	−755.78	−389.03	−191.94	−397.20	−541.34	−330.19	−425.13	−384.72	−360.88
NTP	−474.56	−806.39	−401.74	−240.20	−371.16	−564.62	−306.04	−482.50	−371.36	−345.44
EDTA	−610.97	−817.59	−415.38	−345.30	−439.00	−830.13	−400.95	−645.56	−462.17	−430.82
10-HBQS	−190.86	−491.27	−249.71	−156.68	−275.97	−347.90	−195.26	−287.82	−276.76	−256.57

**Table:** 

Chelator	Fe^3+^	K^+^	Li^+^	Mg^2+^	Mn^2+^	Na^+^	Ni^2+^	V^2+^	V^3+^	Zn^2+^
HL	−514.31	−93.00	−199.47	−218.63	−130.46	−130.71	−343.50	−179.19	−254.00	−328.94
HL-NH_2_	−521.98	−103.12	−216.07	−224.00	−335.73	−138.40	−357.43	−263.00	−471.27	−344.42
NTP	−559.88	−96.93	−206.10	−270.49	−323.28	−133.83	−319.50	−275.09	−529.98	−559.88
EDTA	−730.81	−140.13	−225.41	−359.68	−397.16	−184.19	−445.58	−359.53	−688.92	−440.23
10-HBQS	−345.26	−95.91	−178.82	−145.80	−252.33	−125.22	−271.38	−179.72	−323.44	−221.00

The average binding energies were calculated for each ligand (Table 3) and compared to the binding energy of the Be^2+^ cation. The complexes of K^+^, Li^+^, and Na^+^ were removed as outliers as their corresponding binding strengths were the lowest (due to a combination of their ionic radii and low charge). The well known chelators NTP, EDTA, and 10-HBQS were calculated to bind a majority of the other investigated metals better than Be^2+^. The chelator HL was determined to have a binding energy to Be^2+^ that was 4.0% above that of the average binding energy to HL, and HL-NH_2_ was found to have a binding energy to Be^2+^ just 0.58% below its average. For Be^2+^ binding to NTP, EDTA, and 10-HBQS, the energies were 5.2%, 20%, or 5.0% below the average binding energy, respectively. Although this qualitative comparison is not comprehensive, it does indicate how these ligands may behave towards a range of metal cations. Although EDTA is the strongest binder for Be^2+^, it binds better on average to all other metals. This reflects what is observed for EDTA experimentally, binding many metals strongly and indeterminately, but less so towards Be^2+^. This lack of selectivity is exploited in many applications, from its use as an additive in AA, to its use as a pre-treatment in remediation protocols utilising 10-HBQS for the detection of beryllium.

**Table:** Comparison of the averaged binding energy (kJ mol^−1^) for the complexes of di- and tricationic metals

	*E*_bind_ (average)	*E*_bind_ (Be^2+^)
HL	−343.62	−357.47
HL-NH_2_	−391.30	−389.03
NTP	−423.65	−401.74
EDTA	−520.93	−415.38
10-HBQS	−262.81	−249.71

The results of these calculations indicate that HL-NH_2_, with its pre-organised binding cavity formed by intramolecular hydrogen bonds, has a secondary sphere of interactions which stabilises certain metal complexes due to the tightening of the binding site (as demonstrated by their bond lengths and *τ*_4_ values for all non-monocationic complexes except Ca^2+^). The Be^2+^ cation is calculated to have the fifth largest increase in binding energy upon inclusion of second sphere hydrogen bonding, surpassed only by the pseudo-octahedral V^2+^, Mn^2+^, Cr^2+^, and V^3+^ complexes.

## Conclusions

Through the use of DFT, a series of complexes for five different ligand systems with a range of metals have been investigated for their relative binding energies in the aqueous phase. Although the inclusion of second sphere hydrogen bonding interactions from primary amine attachments (going from HL to HL-NH_2_) did yield the highest binding energy for Be^2+^, the calculated binding energy of the Be^2+^ complex of HL-NH_2_ was determined to be 8.8% greater than that of HL. It was also found that the Be^2+^ complex of HL had a higher binding energy than the averaged binding energy of the HL complexes, indicating that the ligand system is well suited to chelating small highly charged metal cations such as B^3+^ and Be^2+^. It appears that a combination of charged donors enhanced by second sphere stabilisation may yet be the answer to strong and selective binding of beryllium.

## Conflicts of interest

There are no conflicts to declare.

## Supplementary Material

RA-010-D0RA08706H-s001
