# Supplementary material for: Towards more effective beryllium chelation: an investigation of second-sphere hydrogen bonding
Source: RSC Adv. 2020 Nov 4;10(66):40142–7. doi: 10.1039/d0ra08706h (PMC9057475; doi:10.1039/d0ra08706h)
Supplement: RA-010-D0RA08706H-s001 [file RA-010-D0RA08706H-s001.pdf]

Electronic Supplementary Material (ESI) for RSC Advances.
This journal is © The Royal Society of Chemistry 2020

# Towards more effective beryllium chelation: An investigation of second-sphere hydrogen bonding

Tyson N. Dais, David J. Nixon, Penelope J. Brothers, William Henderson, and Paul G. Plieger*

Supporting Information:

Gibbs free energy ($\Delta G \approx \varepsilon_0 + G_{correction}$) kJ/mol
xyz cartesian coordinates

HL

ΔG = -1087.602947

| | | | |
|---|---|---|---|
| C | -0.67758 | 2.14381 | 1.77255 |
| C | 0.46667 | 1.60046 | 1.18965 |
| H | -0.58633 | 2.88808 | 2.55707 |
| C | 0.29262 | 0.65084 | 0.18177 |
| H | 1.45565 | 1.89843 | 1.51066 |
| C | 1.46317 | -0.04131 | -0.52395 |
| H | 1.14444 | -0.17333 | -1.55970 |
| C | 3.00516 | 1.61321 | -1.66121 |
| C | 4.16328 | 2.38300 | -1.67317 |
| H | 2.31342 | 1.64117 | -2.49496 |
| C | 4.68428 | 1.46998 | 0.47066 |
| C | 5.02676 | 2.31297 | -0.58245 |
| H | 4.38945 | 3.02188 | -2.51924 |
| H | 5.33248 | 1.38517 | 1.33827 |
| H | 5.94239 | 2.89039 | -0.54692 |
| C | 1.72866 | -1.45148 | -0.00023 |
| C | 1.76708 | -3.12994 | 1.72134 |
| C | 2.50854 | -3.55928 | -0.50591 |
| C | 2.29442 | -4.02041 | 0.78980 |
| H | 1.58179 | -3.44284 | 2.74271 |
| H | 2.91445 | -4.22240 | -1.26466 |
| H | 2.53091 | -5.04384 | 1.05405 |
| C | 2.73920 | 0.79483 | -0.55750 |
| C | 1.47927 | -1.82878 | 1.32184 |
| H | 1.06678 | -1.11527 | 2.02342 |
| N | -0.91459 | 0.25546 | -0.23463 |
| N | 3.57122 | 0.72544 | 0.49144 |
| N | 2.23444 | -2.30945 | -0.90021 |
| C | -1.93321 | 1.73363 | 1.34999 |
| H | -2.84017 | 2.12165 | 1.78728 |
| C | -3.33425 | 0.29479 | -0.20885 |
| C | -3.33696 | -0.40613 | -1.43206 |
| C | -4.59659 | 0.51740 | 0.48235 |
| C | -4.49046 | -0.90253 | -2.02244 |
| H | -2.38211 | -0.55231 | -1.92309 |
| C | -5.76356 | -0.02601 | -0.16397 |
| C | -5.71767 | -0.70506 | -1.36543 |
| H | -4.44397 | -1.42697 | -2.97035 |
| H | -6.71153 | 0.12588 | 0.34458 |
| H | -6.63663 | -1.08525 | -1.80402 |
| O | -4.71245 | 1.13829 | 1.59961 |
| C | -2.03699 | 0.76985 | 0.32252 |

HL Al(III)

ΔG = -1329.760349

| | | | |
|---|---|---|---|
| C | 1.78992 | -0.05975 | 1.28709 |
| C | 0.96116 | -0.15389 | 3.56221 |
| C | -0.36165 | -0.11628 | 3.10568 |
| H | 1.16036 | -0.20489 | 4.62547 |
| C | -0.55926 | -0.05735 | 1.74607 |
| H | -1.19734 | -0.13515 | 3.79114 |
| C | -1.94600 | -0.02032 | 1.08831 |
| H | -2.69303 | -0.03705 | 1.87666 |
| C | -3.12588 | 2.19436 | 0.58407 |
| C | -3.24695 | 3.33344 | -0.21142 |
| H | -3.78222 | 2.03044 | 1.42798 |
| C | -1.42491 | 2.55901 | -1.54864 |
| C | -2.38729 | 3.51825 | -1.29001 |
| H | -4.00858 | 4.06969 | 0.01280 |
| H | -0.72891 | 2.65328 | -2.37140 |
| H | -2.45454 | 4.39051 | -1.92511 |
| C | -2.17174 | -1.24988 | 0.20243 |
| C | -3.30188 | -3.26871 | -0.40947 |
| C | -1.46121 | -2.44901 | -1.69312 |
| C | -2.43956 | -3.40605 | -1.49289 |
| H | -4.07582 | -4.00450 | -0.23047 |
| H | -0.76235 | -2.50869 | -2.51654 |
| H | -2.51689 | -4.24041 | -2.17598 |
| C | -2.14755 | 1.26463 | 0.27915 |
| C | -3.16644 | -2.17923 | 0.45047 |
| H | -3.82326 | -2.05440 | 1.30066 |
| N | 0.48904 | -0.03153 | 0.88300 |
| N | -1.31313 | 1.45497 | -0.77908 |
| N | -1.33612 | -1.39208 | -0.86196 |
| C | 2.01997 | -0.12789 | 2.67312 |
| H | 3.02451 | -0.16292 | 3.06196 |
| C | 2.87603 | -0.01770 | 0.28184 |
| C | 4.21584 | -0.00656 | 0.73456 |
| C | 2.67766 | 0.01489 | -1.13056 |
| C | 5.29508 | 0.02636 | -0.12944 |
| H | 4.42309 | -0.01975 | 1.79436 |
| C | 3.77646 | 0.04523 | -1.99519 |
| C | 5.07299 | 0.05012 | -1.50857 |
| H | 6.30213 | 0.03437 | 0.26797 |
| H | 3.57494 | 0.06615 | -3.05903 |
| H | 5.90741 | 0.07465 | -2.19954 |
| O | 1.45820 | 0.01836 | -1.73643 |
| Al | -0.04325 | 0.02463 | -0.92287 |

HL Be(II)

ΔG = -1102.257071

| | | | |
|---|---|---|---|
| C | -1.69975 | -0.00109 | -1.30112 |

| | | | |
|---|---|---|---|
| C | -0.89206 | -0.00257 | -3.58252 |
| C | 0.43005 | -0.00194 | -3.12342 |
| H | -1.09274 | -0.00338 | -4.64725 |
| C | 0.62366 | -0.00100 | -1.75672 |
| H | 1.26869 | -0.00222 | -3.80640 |
| C | 1.99153 | -0.00026 | -1.07470 |
| H | 2.77794 | -0.00046 | -1.82496 |
| C | 3.10217 | 2.20432 | -0.35380 |
| C | 3.11957 | 3.29075 | 0.51921 |
| H | 3.83759 | 2.10713 | -1.14159 |
| C | 1.20026 | 2.38197 | 1.61939 |
| C | 2.15651 | 3.38320 | 1.52026 |
| H | 3.87858 | 4.05670 | 0.41620 |
| H | 0.42446 | 2.40007 | 2.37462 |
| H | 2.14152 | 4.21353 | 2.21344 |
| C | 2.11754 | -1.24016 | -0.19212 |
| C | 3.12121 | -3.28905 | 0.52260 |
| C | 1.20141 | -2.38010 | 1.62183 |
| C | 2.15816 | -3.38097 | 1.52372 |
| H | 3.88058 | -4.05473 | 0.42038 |
| H | 0.42560 | -2.39783 | 2.37704 |
| H | 2.14355 | -4.21061 | 2.21772 |
| C | 2.11690 | 1.24064 | -0.19343 |
| C | 3.10330 | -2.20351 | -0.35149 |
| H | 3.83868 | -2.10675 | -1.13938 |
| N | -0.41013 | -0.00065 | -0.89389 |
| N | 1.18766 | 1.33610 | 0.77961 |
| N | 1.18832 | -1.33508 | 0.78099 |
| C | -1.94814 | -0.00216 | -2.68794 |
| H | -2.95877 | -0.00271 | -3.06636 |
| C | -2.75235 | -0.00042 | -0.26994 |
| C | -4.11138 | -0.00028 | -0.65212 |
| C | -2.44617 | 0.00014 | 1.12863 |
| C | -5.14151 | 0.00026 | 0.27184 |
| H | -4.37111 | -0.00052 | -1.70248 |
| C | -3.51303 | 0.00060 | 2.05181 |
| C | -4.83302 | 0.00065 | 1.63834 |
| H | -6.17160 | 0.00037 | -0.06282 |
| H | -3.25796 | 0.00095 | 3.10537 |
| H | -5.62743 | 0.00104 | 2.37670 |
| O | -1.21199 | 0.00021 | 1.62137 |
| Be | 0.04205 | 0.00021 | 0.74551 |

HL B(III)

ΔG = -1112.127021

| | | | |
|---|---|---|---|
| C | -1.60695 | -0.00005 | -1.36588 |
| C | -0.78660 | -0.00013 | -3.63892 |
| C | 0.53834 | -0.00010 | -3.18065 |
| H | -0.98497 | -0.00017 | -4.70336 |
| C | 0.73975 | -0.00006 | -1.82285 |
| H | 1.37678 | -0.00012 | -3.86280 |
| C | 2.08153 | -0.00002 | -1.09883 |
| H | 2.90932 | -0.00003 | -1.80068 |
| C | 3.04125 | 2.24303 | -0.20611 |
| C | 2.89695 | 3.28404 | 0.71363 |
| H | 3.85907 | 2.22354 | -0.91322 |
| C | 0.92592 | 2.23485 | 1.57809 |
| C | 1.83328 | 3.28006 | 1.61233 |
| H | 3.61514 | 4.09429 | 0.72502 |
| H | 0.07363 | 2.16182 | 2.23906 |
| H | 1.70163 | 4.07475 | 2.33321 |
| C | 2.10934 | -1.22543 | -0.19326 |
| C | 2.89702 | -3.28396 | 0.71382 |
| C | 0.92596 | -2.23476 | 1.57822 |
| C | 1.83334 | -3.27995 | 1.61252 |
| H | 3.61522 | -4.09419 | 0.72526 |
| H | 0.07367 | -2.16171 | 2.23918 |
| H | 1.70171 | -4.07460 | 2.33344 |
| C | 2.10931 | 1.22545 | -0.19333 |
| C | 3.04129 | -2.24300 | -0.20597 |
| H | 3.85912 | -2.22352 | -0.91309 |
| N | -0.31011 | -0.00003 | -0.96014 |
| N | 1.07901 | 1.23945 | 0.68681 |
| N | 1.07904 | -1.23941 | 0.68688 |
| C | -1.84500 | -0.00011 | -2.74803 |
| H | -2.85781 | -0.00013 | -3.12008 |
| C | -2.63759 | -0.00002 | -0.32970 |
| C | -4.01030 | -0.00002 | -0.64743 |
| C | -2.27566 | 0.00001 | 1.03348 |
| C | -4.97511 | 0.00001 | 0.34537 |
| H | -4.32620 | -0.00003 | -1.68188 |
| C | -3.24423 | 0.00004 | 2.03371 |
| C | -4.58911 | 0.00004 | 1.68991 |
| H | -6.02403 | 0.00001 | 0.07770 |
| H | -2.92373 | 0.00006 | 3.06793 |
| H | -5.33964 | 0.00006 | 2.47131 |
| O | -0.97005 | 0.00002 | 1.43826 |
| B | 0.09591 | 0.00001 | 0.54027 |

HL Ca(II)

ΔG = -1765.133379

| | | | |
|---|---|---|---|
| C | -1.85751 | -0.20375 | 1.23694 |
| C | -0.95836 | -0.51822 | 3.45991 |
| C | 0.31193 | -0.29057 | 2.94263 |

| | | | |
|---|---|---|---|
| H | -1.09482 | -0.72798 | 4.51449 |
| C | 0.44590 | -0.05987 | 1.57499 |
| H | 1.18425 | -0.30091 | 3.58319 |
| C | 1.86409 | 0.17022 | 1.03736 |
| H | 2.48803 | 0.27415 | 1.92309 |
| C | 3.54890 | -1.69820 | 0.79082 |
| C | 4.04272 | -2.81127 | 0.11885 |
| H | 4.03225 | -1.33813 | 1.69021 |
| C | 2.27509 | -2.54120 | -1.46003 |
| C | 3.39240 | -3.24584 | -1.03204 |
| H | 4.91905 | -3.32958 | 0.48959 |
| H | 1.74087 | -2.84829 | -2.35284 |
| H | 3.73694 | -4.10776 | -1.58850 |
| C | 2.03734 | 1.47738 | 0.26849 |
| C | 3.01540 | 3.66998 | 0.10002 |
| C | 1.57183 | 2.78474 | -1.57986 |
| C | 2.36299 | 3.82891 | -1.11866 |
| H | 3.64323 | 4.45757 | 0.49959 |
| H | 1.04405 | 2.86821 | -2.52399 |
| H | 2.46059 | 4.73587 | -1.70112 |
| C | 2.41827 | -1.04482 | 0.29557 |
| C | 2.85064 | 2.48026 | 0.80117 |
| H | 3.34737 | 2.32761 | 1.75099 |
| N | -0.60596 | -0.03229 | 0.73539 |
| N | 1.79399 | -1.46387 | -0.81662 |
| N | 1.40871 | 1.63349 | -0.90769 |
| C | -2.04483 | -0.47623 | 2.60442 |
| H | -3.04097 | -0.66702 | 2.97870 |
| C | -3.05622 | -0.08370 | 0.37104 |
| C | -4.20062 | 0.53616 | 0.90959 |
| C | -3.11636 | -0.62109 | -0.95664 |
| C | -5.39456 | 0.63473 | 0.20779 |
| H | -4.14031 | 0.97242 | 1.90085 |
| C | -4.36125 | -0.53358 | -1.63775 |
| C | -5.46873 | 0.08017 | -1.07644 |
| H | -6.24885 | 1.13546 | 0.64772 |
| H | -4.41153 | -0.96130 | -2.63377 |
| H | -6.39492 | 0.13593 | -1.63997 |
| O | -2.08865 | -1.18489 | -1.53628 |
| Ca | -0.14038 | -0.16569 | -1.77373 |

HL Co(II)

$\Delta G$ = -2470.174092

| | | | |
|---|---|---|---|
| C | -1.78640 | -0.35727 | -1.28319 |
| C | -0.97690 | -0.57497 | -3.55388 |
| C | 0.33273 | -0.49533 | -3.08127 |
| H | -1.16940 | -0.67508 | -4.61529 |
| C | 0.53052 | -0.35108 | -1.71759 |
| H | 1.17832 | -0.52362 | -3.75544 |
| C | 1.93319 | -0.17050 | -1.14126 |
| H | 2.64210 | -0.25523 | -1.96204 |
| C | 2.99588 | 2.14654 | -1.02866 |
| C | 3.09245 | 3.39569 | -0.42132 |
| H | 3.61159 | 1.89329 | -1.88226 |
| C | 1.39524 | 2.70969 | 1.11674 |
| C | 2.27881 | 3.68409 | 0.67027 |
| H | 3.79263 | 4.13177 | -0.79750 |
| H | 0.73599 | 2.88606 | 1.95792 |
| H | 2.32253 | 4.64274 | 1.17035 |
| C | 2.25675 | -1.24260 | -0.11811 |
| C | 3.54714 | -3.07304 | 0.73647 |
| C | 1.67036 | -2.18963 | 1.92523 |
| C | 2.71042 | -3.10341 | 1.84939 |
| H | 4.36776 | -3.77420 | 0.64617 |
| H | 0.98833 | -2.17038 | 2.76719 |
| H | 2.85462 | -3.82021 | 2.64671 |
| C | 2.08983 | 1.21998 | -0.52139 |
| C | 3.31866 | -2.12980 | -0.26087 |
| H | 3.95174 | -2.07862 | -1.13711 |
| N | -0.50188 | -0.31968 | -0.84830 |
| N | 1.30730 | 1.50384 | 0.53580 |
| N | 1.45126 | -1.28041 | 0.95824 |
| C | -2.03084 | -0.48534 | -2.66261 |
| H | -3.04687 | -0.48331 | -3.02915 |
| C | -2.89180 | -0.21349 | -0.31889 |
| C | -4.16954 | -0.71114 | -0.64836 |
| C | -2.73237 | 0.49018 | 0.91552 |
| C | -5.27437 | -0.50614 | 0.16126 |
| H | -4.29211 | -1.28775 | -1.55717 |
| C | -3.88025 | 0.71529 | 1.71004 |
| C | -5.12350 | 0.23075 | 1.34338 |
| H | -6.23845 | -0.91313 | -0.11812 |
| H | -3.74831 | 1.27278 | 2.63037 |
| H | -5.97924 | 0.41207 | 1.98490 |
| O | -1.57430 | 0.96570 | 1.34927 |
| Co | -0.03295 | -0.00889 | 1.02632 |

HL Co(III)

$\Delta G$ = -2469.956572

| | | | |
|---|---|---|---|
| C | -1.72793 | 1.08831 | -0.82966 |
| C | -0.88611 | 2.39378 | -2.70139 |
| C | 0.38038 | 1.84640 | -2.50280 |
| H | -1.06273 | 3.09250 | -3.50919 |
| C | 0.57061 | 1.00065 | -1.43055 |
| H | 1.20662 | 2.07394 | -3.16207 |

| | | | |
|---|---|---|---|
| C | 1.92603 | 0.35482 | -1.18966 |
| H | 2.57232 | 0.58896 | -2.03076 |
| C | 3.75515 | 1.54959 | 0.16434 |
| C | 4.19928 | 1.98249 | 1.41187 |
| H | 4.33554 | 1.72230 | -0.73210 |
| C | 2.21422 | 1.07173 | 2.39198 |
| C | 3.42043 | 1.74057 | 2.54008 |
| H | 5.14433 | 2.50375 | 1.50012 |
| H | 1.56691 | 0.85989 | 3.23393 |
| H | 3.73335 | 2.06344 | 3.52365 |
| C | 1.76694 | -1.15100 | -1.08014 |
| C | 2.19804 | -3.42381 | -1.69392 |
| C | 0.72010 | -2.88052 | 0.10874 |
| C | 1.34420 | -3.83812 | -0.67607 |
| H | 2.69333 | -4.15252 | -2.32324 |
| H | 0.04105 | -3.14285 | 0.90882 |
| H | 1.15172 | -4.88532 | -0.48690 |
| C | 2.54054 | 0.88502 | 0.08982 |
| C | 2.41208 | -2.06334 | -1.90198 |
| H | 3.06886 | -1.70995 | -2.68561 |
| N | -0.44136 | 0.69412 | -0.57303 |
| N | 1.79709 | 0.65768 | 1.18230 |
| N | 0.94458 | -1.57183 | -0.10331 |
| C | -1.92393 | 2.00227 | -1.88546 |
| H | -2.91202 | 2.38850 | -2.07437 |
| C | -2.88272 | 0.53372 | -0.10902 |
| C | -4.20112 | 0.82188 | -0.52409 |
| C | -2.73574 | -0.37483 | 0.97338 |
| C | -5.30274 | 0.25343 | 0.09122 |
| H | -4.38163 | 1.48880 | -1.35384 |
| C | -3.85908 | -0.96545 | 1.58104 |
| C | -5.13204 | -0.64792 | 1.15005 |
| H | -6.29647 | 0.50246 | -0.25909 |
| H | -3.68900 | -1.65348 | 2.39945 |
| H | -5.99331 | -1.09791 | 1.62921 |
| O | -1.55020 | -0.65543 | 1.48876 |
| Co | 0.08670 | -0.25692 | 0.96679 |

HL Cr(II)

ΔG = -2131.841939

| | | | |
|---|---|---|---|
| C | -2.61311 | 0.82762 | -0.82218 |
| C | -2.31369 | 2.10345 | -2.84701 |
| C | -0.91571 | 1.96995 | -2.78055 |
| H | -2.75251 | 2.61992 | -3.69416 |
| C | -0.41397 | 1.28592 | -1.67224 |
| H | -0.28191 | 2.34259 | -3.57438 |
| C | 3.61325 | -0.02101 | 0.12526 |

| | | | |
|---|---|---|---|
| H | 4.69418 | 0.02933 | 0.09928 |
| C | 3.79686 | 2.20578 | 1.08652 |
| C | 3.23426 | 3.34023 | 1.63424 |
| H | 4.87140 | 2.09125 | 1.03038 |
| C | 1.08069 | 2.39557 | 1.19199 |
| C | 1.84149 | 3.43435 | 1.70379 |
| H | 3.86228 | 4.13981 | 2.00673 |
| H | -0.00089 | 2.45119 | 1.20217 |
| H | 1.34865 | 4.29819 | 2.12867 |
| C | 3.06812 | -1.28138 | -0.23717 |
| C | 3.48714 | -3.59136 | -0.85238 |
| C | 1.26950 | -2.74766 | -0.52179 |
| C | 2.10845 | -3.81179 | -0.82426 |
| H | 4.17213 | -4.39493 | -1.09278 |
| H | 0.19481 | -2.87509 | -0.48741 |
| H | 1.68367 | -4.78450 | -1.03269 |
| C | 2.97761 | 1.15437 | 0.60500 |
| C | 3.96468 | -2.32931 | -0.55807 |
| H | 5.02714 | -2.12356 | -0.55659 |
| N | -1.27423 | 0.88659 | -0.76802 |
| N | 1.61360 | 1.28645 | 0.64497 |
| N | 1.71679 | -1.51019 | -0.24474 |
| C | -3.17155 | 1.51586 | -1.90907 |
| H | -4.24262 | 1.54566 | -2.05938 |
| C | -3.22438 | -0.03671 | 0.18166 |
| C | -4.59535 | 0.04373 | 0.48240 |
| C | -2.41422 | -1.03222 | 0.82981 |
| C | -5.19007 | -0.81077 | 1.39616 |
| H | -5.19392 | 0.80374 | -0.00750 |
| C | -3.05260 | -1.89365 | 1.74763 |
| C | -4.40536 | -1.78442 | 2.02846 |
| H | -6.24636 | -0.72394 | 1.61964 |
| H | -2.44537 | -2.64939 | 2.23244 |
| H | -4.85664 | -2.46132 | 2.74578 |
| O | -1.11200 | -1.22913 | 0.60446 |
| Cr | 0.24485 | -0.03063 | -0.11461 |

HL Cr(III)

ΔG = -2131.748214

| | | | |
|---|---|---|---|
| C | -1.81195 | 0.75885 | -1.05313 |
| C | -0.95621 | 1.81468 | -3.06277 |
| C | 0.32234 | 1.36893 | -2.73834 |
| H | -1.12510 | 2.39131 | -3.96360 |
| C | 0.50294 | 0.68999 | -1.54882 |
| H | 1.16783 | 1.56186 | -3.38431 |
| C | 1.90623 | 0.23119 | -1.16067 |
| H | 2.55049 | 0.40794 | -2.01776 |

| | | | |
|---|---|---|---|
| C | 3.55931 | 1.85265 | -0.07299 |
| C | 3.96072 | 2.56049 | 1.05714 |
| H | 4.10872 | 1.92405 | -1.00231 |
| C | 2.10919 | 1.62498 | 2.24349 |
| C | 3.22620 | 2.44469 | 2.23376 |
| H | 4.83745 | 3.19496 | 1.01804 |
| H | 1.50242 | 1.50399 | 3.13271 |
| H | 3.50576 | 2.97803 | 3.13214 |
| C | 1.95580 | -1.25276 | -0.83443 |
| C | 2.74131 | -3.48956 | -1.17763 |
| C | 1.21237 | -2.95745 | 0.58057 |
| C | 1.97130 | -3.90072 | -0.09342 |
| H | 3.34283 | -4.20365 | -1.72625 |
| H | 0.59042 | -3.22189 | 1.42691 |
| H | 1.95157 | -4.93279 | 0.22894 |
| C | 2.43239 | 1.04626 | 0.00853 |
| C | 2.73397 | -2.14902 | -1.55421 |
| H | 3.32263 | -1.80049 | -2.39235 |
| N | -0.53002 | 0.42784 | -0.70335 |
| N | 1.72632 | 0.94041 | 1.14772 |
| N | 1.21184 | -1.66190 | 0.21086 |
| C | -2.01042 | 1.50501 | -2.22856 |
| H | -3.00049 | 1.84988 | -2.48050 |
| C | -2.97805 | 0.32935 | -0.25623 |
| C | -4.26776 | 0.35723 | -0.82931 |
| C | -2.87665 | -0.15538 | 1.07012 |
| C | -5.39317 | -0.03659 | -0.12424 |
| H | -4.39666 | 0.66319 | -1.85757 |
| C | -4.01866 | -0.54207 | 1.78572 |
| C | -5.26993 | -0.48012 | 1.19616 |
| H | -6.36288 | -0.00813 | -0.60530 |
| H | -3.88736 | -0.89594 | 2.80066 |
| H | -6.14603 | -0.78795 | 1.75461 |
| O | -1.69538 | -0.26814 | 1.68852 |
| Cr | 0.01820 | -0.25615 | 1.13320 |

HL Cu(II)

ΔG = -2727.87861

| | | | |
|---|---|---|---|
| C | -1.81960 | 0.07834 | -1.34054 |
| C | -0.98099 | 0.42758 | -3.58194 |
| C | 0.32469 | 0.30534 | -3.10593 |
| H | -1.16015 | 0.62046 | -4.63288 |
| C | 0.50641 | 0.07836 | -1.75037 |
| H | 1.17664 | 0.40446 | -3.76523 |
| C | 1.90869 | 0.01993 | -1.14005 |
| H | 2.61604 | 0.05808 | -1.96560 |
| C | 3.14016 | 2.18570 | -0.60788 |

| | | | |
|---|---|---|---|
| C | 3.34807 | 3.28071 | 0.22667 |
| H | 3.71118 | 2.06514 | -1.51966 |
| C | 1.65228 | 2.41874 | 1.67343 |
| C | 2.59212 | 3.40187 | 1.38876 |
| H | 4.09021 | 4.02732 | -0.02929 |
| H | 1.03784 | 2.46963 | 2.56445 |
| H | 2.72303 | 4.23733 | 2.06401 |
| C | 2.16540 | -1.27415 | -0.38266 |
| C | 3.38196 | -3.30982 | -0.01373 |
| C | 1.57374 | -2.65193 | 1.40281 |
| C | 2.56780 | -3.56518 | 1.08626 |
| H | 4.16653 | -4.00340 | -0.29060 |
| H | 0.91136 | -2.80578 | 2.24553 |
| H | 2.69278 | -4.45379 | 1.69041 |
| C | 2.17997 | 1.24168 | -0.25182 |
| C | 3.17907 | -2.15119 | -0.75681 |
| H | 3.79751 | -1.92521 | -1.61553 |
| N | -0.54226 | -0.05300 | -0.91757 |
| N | 1.45377 | 1.36301 | 0.87198 |
| N | 1.38150 | -1.53352 | 0.68138 |
| C | -2.04805 | 0.33247 | -2.70559 |
| H | -3.05515 | 0.47764 | -3.06764 |
| C | -2.92869 | -0.02084 | -0.37046 |
| C | -4.21386 | -0.36814 | -0.83765 |
| C | -2.77173 | 0.26485 | 1.02194 |
| C | -5.32170 | -0.40606 | -0.00792 |
| H | -4.33964 | -0.63480 | -1.87961 |
| C | -3.92003 | 0.24701 | 1.84456 |
| C | -5.16843 | -0.07964 | 1.34459 |
| H | -6.28937 | -0.68867 | -0.40374 |
| H | -3.78520 | 0.48851 | 2.89249 |
| H | -6.02443 | -0.09399 | 2.01051 |
| O | -1.61710 | 0.57897 | 1.60233 |
| Cu | -0.05059 | -0.20841 | 1.02135 |

HL Fe(II)

ΔG = -2351.116474

| | | | |
|---|---|---|---|
| C | -1.81913 | -0.40315 | -1.24833 |
| C | -1.00886 | -0.66204 | -3.51500 |
| C | 0.30060 | -0.56484 | -3.04308 |
| H | -1.20032 | -0.78125 | -4.57465 |
| C | 0.49936 | -0.39354 | -1.68369 |
| H | 1.14550 | -0.59749 | -3.71798 |
| C | 1.90998 | -0.18984 | -1.13229 |
| H | 2.59874 | -0.29128 | -1.96830 |
| C | 2.99899 | 2.11255 | -1.11497 |
| C | 3.14188 | 3.37411 | -0.54387 |

| | | | |
|---|---|---|---|
| H | 3.57951 | 1.82640 | -1.98280 |
| C | 1.48964 | 2.75967 | 1.06973 |
| C | 2.37473 | 3.70565 | 0.56914 |
| H | 3.84186 | 4.08638 | -0.96383 |
| H | 0.86620 | 2.97128 | 1.93022 |
| H | 2.45550 | 4.67499 | 1.04337 |
| C | 2.27124 | -1.23956 | -0.09996 |
| C | 3.61623 | -3.04167 | 0.73578 |
| C | 1.77060 | -2.16026 | 1.97396 |
| C | 2.81777 | -3.06275 | 1.87705 |
| H | 4.44221 | -3.73397 | 0.62814 |
| H | 1.12232 | -2.14089 | 2.84220 |
| H | 2.99731 | -3.76397 | 2.68118 |
| C | 2.09424 | 1.21605 | -0.55211 |
| C | 3.33994 | -2.11650 | -0.26529 |
| H | 3.94206 | -2.06926 | -1.16344 |
| N | -0.53279 | -0.35765 | -0.80959 |
| N | 1.35590 | 1.54087 | 0.52449 |
| N | 1.50066 | -1.26694 | 1.00225 |
| C | -2.06202 | -0.55889 | -2.62636 |
| H | -3.07716 | -0.56348 | -2.99480 |
| C | -2.93136 | -0.23282 | -0.29783 |
| C | -4.21079 | -0.72415 | -0.63660 |
| C | -2.78811 | 0.50006 | 0.92280 |
| C | -5.32760 | -0.48408 | 0.14490 |
| H | -4.32446 | -1.32460 | -1.53079 |
| C | -3.95016 | 0.76390 | 1.68555 |
| C | -5.19166 | 0.28534 | 1.30867 |
| H | -6.29082 | -0.88769 | -0.14235 |
| H | -3.82842 | 1.34613 | 2.59184 |
| H | -6.05798 | 0.49637 | 1.92643 |
| O | -1.63445 | 0.96853 | 1.37208 |
| Fe | -0.05445 | 0.01118 | 1.08576 |

HL Fe(III)

ΔG = -2350.913228

| | | | |
|---|---|---|---|
| C | 1.77896 | 0.57874 | 1.16603 |
| C | 0.95255 | 1.34881 | 3.31741 |
| C | -0.33828 | 1.01937 | 2.92006 |
| H | 1.14010 | 1.76660 | 4.29837 |
| C | -0.53336 | 0.53146 | 1.64125 |
| H | -1.18393 | 1.15051 | 3.58101 |
| C | -1.94245 | 0.19373 | 1.18256 |
| H | -2.61576 | 0.33368 | 2.02368 |
| C | -3.45079 | 1.97448 | 0.12054 |
| C | -3.73419 | 2.77872 | -0.98125 |
| H | -4.06378 | 2.00656 | 1.01132 |
| C | -1.85189 | 1.82958 | -2.10847 |
| C | -2.92516 | 2.70608 | -2.11199 |
| H | -4.58076 | 3.45358 | -0.95604 |
| H | -1.18685 | 1.72687 | -2.95778 |
| H | -3.11587 | 3.31396 | -2.98582 |
| C | -2.02374 | -1.25016 | 0.72451 |
| C | -2.89238 | -3.47996 | 0.78637 |
| C | -1.21861 | -2.83408 | -0.79436 |
| C | -2.05050 | -3.81122 | -0.27115 |
| H | -3.55519 | -4.22224 | 1.21335 |
| H | -0.54331 | -3.03860 | -1.61514 |
| H | -2.03179 | -4.80735 | -0.69164 |
| C | -2.35881 | 1.12163 | 0.05848 |
| C | -2.88035 | -2.18233 | 1.29183 |
| H | -3.52546 | -1.89400 | 2.11092 |
| N | 0.48960 | 0.34295 | 0.76743 |
| N | -1.58197 | 1.06233 | -1.03537 |
| N | -1.21276 | -1.58304 | -0.29586 |
| C | 1.99554 | 1.12508 | 2.44456 |
| H | 2.99674 | 1.37803 | 2.75264 |
| C | 2.93949 | 0.25584 | 0.31568 |
| C | 4.25075 | 0.29327 | 0.83243 |
| C | 2.80394 | -0.13614 | -1.03820 |
| C | 5.35533 | -0.00791 | 0.05120 |
| H | 4.42456 | 0.53985 | 1.86910 |
| C | 3.92485 | -0.43345 | -1.83210 |
| C | 5.19511 | -0.36590 | -1.29249 |
| H | 6.34375 | 0.02897 | 0.49176 |
| H | 3.75625 | -0.71973 | -2.86253 |
| H | 6.05805 | -0.59954 | -1.90421 |
| O | 1.61208 | -0.24585 | -1.61458 |
| Fe | -0.04052 | -0.18230 | -1.04051 |

HL K(I)

ΔG = -1687.507056

| | | | |
|---|---|---|---|
| C | -2.02651 | -0.83465 | -0.38030 |
| C | -1.20473 | -2.98018 | -1.11781 |
| C | 0.08032 | -2.45583 | -1.09647 |
| H | -1.37564 | -4.00841 | -1.41708 |
| C | 0.25404 | -1.12721 | -0.69224 |
| H | 0.93386 | -3.05565 | -1.38996 |
| C | 1.67608 | -0.54991 | -0.72342 |
| H | 2.11943 | -0.92851 | -1.64796 |
| C | 1.85196 | 1.55839 | -2.10125 |
| C | 1.90352 | 2.94420 | -2.20625 |
| H | 1.91072 | 0.93566 | -2.98602 |
| C | 1.71056 | 3.04053 | 0.17014 |

| | | | |
|---|---|---|---|
| C | 1.83360 | 3.70787 | -1.04432 |
| H | 2.00240 | 3.41770 | -3.17629 |
| H | 1.65291 | 3.60027 | 1.09931 |
| H | 1.87522 | 4.78958 | -1.07299 |
| C | 2.55697 | -1.08570 | 0.40423 |
| C | 4.68244 | -1.92345 | 1.16303 |
| C | 2.79947 | -1.69263 | 2.61142 |
| C | 4.12865 | -2.06217 | 2.43311 |
| H | 5.71338 | -2.19992 | 0.97426 |
| H | 2.33114 | -1.78489 | 3.58711 |
| H | 4.70580 | -2.44733 | 3.26444 |
| C | 1.72501 | 0.97129 | -0.83788 |
| C | 3.88583 | -1.43073 | 0.13507 |
| H | 4.28332 | -1.31783 | -0.86664 |
| N | -0.76559 | -0.34365 | -0.32843 |
| N | 1.65518 | 1.70641 | 0.28031 |
| N | 2.02538 | -1.21402 | 1.62761 |
| C | -2.27085 | -2.16133 | -0.76154 |
| H | -3.28599 | -2.53767 | -0.77547 |
| C | -3.16916 | 0.07478 | -0.09623 |
| C | -4.15841 | 0.21419 | -1.07913 |
| C | -3.29177 | 0.77147 | 1.16184 |
| C | -5.26475 | 1.04696 | -0.91405 |
| H | -4.04265 | -0.33186 | -2.01156 |
| C | -4.43853 | 1.61970 | 1.29042 |
| C | -5.38900 | 1.75336 | 0.28910 |
| H | -6.00294 | 1.14753 | -1.70134 |
| H | -4.55089 | 2.15977 | 2.22647 |
| H | -6.23966 | 2.41148 | 0.44460 |
| O | -2.45013 | 0.62783 | 2.12853 |
| K | 0.05629 | 0.70749 | 2.46027 |

HL Li(I)

ΔG = -1095.118333

| | | | |
|---|---|---|---|
| C | -1.81177 | -0.44621 | -1.09669 |
| C | -0.93878 | -1.13848 | -3.24663 |
| C | 0.34204 | -0.78946 | -2.81847 |
| H | -1.09150 | -1.54170 | -4.24153 |
| C | 0.48338 | -0.31690 | -1.51711 |
| H | 1.20197 | -0.89971 | -3.46710 |
| C | 1.87630 | 0.01144 | -0.96930 |
| H | 2.56177 | -0.01300 | -1.81592 |
| C | 2.76531 | 2.38526 | -0.97223 |
| C | 2.83458 | 3.65441 | -0.40545 |
| H | 3.32386 | 2.14958 | -1.86976 |
| C | 1.32989 | 2.89266 | 1.28465 |
| C | 2.10266 | 3.91762 | 0.74886 |
| H | 3.45051 | 4.42285 | -0.85791 |
| H | 0.74204 | 3.05468 | 2.18244 |
| H | 2.12643 | 4.88941 | 1.22555 |
| C | 2.34919 | -1.06092 | 0.01221 |
| C | 3.91764 | -2.75919 | 0.66913 |
| C | 1.99962 | -2.21004 | 1.98158 |
| C | 3.14579 | -2.97840 | 1.80673 |
| H | 4.81862 | -3.33497 | 0.49206 |
| H | 1.36842 | -2.34791 | 2.85353 |
| H | 3.41995 | -3.72420 | 2.54205 |
| C | 1.96445 | 1.41300 | -0.36789 |
| C | 3.51456 | -1.78757 | -0.24188 |
| H | 4.09252 | -1.59425 | -1.13719 |
| N | -0.55590 | -0.15534 | -0.68947 |
| N | 1.25888 | 1.66882 | 0.74475 |
| N | 1.60596 | -1.27296 | 1.10876 |
| C | -2.01396 | -0.97938 | -2.38868 |
| H | -3.00238 | -1.27890 | -2.70787 |
| C | -2.95119 | -0.18907 | -0.18853 |
| C | -4.21798 | 0.05207 | -0.76117 |
| C | -2.82430 | -0.17868 | 1.25457 |
| C | -5.36114 | 0.27350 | -0.00749 |
| H | -4.30266 | 0.09496 | -1.84130 |
| C | -4.03291 | 0.02658 | 1.99427 |
| C | -5.25640 | 0.24795 | 1.39162 |
| H | -6.30968 | 0.47116 | -0.49287 |
| H | -3.94704 | 0.01439 | 3.07648 |
| H | -6.13700 | 0.41152 | 2.00596 |
| O | -1.71344 | -0.35735 | 1.89154 |
| Li | -0.02423 | 0.05332 | 1.26671 |

HL Mg(II)

ΔG = -1287.592403

| | | | |
|---|---|---|---|
| C | -1.82719 | 0.34534 | -1.16132 |
| C | -0.96648 | 0.86788 | -3.36624 |
| C | 0.32238 | 0.58327 | -2.91708 |
| H | -1.13162 | 1.18016 | -4.39078 |
| C | 0.48276 | 0.23664 | -1.58405 |
| H | 1.17681 | 0.64977 | -3.57750 |
| C | 1.88989 | -0.01972 | -1.03202 |
| H | 2.56241 | -0.00795 | -1.88745 |
| C | 3.45349 | 1.88710 | -0.42017 |
| C | 3.82993 | 2.91036 | 0.44460 |
| H | 4.01115 | 1.69390 | -1.32740 |
| C | 1.99413 | 2.31127 | 1.84929 |
| C | 3.08705 | 3.12991 | 1.60088 |
| H | 4.69131 | 3.52666 | 0.21662 |

| | | | |
|---|---|---|---|
| H | 1.38628 | 2.44151 | 2.73671 |
| H | 3.34465 | 3.91566 | 2.29863 |
| C | 2.03572 | -1.39321 | -0.37993 |
| C | 3.00726 | -3.58846 | -0.30022 |
| C | 1.42894 | -2.84422 | 1.32821 |
| C | 2.26268 | -3.84379 | 0.84728 |
| H | 3.66934 | -4.34302 | -0.70762 |
| H | 0.82755 | -2.99574 | 2.21678 |
| H | 2.32167 | -4.79371 | 1.36193 |
| C | 2.34324 | 1.10782 | -0.10493 |
| C | 2.89313 | -2.34831 | -0.92154 |
| H | 3.46116 | -2.12235 | -1.81472 |
| N | -0.55605 | 0.13123 | -0.73317 |
| N | 1.62980 | 1.32154 | 1.01660 |
| N | 1.31721 | -1.64602 | 0.73068 |
| C | -2.03346 | 0.75959 | -2.49386 |
| H | -3.02640 | 1.00977 | -2.83617 |
| C | -2.98115 | 0.13663 | -0.25510 |
| C | -4.23905 | -0.13182 | -0.84221 |
| C | -2.90704 | 0.20239 | 1.17858 |
| C | -5.39935 | -0.28546 | -0.10239 |
| H | -4.30297 | -0.25032 | -1.91654 |
| C | -4.11898 | 0.07338 | 1.90736 |
| C | -5.33346 | -0.16386 | 1.29198 |
| H | -6.33773 | -0.50248 | -0.59835 |
| H | -4.05042 | 0.15415 | 2.98679 |
| H | -6.23092 | -0.26874 | 1.89294 |
| O | -1.79915 | 0.39135 | 1.85553 |
| Mg | -0.00521 | -0.05514 | 1.33337 |

HL Mn(II)

ΔG = -2238.333752

| | | | |
|---|---|---|---|
| C | -2.48074 | -0.96433 | -0.77353 |
| C | -2.67945 | -2.53532 | -2.62389 |
| C | -1.28935 | -2.56583 | -2.79056 |
| H | -3.31065 | -3.13699 | -3.26877 |
| C | -0.60473 | -1.77523 | -1.89583 |
| H | -0.80131 | -3.16524 | -3.54571 |
| C | 2.20913 | -0.26771 | -1.01107 |
| H | 2.41322 | -0.79659 | -1.93663 |
| C | 2.68906 | 1.99531 | -2.11231 |
| C | 2.43362 | 3.36193 | -2.10441 |
| H | 3.35865 | 1.54852 | -2.83599 |
| C | 0.94382 | 3.04321 | -0.25444 |
| C | 1.55927 | 3.89982 | -1.15688 |
| H | 2.91273 | 4.00744 | -2.83103 |
| H | 0.23030 | 3.40111 | 0.47814 |
| H | 1.34755 | 4.96056 | -1.12736 |
| C | 2.85820 | -0.83756 | 0.19258 |
| C | 4.50767 | -2.01693 | 1.45909 |
| C | 2.51976 | -1.15576 | 2.48554 |
| C | 3.73419 | -1.81579 | 2.60558 |
| H | 5.45074 | -2.54625 | 1.52659 |
| H | 1.86218 | -1.00368 | 3.33274 |
| H | 4.06078 | -2.17581 | 3.57226 |
| C | 2.06951 | 1.20160 | -1.14413 |
| C | 4.06613 | -1.53684 | 0.23142 |
| H | 4.63986 | -1.68418 | -0.67451 |
| N | -1.12196 | -1.02957 | -0.96729 |
| N | 1.19752 | 1.72753 | -0.25468 |
| N | 2.10377 | -0.67887 | 1.30378 |
| C | -3.26193 | -1.75758 | -1.62830 |
| H | -4.33416 | -1.78503 | -1.49997 |
| C | -3.04271 | -0.10898 | 0.27690 |
| C | -4.39766 | 0.27944 | 0.19891 |
| C | -2.27401 | 0.33615 | 1.39526 |
| C | -5.00154 | 1.04710 | 1.17932 |
| H | -4.98275 | -0.01142 | -0.66429 |
| C | -2.91453 | 1.10473 | 2.39145 |
| C | -4.24998 | 1.45240 | 2.29002 |
| H | -6.04107 | 1.33470 | 1.08113 |
| H | -2.31822 | 1.41964 | 3.23985 |
| H | -4.70927 | 2.05034 | 3.06959 |
| O | -0.99093 | 0.04740 | 1.56583 |
| Mn | 0.34125 | -0.20932 | 0.22882 |

HL Na(I)

ΔG = -1249.873668

| | | | |
|---|---|---|---|
| C | -1.84766 | -0.73102 | -1.02458 |
| C | -0.90390 | -1.77723 | -2.98748 |
| C | 0.34481 | -1.29969 | -2.60178 |
| H | -1.01256 | -2.36405 | -3.89285 |
| C | 0.43948 | -0.56726 | -1.41614 |
| H | 1.22568 | -1.49365 | -3.20136 |
| C | 1.82433 | -0.05766 | -0.99094 |
| H | 2.44719 | -0.14242 | -1.88138 |
| C | 2.41708 | 2.33995 | -1.51152 |
| C | 2.44530 | 3.69023 | -1.17837 |
| H | 2.82995 | 1.99647 | -2.45231 |
| C | 1.36455 | 3.11637 | 0.86977 |
| C | 1.90870 | 4.09224 | 0.04136 |
| H | 2.88103 | 4.41323 | -1.85823 |
| H | 0.93450 | 3.38732 | 1.82929 |
| H | 1.90967 | 5.13098 | 0.34708 |

| | | | |
|---|---|---|---|
| C | 2.48685 | -0.95676 | 0.05455 |
| C | 4.33325 | -2.34946 | 0.72496 |
| C | 2.42885 | -1.98572 | 2.11586 |
| C | 3.66656 | -2.59061 | 1.92271 |
| H | 5.29989 | -2.79846 | 0.52794 |
| H | 1.87584 | -2.14667 | 3.03628 |
| H | 4.08886 | -3.22912 | 2.68844 |
| C | 1.84987 | 1.42448 | -0.61801 |
| C | 3.73621 | -1.52276 | -0.22129 |
| H | 4.22909 | -1.31940 | -1.16423 |
| N | -0.62322 | -0.29640 | -0.64844 |
| N | 1.33119 | 1.81439 | 0.55510 |
| N | 1.84736 | -1.18828 | 1.20976 |
| C | -2.00651 | -1.49552 | -2.19518 |
| H | -2.98592 | -1.86830 | -2.46539 |
| C | -3.04002 | -0.36529 | -0.21990 |
| C | -4.19128 | 0.05493 | -0.90810 |
| C | -3.06603 | -0.47075 | 1.22269 |
| C | -5.37397 | 0.39598 | -0.25925 |
| H | -4.14574 | 0.13816 | -1.98999 |
| C | -4.30653 | -0.12294 | 1.84961 |
| C | -5.41772 | 0.29806 | 1.13861 |
| H | -6.23478 | 0.73558 | -0.82346 |
| H | -4.34630 | -0.20351 | 2.93209 |
| H | -6.32886 | 0.55520 | 1.67177 |
| O | -2.06271 | -0.87501 | 1.92679 |
| Na | -0.11198 | 0.19909 | 1.72317 |

HL Ni(II)

ΔG = -2595.691069

| | | | |
|---|---|---|---|
| C | -1.81019 | -0.04967 | -1.33722 |
| C | -0.97339 | 0.18746 | -3.59197 |
| C | 0.32986 | 0.12695 | -3.10078 |
| H | -1.15104 | 0.31680 | -4.65267 |
| C | 0.51764 | -0.03288 | -1.73644 |
| H | 1.18382 | 0.21548 | -3.75889 |
| C | 1.92124 | -0.04553 | -1.13489 |
| H | 2.62699 | -0.04720 | -1.96291 |
| C | 3.09560 | 2.17979 | -0.74322 |
| C | 3.31011 | 3.31085 | 0.04005 |
| H | 3.61836 | 2.04652 | -1.68196 |
| C | 1.73271 | 2.43794 | 1.60987 |
| C | 2.61690 | 3.44528 | 1.23924 |
| H | 4.00848 | 4.07354 | -0.28316 |
| H | 1.16960 | 2.49885 | 2.53415 |
| H | 2.75578 | 4.30739 | 1.87884 |
| C | 2.15431 | -1.29331 | -0.31168 |

| | | | |
|---|---|---|---|
| C | 3.34594 | -3.29035 | 0.27172 |
| C | 1.42184 | -2.57119 | 1.49543 |
| C | 2.44276 | -3.49010 | 1.31373 |
| H | 4.15286 | -3.99265 | 0.10217 |
| H | 0.69093 | -2.68070 | 2.28732 |
| H | 2.51958 | -4.34286 | 1.97475 |
| C | 2.19280 | 1.21628 | -0.29814 |
| C | 3.20100 | -2.17869 | -0.55156 |
| H | 3.88651 | -1.99410 | -1.36825 |
| N | -0.53201 | -0.15542 | -0.89547 |
| N | 1.52863 | 1.34711 | 0.86032 |
| N | 1.28805 | -1.49778 | 0.69526 |
| C | -2.03946 | 0.11821 | -2.71237 |
| H | -3.05165 | 0.22340 | -3.07551 |
| C | -2.91120 | -0.05575 | -0.36323 |
| C | -4.20304 | -0.45979 | -0.74902 |
| C | -2.70775 | 0.42038 | 0.96505 |
| C | -5.28196 | -0.38044 | 0.11741 |
| H | -4.35651 | -0.85981 | -1.74423 |
| C | -3.82235 | 0.51858 | 1.82366 |
| C | -5.08485 | 0.12760 | 1.40740 |
| H | -6.26255 | -0.71154 | -0.20157 |
| H | -3.65765 | 0.90161 | 2.82402 |
| H | -5.92054 | 0.20422 | 2.09450 |
| O | -1.52039 | 0.81100 | 1.41979 |
| Ni | -0.09763 | -0.21265 | 0.96915 |

HL V(II)

ΔG = -2031.393522

| | | | |
|---|---|---|---|
| C | -2.24791 | 0.75281 | -1.21152 |
| C | -1.42338 | 1.39728 | -3.38327 |
| C | -0.08955 | 1.33642 | -2.94750 |
| H | -1.63080 | 1.68952 | -4.40746 |
| C | 0.12743 | 0.94961 | -1.61900 |
| H | 0.72192 | 1.59090 | -3.61761 |
| C | 3.17594 | -1.11686 | -0.32491 |
| H | 4.05429 | -1.59278 | -0.74262 |
| C | 4.72682 | 0.69688 | 0.15950 |
| C | 5.01410 | 1.92913 | 0.71663 |
| H | 5.50852 | 0.09208 | -0.28126 |
| C | 2.69028 | 2.14796 | 1.24259 |
| C | 3.97759 | 2.66840 | 1.28810 |
| H | 6.02780 | 2.31018 | 0.71119 |
| H | 1.85995 | 2.72015 | 1.64403 |
| H | 4.14991 | 3.63432 | 1.74315 |
| C | 2.03517 | -1.96027 | -0.25108 |
| C | 1.15402 | -4.21237 | -0.48803 |

| | | | |
|---|---|---|---|
| C | -0.17590 | -2.41460 | 0.36822 |
| C | -0.05664 | -3.75678 | 0.04109 |
| H | 1.27891 | -5.24950 | -0.77340 |
| H | -1.10587 | -2.02239 | 0.75724 |
| H | -0.89972 | -4.41869 | 0.18632 |
| C | 3.40365 | 0.19726 | 0.17533 |
| C | 2.19331 | -3.31636 | -0.63280 |
| H | 3.15092 | -3.63639 | -1.02226 |
| N | -0.94533 | 0.66740 | -0.91738 |
| N | 2.39295 | 0.95743 | 0.69790 |
| N | 0.82088 | -1.52187 | 0.21730 |
| C | -2.50765 | 1.12633 | -2.53850 |
| H | -3.51999 | 1.22845 | -2.90603 |
| C | -3.14785 | 0.49109 | -0.08898 |
| C | -4.54083 | 0.42084 | -0.26920 |
| C | -2.60509 | 0.33254 | 1.23097 |
| C | -5.40408 | 0.20449 | 0.79263 |
| H | -4.94616 | 0.53553 | -1.26812 |
| C | -3.50713 | 0.11551 | 2.29323 |
| C | -4.87540 | 0.05319 | 2.08106 |
| H | -6.47314 | 0.15248 | 0.62631 |
| H | -3.09390 | 0.00046 | 3.28871 |
| H | -5.53797 | -0.11558 | 2.92299 |
| O | -1.30401 | 0.39152 | 1.52607 |
| V | 0.32637 | 0.47710 | 0.48428 |

HL V(III)

ΔG = -2031.169819

| | | | |
|---|---|---|---|
| C | 0.18402 | 0.63359 | 3.38651 |
| C | -0.77247 | -0.14856 | 1.30564 |
| C | 0.47611 | -0.24365 | 0.67392 |
| H | -1.65839 | -0.42396 | 0.74511 |
| C | 1.56437 | 0.13284 | 1.47258 |
| H | 0.57605 | -0.58623 | -0.34565 |
| C | 5.10231 | 0.06579 | 0.76350 |
| H | 5.40021 | -0.23227 | -0.23435 |
| C | 5.51381 | 2.38100 | 0.00182 |
| C | 5.59736 | 3.71178 | 0.32097 |
| H | 5.85851 | 2.00229 | -0.95128 |
| C | 4.59290 | 3.27670 | 2.46177 |
| C | 5.16364 | 4.16474 | 1.58963 |
| H | 6.00741 | 4.41709 | -0.39074 |
| H | 4.18844 | 3.56893 | 3.42151 |
| H | 5.25332 | 5.20556 | 1.86953 |
| C | 4.99657 | -0.99440 | 1.69401 |
| C | 5.54270 | -3.23799 | 2.38095 |
| C | 4.52418 | -1.70314 | 3.92777 |
| C | 5.09002 | -2.92574 | 3.68552 |
| H | 5.94997 | -4.21937 | 2.17324 |
| H | 4.10587 | -1.42408 | 4.88522 |
| H | 5.16049 | -3.64887 | 4.48661 |
| C | 5.02128 | 1.46412 | 0.96813 |
| C | 5.48135 | -2.29412 | 1.38905 |
| H | 5.84131 | -2.49577 | 0.38884 |
| N | 1.30857 | 0.52469 | 2.67267 |
| N | 4.48208 | 1.95597 | 2.14326 |
| N | 4.43676 | -0.76706 | 2.93959 |
| C | -0.94382 | 0.27571 | 2.63994 |
| H | -1.93639 | 0.31081 | 3.06825 |
| C | 0.41224 | 1.07145 | 4.75660 |
| C | -0.62456 | 1.26525 | 5.67744 |
| C | 1.75804 | 1.29941 | 5.18637 |
| C | -0.35709 | 1.67046 | 6.97823 |
| H | -1.64856 | 1.09526 | 5.36858 |
| C | 2.01269 | 1.70779 | 6.50075 |
| C | 0.96204 | 1.89150 | 7.38779 |
| H | -1.17328 | 1.81370 | 7.67506 |
| H | 3.04007 | 1.87156 | 6.80018 |
| H | 1.16968 | 2.20669 | 8.40304 |
| O | 2.84721 | 1.13699 | 4.38917 |
| V | 3.17500 | 0.64932 | 2.68924 |

HL Zn(II)

ΔG = -2866.763982

| | | | |
|---|---|---|---|
| C | -1.94475 | -0.63744 | -0.76869 |
| C | -1.27179 | -1.90409 | -2.72145 |
| C | 0.06729 | -1.67647 | -2.40133 |
| H | -1.53009 | -2.48391 | -3.59975 |
| C | 0.33834 | -0.88290 | -1.29790 |
| H | 0.86887 | -2.08375 | -3.00302 |
| C | 1.77988 | -0.52584 | -0.91902 |
| H | 2.43248 | -1.12717 | -1.54842 |
| C | 3.04127 | 1.29332 | -2.17763 |
| C | 3.27070 | 2.64169 | -2.43516 |
| H | 3.59543 | 0.52518 | -2.70107 |
| C | 1.59880 | 3.17512 | -0.81478 |
| C | 2.53744 | 3.60133 | -1.74317 |
| H | 4.01229 | 2.93735 | -3.16735 |
| H | 1.00219 | 3.88042 | -0.25003 |
| H | 2.68452 | 4.65929 | -1.91508 |
| C | 2.11716 | -0.87120 | 0.53099 |
| C | 3.36765 | -2.11437 | 2.16146 |
| C | 1.74636 | -0.47913 | 2.79487 |
| C | 2.69227 | -1.42484 | 3.16397 |

| | | | |
|---|---|---|---|
| H | 4.11258 | -2.86024 | 2.41090 |
| H | 1.19179 | 0.08392 | 3.53517 |
| H | 2.88922 | -1.61090 | 4.21143 |
| C | 2.08234 | 0.93823 | -1.23310 |
| C | 3.07729 | -1.83447 | 0.82921 |
| H | 3.58831 | -2.35396 | 0.02894 |
| N | -0.63880 | -0.37404 | -0.52794 |
| N | 1.37902 | 1.87303 | -0.56752 |
| N | 1.46727 | -0.21009 | 1.50928 |
| C | -2.27243 | -1.38505 | -1.91893 |
| H | -3.30521 | -1.54793 | -2.18809 |
| C | -2.98874 | -0.17477 | 0.17595 |
| C | -4.22037 | -0.87160 | 0.16844 |
| C | -2.84413 | 0.91496 | 1.10126 |
| C | -5.29185 | -0.52697 | 0.97292 |
| H | -4.32883 | -1.73546 | -0.47445 |
| C | -3.96923 | 1.26125 | 1.89367 |
| C | -5.16232 | 0.56740 | 1.83742 |
| H | -6.20842 | -1.10315 | 0.93661 |
| H | -3.84745 | 2.10107 | 2.56869 |
| H | -5.98883 | 0.86418 | 2.47455 |
| O | -1.76293 | 1.65472 | 1.27058 |
| Zn | 0.00396 | 1.10734 | 0.80495 |

HL-NH2

ΔG = -1198.346393

| | | | |
|---|---|---|---|
| C | 1.87302 | 0.98604 | 0.96774 |
| C | 0.70793 | 1.95571 | 2.84369 |
| C | -0.41193 | 1.21679 | 2.48521 |
| H | 0.68251 | 2.60960 | 3.70884 |
| C | -0.33459 | 0.39026 | 1.35736 |
| H | -1.32842 | 1.27520 | 3.06101 |
| C | -1.57283 | -0.42201 | 0.95959 |
| H | -1.97900 | -0.83215 | 1.88778 |
| C | -3.99248 | 0.28115 | 0.75577 |
| C | -4.97098 | 1.09625 | 0.17938 |
| H | -4.25254 | -0.46843 | 1.49256 |
| C | -3.24400 | 2.16100 | -1.09258 |
| C | -4.60795 | 2.04791 | -0.75632 |
| H | -6.01110 | 0.98757 | 0.46637 |
| H | -5.34332 | 2.69463 | -1.21970 |
| C | -1.23973 | -1.64033 | 0.10255 |
| C | -0.96995 | -2.80789 | -1.96925 |
| C | -0.55923 | -3.85238 | 0.14361 |
| C | -0.58969 | -3.93463 | -1.26178 |
| H | -1.00741 | -2.83737 | -3.05279 |
| H | -0.32469 | -4.85678 | -1.76525 |
| C | -2.66742 | 0.46006 | 0.36219 |
| C | -1.30260 | -1.63407 | -1.28763 |
| H | -1.59130 | -0.74013 | -1.82179 |
| N | 0.77569 | 0.27533 | 0.62718 |
| N | -2.30216 | 1.38528 | -0.53834 |
| N | -0.88448 | -2.73188 | 0.80210 |
| C | 1.86337 | 1.84526 | 2.07572 |
| H | 2.74986 | 2.41615 | 2.32343 |
| C | 3.11302 | 0.79902 | 0.16900 |
| C | 4.27973 | 0.40592 | 0.83560 |
| C | 3.13153 | 1.04964 | -1.25913 |
| C | 5.48838 | 0.18954 | 0.17168 |
| H | 4.23065 | 0.24210 | 1.90925 |
| C | 4.39201 | 0.79945 | -1.90301 |
| C | 5.52363 | 0.38750 | -1.21528 |
| H | 6.36934 | -0.13247 | 0.71513 |
| H | 4.43665 | 0.96494 | -2.97627 |
| H | 6.44866 | 0.22084 | -1.76153 |
| O | 2.12087 | 1.49557 | -1.91264 |
| N | -0.14279 | -4.92468 | 0.91388 |
| H | -0.41487 | -4.88073 | 1.88591 |
| N | -2.81945 | 3.05536 | -2.05658 |
| H | -1.83140 | 3.26516 | -2.04204 |
| H | -0.23351 | -5.84336 | 0.50522 |
| H | -3.41020 | 3.85354 | -2.23744 |

HL-NH2 Al(III)

ΔG = -1440.51567

| | | | |
|---|---|---|---|
| C | 1.85971 | -0.05368 | 1.47278 |
| C | 1.08015 | -0.14758 | 3.76276 |
| C | -0.25298 | -0.11383 | 3.33766 |
| H | 1.30292 | -0.19750 | 4.82151 |
| C | -0.48238 | -0.05651 | 1.98147 |
| H | -1.07243 | -0.13498 | 4.04253 |
| C | -1.87555 | -0.02599 | 1.33619 |
| H | -2.61282 | -0.04244 | 2.13369 |
| C | -3.08125 | 2.16090 | 0.85713 |
| C | -3.22618 | 3.31041 | 0.05150 |
| H | -3.72490 | 1.98891 | 1.70808 |
| C | -1.39283 | 2.57469 | -1.32160 |
| C | -2.40100 | 3.52140 | -1.02244 |
| H | -3.99580 | 4.03522 | 0.28675 |
| H | -2.49788 | 4.40020 | -1.64560 |
| C | -2.12043 | -1.26561 | 0.46463 |
| C | -3.26717 | -3.26861 | -0.13582 |
| C | -1.39974 | -2.49709 | -1.44130 |
| C | -2.42663 | -3.44108 | -1.20486 |

| | | | |
|---|---|---|---|
| H | -4.05127 | -3.99186 | 0.05143 |
| H | -2.52382 | -4.28905 | -1.86932 |
| C | -2.10323 | 1.26208 | 0.53388 |
| C | -3.11681 | -2.16372 | 0.72922 |
| H | -3.76896 | -2.02621 | 1.58000 |
| N | 0.55148 | -0.02771 | 1.10371 |
| N | -1.27045 | 1.46235 | -0.54761 |
| N | -1.27761 | -1.42308 | -0.61516 |
| C | 2.12273 | -0.12046 | 2.85160 |
| H | 3.13558 | -0.15436 | 3.21937 |
| C | 2.91177 | -0.01241 | 0.42926 |
| C | 4.26419 | 0.00509 | 0.83893 |
| C | 2.66935 | 0.01151 | -0.97709 |
| C | 5.31653 | 0.03481 | -0.05873 |
| H | 4.50281 | -0.00074 | 1.89250 |
| C | 3.73960 | 0.03880 | -1.87512 |
| C | 5.05152 | 0.04915 | -1.42969 |
| H | 6.33545 | 0.04738 | 0.30715 |
| H | 3.50532 | 0.05256 | -2.93238 |
| H | 5.86343 | 0.07067 | -2.14701 |
| O | 1.42747 | 0.00855 | -1.55072 |
| N | -0.55085 | -2.62393 | -2.47683 |
| H | 0.27342 | -2.04855 | -2.57799 |
| N | -0.56371 | 2.73759 | -2.36790 |
| H | 0.24157 | 2.14785 | -2.52244 |
| H | -0.59957 | -3.44318 | -3.06204 |
| H | -0.60748 | 3.58586 | -2.91067 |
| Al | -0.04336 | 0.01859 | -0.67119 |

HL-NH2 Be(II)

ΔG = -1213.012537

| | | | |
|---|---|---|---|
| C | -1.71905 | -0.00035 | 1.51258 |
| C | -0.92420 | -0.00079 | 3.79658 |
| C | 0.39921 | -0.00061 | 3.34473 |
| H | -1.13074 | -0.00103 | 4.86023 |
| C | 0.60230 | -0.00032 | 1.97801 |
| H | 1.23396 | -0.00070 | 4.03256 |
| C | 1.96991 | -0.00011 | 1.29945 |
| H | 2.75265 | -0.00018 | 2.05362 |
| C | 3.08430 | -2.19676 | 0.60926 |
| C | 3.10025 | -3.29653 | -0.26778 |
| H | 3.81191 | -2.09752 | 1.40278 |
| C | 1.19084 | -2.38808 | -1.41437 |
| C | 2.16712 | -3.40192 | -1.27348 |
| H | 3.85320 | -4.06715 | -0.15121 |
| H | 2.16601 | -4.24003 | -1.95831 |
| C | 2.10588 | 1.24642 | 0.42554 |
| C | 3.09980 | 3.29696 | -0.26674 |
| C | 1.19056 | 2.38858 | -1.41366 |
| C | 2.16668 | 3.40252 | -1.27243 |
| H | 3.85263 | 4.06766 | -0.14992 |
| H | 2.16547 | 4.24083 | -1.95702 |
| C | 2.10606 | -1.24635 | 0.42515 |
| C | 3.08398 | 2.19692 | 0.60996 |
| H | 3.81160 | 2.09755 | 1.40347 |
| N | -0.42864 | -0.00021 | 1.11261 |
| N | 1.17767 | -1.34038 | -0.56120 |
| N | 1.17750 | 1.34062 | -0.56080 |
| C | -1.97685 | -0.00066 | 2.89600 |
| H | -2.98952 | -0.00082 | 3.26910 |
| C | -2.76575 | -0.00015 | 0.47179 |
| C | -4.12656 | -0.00014 | 0.84432 |
| C | -2.45544 | 0.00003 | -0.92247 |
| C | -5.15094 | 0.00001 | -0.08713 |
| H | -4.39311 | -0.00023 | 1.89285 |
| C | -3.51068 | 0.00017 | -1.85408 |
| C | -4.83502 | 0.00015 | -1.45036 |
| H | -6.18300 | 0.00002 | 0.24141 |
| H | -3.24825 | 0.00029 | -2.90578 |
| H | -5.62397 | 0.00026 | -2.19428 |
| O | -1.20926 | 0.00009 | -1.41228 |
| N | 0.26817 | 2.40789 | -2.40475 |
| H | -0.49042 | 1.73641 | -2.38383 |
| N | 0.26841 | -2.40725 | -2.40542 |
| H | -0.49024 | -1.73585 | -2.38431 |
| H | 0.15460 | 3.23936 | -2.96127 |
| H | 0.15499 | -3.23852 | -2.96224 |
| Be | 0.05251 | 0.00005 | -0.51446 |

HL-NH2 B(III)

ΔG = -1222.878491

| | | | |
|---|---|---|---|
| C | -1.61106 | 0.00120 | 1.57232 |
| C | -0.77955 | 0.00246 | 3.84198 |
| C | 0.53761 | 0.00187 | 3.37200 |
| H | -0.97262 | 0.00317 | 4.90739 |
| C | 0.72863 | 0.00103 | 2.01080 |
| H | 1.38395 | 0.00207 | 4.04439 |
| C | 2.05665 | 0.00025 | 1.28462 |
| H | 2.88611 | 0.00039 | 1.98440 |
| C | 3.03928 | -2.21306 | 0.48117 |
| C | 2.94507 | -3.29430 | -0.42158 |
| H | 3.82634 | -2.15660 | 1.21937 |
| C | 0.99345 | -2.27736 | -1.42771 |
| C | 1.94632 | -3.32714 | -1.35539 |

| | | | |
|---|---|---|---|
| H | 3.66958 | -4.09807 | -0.37834 |
| H | 1.86244 | -4.14126 | -2.06269 |
| C | 2.10036 | 1.22952 | 0.39470 |
| C | 2.94693 | 3.29255 | -0.42492 |
| C | 0.99472 | 2.27569 | -1.43002 |
| C | 1.94818 | 3.32502 | -1.35874 |
| H | 3.67188 | 4.09596 | -0.38248 |
| H | 1.86474 | 4.13849 | -2.06684 |
| C | 2.09966 | -1.22997 | 0.39598 |
| C | 3.04055 | 2.21215 | 0.47889 |
| H | 3.82759 | 2.15598 | 1.21713 |
| N | -0.31835 | 0.00077 | 1.14801 |
| N | 1.07597 | -1.25433 | -0.52552 |
| N | 1.07666 | 1.25353 | -0.52679 |
| C | -1.84102 | 0.00212 | 2.95365 |
| H | -2.85230 | 0.00260 | 3.32908 |
| C | -2.66182 | 0.00065 | 0.55721 |
| C | -4.02898 | 0.00068 | 0.89454 |
| C | -2.32335 | 0.00005 | -0.80639 |
| C | -5.00852 | 0.00023 | -0.08477 |
| H | -4.33100 | 0.00102 | 1.93304 |
| C | -3.30211 | -0.00037 | -1.79544 |
| C | -4.64320 | -0.00026 | -1.43373 |
| H | -6.05327 | 0.00027 | 0.19869 |
| H | -3.00111 | -0.00079 | -2.83582 |
| H | -5.40379 | -0.00059 | -2.20508 |
| O | -1.01573 | -0.00017 | -1.22981 |
| N | 0.04087 | 2.29017 | -2.36819 |
| H | -0.62392 | 1.53854 | -2.45738 |
| N | 0.03954 | -2.29224 | -2.36582 |
| H | -0.62475 | -1.54027 | -2.45581 |
| H | 0.01106 | 3.04751 | -3.03201 |
| H | 0.00930 | -3.05020 | -3.02891 |
| B | 0.10120 | -0.00005 | -0.36330 |

HL-NH2 Ca(II)

ΔG = -1875.874369

| | | | |
|---|---|---|---|
| C | -1.96864 | 0.04572 | 1.30570 |
| C | -1.11269 | 0.08773 | 3.56529 |
| C | 0.17525 | -0.04025 | 3.05193 |
| H | -1.27167 | 0.16475 | 4.63470 |
| C | 0.33790 | -0.11406 | 1.67096 |
| H | 1.03644 | -0.06006 | 3.70735 |
| C | 1.76330 | -0.12714 | 1.10914 |
| H | 2.41150 | -0.07151 | 1.98192 |
| C | 3.37571 | -2.01540 | 0.79890 |
| C | 3.79219 | -3.17991 | 0.14810 |
| H | 3.96323 | -1.58821 | 1.60035 |
| C | 1.80708 | -3.06422 | -1.18964 |
| C | 3.00750 | -3.71745 | -0.84965 |
| H | 4.71608 | -3.66730 | 0.43714 |
| H | 3.28643 | -4.63207 | -1.35780 |
| C | 2.06711 | 1.14670 | 0.31134 |
| C | 3.33988 | 3.14288 | -0.03734 |
| C | 1.38784 | 2.64621 | -1.33493 |
| C | 2.46315 | 3.50754 | -1.04024 |
| H | 4.16976 | 3.79206 | 0.21753 |
| H | 2.57360 | 4.44314 | -1.57417 |
| C | 2.19066 | -1.41238 | 0.39629 |
| C | 3.13311 | 1.95675 | 0.67750 |
| H | 3.78011 | 1.68101 | 1.49956 |
| N | -0.71029 | -0.10799 | 0.82777 |
| N | 1.42518 | -1.91218 | -0.59820 |
| N | 1.24689 | 1.46264 | -0.70711 |
| C | -2.18442 | 0.15704 | 2.69208 |
| H | -3.18308 | 0.32246 | 3.07131 |
| C | -3.12430 | 0.13312 | 0.37701 |
| C | -4.36552 | -0.37720 | 0.80808 |
| C | -3.05425 | 0.79176 | -0.90046 |
| C | -5.52995 | -0.23995 | 0.06648 |
| H | -4.40952 | -0.91456 | 1.74893 |
| C | -4.27233 | 0.95416 | -1.61419 |
| C | -5.47594 | 0.45079 | -1.15098 |
| H | -6.46110 | -0.66165 | 0.42579 |
| H | -4.22129 | 1.47863 | -2.56266 |
| H | -6.37665 | 0.58402 | -1.74188 |
| O | -1.93209 | 1.23096 | -1.42149 |
| N | 0.44203 | 2.95024 | -2.28653 |
| H | -0.49314 | 2.58459 | -2.11466 |
| N | 1.00621 | -3.57651 | -2.18661 |
| H | 0.01388 | -3.40590 | -2.11905 |
| H | 0.45270 | 3.89442 | -2.64351 |
| H | 1.21971 | -4.51631 | -2.48711 |
| Ca | -0.28866 | -0.27771 | -1.58748 |

HL-NH2 Co(II)

ΔG = -2580.921115

| | | | |
|---|---|---|---|
| C | 1.87922 | -0.36894 | 1.43399 |
| C | 1.13051 | -0.44466 | 3.73253 |
| C | -0.18910 | -0.35612 | 3.29230 |
| H | 1.35071 | -0.49152 | 4.79229 |
| C | -0.42508 | -0.28312 | 1.92783 |
| H | -1.01472 | -0.32289 | 3.99065 |
| C | -1.83611 | -0.09001 | 1.38190 |

| | | | |
|---|---|---|---|
| H | -2.51812 | -0.11458 | 2.22922 |
| C | -2.89250 | 2.21084 | 1.17712 |
| C | -2.97602 | 3.43899 | 0.50567 |
| H | -3.51500 | 1.99512 | 2.03490 |
| C | -1.23515 | 2.70507 | -0.98070 |
| C | -2.15202 | 3.69641 | -0.56894 |
| H | -3.67995 | 4.19255 | 0.83912 |
| H | -2.18627 | 4.64425 | -1.09115 |
| C | -2.24488 | -1.19954 | 0.43058 |
| C | -3.69258 | -2.96863 | -0.25768 |
| C | -1.79281 | -2.30758 | -1.57875 |
| C | -2.91951 | -3.13372 | -1.38479 |
| H | -4.55613 | -3.60252 | -0.09513 |
| H | -3.15237 | -3.89137 | -2.12192 |
| C | -1.98699 | 1.27641 | 0.70928 |
| C | -3.35383 | -1.98388 | 0.67893 |
| H | -3.94094 | -1.83207 | 1.57408 |
| N | 0.58428 | -0.32928 | 1.03365 |
| N | -1.19373 | 1.50842 | -0.35830 |
| N | -1.47646 | -1.34904 | -0.67606 |
| C | 2.16168 | -0.42945 | 2.80941 |
| H | 3.18781 | -0.43127 | 3.14706 |
| C | 2.95791 | -0.29041 | 0.43075 |
| C | 4.23225 | -0.80798 | 0.73798 |
| C | 2.77711 | 0.37148 | -0.82169 |
| C | 5.31380 | -0.66176 | -0.11571 |
| H | 4.37035 | -1.35211 | 1.66450 |
| C | 3.89865 | 0.53706 | -1.66301 |
| C | 5.14194 | 0.03356 | -1.31903 |
| H | 6.27654 | -1.08255 | 0.14739 |
| H | 3.74932 | 1.06222 | -2.59949 |
| H | 5.97956 | 0.16786 | -1.99494 |
| O | 1.60974 | 0.85694 | -1.23157 |
| N | -1.03382 | -2.43389 | -2.70815 |
| H | -0.07927 | -2.10872 | -2.70673 |
| N | -0.38795 | 2.89698 | -2.03580 |
| H | 0.46411 | 2.34342 | -2.03888 |
| H | -1.18110 | -3.26137 | -3.26650 |
| H | -0.28399 | 3.84304 | -2.37053 |
| Co | 0.06024 | -0.08628 | -0.83025 |

HL-NH2 Co(III)

ΔG = -2580.701621

| | | | |
|---|---|---|---|
| C | 1.85148 | -0.00029 | 1.44132 |
| C | 1.07509 | -0.00077 | 3.74702 |
| C | -0.24193 | -0.00058 | 3.29764 |
| H | 1.29543 | -0.00102 | 4.80695 |
| C | -0.47968 | -0.00030 | 1.93758 |
| H | -1.07497 | -0.00068 | 3.98686 |
| C | -1.91071 | -0.00013 | 1.41940 |
| H | -2.57941 | -0.00022 | 2.27573 |
| C | -3.16573 | 2.14887 | 0.86790 |
| C | -3.30804 | 3.24914 | 0.00483 |
| H | -3.80602 | 2.01545 | 1.72844 |
| C | -1.44527 | 2.44898 | -1.29993 |
| C | -2.46067 | 3.40418 | -1.06578 |
| H | -4.08047 | 3.98552 | 0.18983 |
| H | -2.54117 | 4.25331 | -1.73186 |
| C | -2.17654 | -1.23777 | 0.58557 |
| C | -3.30845 | -3.24880 | 0.00384 |
| C | -1.44544 | -2.44858 | -1.30055 |
| C | -2.46101 | -3.40370 | -1.06673 |
| H | -4.08101 | -3.98512 | 0.18859 |
| H | -2.54155 | -4.25266 | -1.73303 |
| C | -2.17638 | 1.23779 | 0.58594 |
| C | -3.16604 | -2.14879 | 0.86722 |
| H | -3.80635 | -2.01552 | 1.72777 |
| N | 0.54152 | -0.00017 | 1.03666 |
| N | -1.35069 | 1.37416 | -0.47729 |
| N | -1.35082 | -1.37396 | -0.47766 |
| C | 2.10075 | -0.00063 | 2.82783 |
| H | 3.11625 | -0.00079 | 3.18655 |
| C | 2.98364 | -0.00010 | 0.49633 |
| C | 4.31642 | -0.00009 | 0.95871 |
| C | 2.80575 | 0.00008 | -0.91327 |
| C | 5.39448 | 0.00005 | 0.08892 |
| H | 4.53333 | -0.00018 | 2.01572 |
| C | 3.90155 | 0.00021 | -1.79321 |
| C | 5.18987 | 0.00019 | -1.29624 |
| H | 6.39968 | 0.00005 | 0.49119 |
| H | 3.69839 | 0.00034 | -2.85656 |
| H | 6.03420 | 0.00030 | -1.97473 |
| O | 1.59256 | 0.00012 | -1.45128 |
| N | -0.59311 | -2.56866 | -2.34696 |
| H | 0.28336 | -2.06647 | -2.35892 |
| N | -0.59309 | 2.56914 | -2.34646 |
| H | 0.28332 | 2.06686 | -2.35864 |
| H | -0.59836 | -3.43347 | -2.86588 |
| H | -0.59830 | 3.43406 | -2.86520 |
| Co | -0.01824 | 0.00007 | -0.75284 |

HL-NH2 Cr(II)

ΔG = -2242.635701

| | | | |
|---|---|---|---|
| C | -1.86986 | -0.44346 | 1.39430 |

| | | | |
|---|---|---|---|
| C | -1.09025 | -0.57400 | 3.68869 |
| C | 0.22002 | -0.43424 | 3.23257 |
| H | -1.29929 | -0.66456 | 4.74744 |
| C | 0.44202 | -0.29206 | 1.87367 |
| H | 1.05470 | -0.40416 | 3.92105 |
| C | 1.83901 | 0.00281 | 1.34177 |
| H | 2.50503 | 0.04161 | 2.20125 |
| C | 3.54110 | -1.75316 | 0.72753 |
| C | 4.01350 | -2.71784 | -0.17168 |
| H | 4.07501 | -1.52779 | 1.64048 |
| C | 2.11030 | -2.27466 | -1.57480 |
| C | 3.30486 | -2.98109 | -1.32173 |
| H | 4.92950 | -3.25769 | 0.03705 |
| H | 3.63986 | -3.72525 | -2.03295 |
| C | 1.87941 | 1.38168 | 0.68431 |
| C | 2.71769 | 3.61038 | 0.49365 |
| C | 0.94730 | 2.81038 | -0.92531 |
| C | 1.81759 | 3.84477 | -0.52413 |
| H | 3.38987 | 4.39813 | 0.81291 |
| H | 1.75760 | 4.80904 | -1.01277 |
| C | 2.37582 | -1.08061 | 0.41713 |
| C | 2.74901 | 2.36023 | 1.12689 |
| H | 3.42976 | 2.16057 | 1.94336 |
| N | -0.57139 | -0.34558 | 0.96542 |
| N | 1.66631 | -1.32509 | -0.71544 |
| N | 1.01653 | 1.58858 | -0.34074 |
| C | -2.12717 | -0.54690 | 2.76857 |
| H | -3.14911 | -0.58462 | 3.11667 |
| C | -2.97914 | -0.41037 | 0.41666 |
| C | -4.22406 | -0.97550 | 0.76734 |
| C | -2.87203 | 0.22182 | -0.85873 |
| C | -5.33627 | -0.89444 | -0.05519 |
| H | -4.31501 | -1.50875 | 1.70542 |
| C | -4.02069 | 0.31765 | -1.67175 |
| C | -5.23305 | -0.22502 | -1.28045 |
| H | -6.27097 | -1.34975 | 0.24916 |
| H | -3.91728 | 0.82299 | -2.62534 |
| H | -6.09542 | -0.14258 | -1.93306 |
| O | -1.73147 | 0.73399 | -1.32177 |
| N | 0.03826 | 2.98368 | -1.92772 |
| H | -0.77639 | 2.37502 | -1.93109 |
| N | 1.41420 | -2.51605 | -2.72675 |
| H | 0.42902 | -2.30872 | -2.77935 |
| H | -0.12390 | 3.92880 | -2.24040 |
| H | 1.68297 | -3.33185 | -3.25609 |
| Cr | -0.05300 | -0.08247 | -0.91953 |

HL-NH2 Cr(III)

ΔG = -2242.495839

| | | | |
|---|---|---|---|
| C | -1.86913 | 0.76462 | 1.26688 |
| C | -1.09389 | 1.33131 | 3.49181 |
| C | 0.21097 | 1.01648 | 3.11449 |
| H | -1.30570 | 1.65725 | 4.50264 |
| C | 0.43554 | 0.59068 | 1.82021 |
| H | 1.03313 | 1.08355 | 3.81371 |
| C | 1.82193 | 0.16255 | 1.36182 |
| H | 2.47927 | 0.23579 | 2.22404 |
| C | 2.66131 | -2.21933 | 1.47994 |
| C | 2.60755 | -3.54349 | 1.00788 |
| H | 3.34340 | -1.93065 | 2.26719 |
| C | 0.83656 | -2.91088 | -0.49946 |
| C | 1.70701 | -3.89121 | 0.03381 |
| H | 3.26633 | -4.29395 | 1.42748 |
| H | 1.62616 | -4.90861 | -0.32570 |
| C | 2.39329 | 1.07443 | 0.28603 |
| C | 4.03006 | 2.62038 | -0.50155 |
| C | 2.20673 | 1.89418 | -1.90457 |
| C | 3.37991 | 2.66584 | -1.70580 |
| H | 4.92515 | 3.20982 | -0.34438 |
| H | 3.74146 | 3.28223 | -2.51854 |
| C | 1.82234 | -1.29121 | 0.91579 |
| C | 3.53043 | 1.80417 | 0.52843 |
| H | 4.02440 | 1.74506 | 1.48798 |
| N | -0.57501 | 0.50243 | 0.92060 |
| N | 0.94443 | -1.61932 | -0.07631 |
| N | 1.72557 | 1.10529 | -0.90444 |
| C | -2.12391 | 1.18696 | 2.58257 |
| H | -3.14042 | 1.36625 | 2.89683 |
| C | -2.97505 | 0.54918 | 0.31672 |
| C | -4.23190 | 1.14511 | 0.55397 |
| C | -2.85609 | -0.31230 | -0.80515 |
| C | -5.33280 | 0.87080 | -0.23919 |
| H | -4.34436 | 1.85086 | 1.36607 |
| C | -3.98381 | -0.60929 | -1.58511 |
| C | -5.20964 | -0.02830 | -1.30410 |
| H | -6.28031 | 1.35248 | -0.03267 |
| H | -3.86095 | -1.28920 | -2.41924 |
| H | -6.06777 | -0.26085 | -1.92410 |
| O | -1.68524 | -0.86328 | -1.16195 |
| N | 1.59610 | 1.94897 | -3.10818 |
| H | 0.71902 | 1.51019 | -3.32670 |
| N | -0.07987 | -3.22775 | -1.43828 |
| H | -0.88372 | -2.62512 | -1.58279 |
| H | 1.94890 | 2.58941 | -3.80121 |
| H | -0.19574 | -4.20094 | -1.67524 |

| | | | |
|---|---|---|---|
| Cr | -0.02622 | -0.06911 | -0.90775 |

HL-NH2 Cu(II)

ΔG = -2838.629298

| | | | |
|---|---|---|---|
| C | -1.90166 | 0.02013 | 1.49066 |
| C | -1.13010 | -0.30803 | 3.75718 |
| C | 0.19046 | -0.24288 | 3.31188 |
| H | -1.34086 | -0.47298 | 4.80707 |
| C | 0.41512 | -0.04660 | 1.95712 |
| H | 1.02094 | -0.36401 | 3.99461 |
| C | 1.82956 | -0.06721 | 1.37179 |
| H | 2.51847 | -0.12927 | 2.21132 |
| C | 3.00018 | -2.26725 | 0.86931 |
| C | 3.14284 | -3.39377 | 0.04547 |
| H | 3.60330 | -2.14187 | 1.75836 |
| C | 1.39127 | -2.54612 | -1.36104 |
| C | 2.34261 | -3.54668 | -1.06696 |
| H | 3.87484 | -4.15423 | 0.29135 |
| H | 2.42280 | -4.41827 | -1.70420 |
| C | 2.17766 | 1.20076 | 0.60493 |
| C | 3.53425 | 3.13030 | 0.22098 |
| C | 1.67659 | 2.60498 | -1.20943 |
| C | 2.76074 | 3.44283 | -0.87374 |
| H | 4.36561 | 3.76898 | 0.49455 |
| H | 2.96037 | 4.31964 | -1.47605 |
| C | 2.05819 | -1.31912 | 0.51183 |
| C | 3.24383 | 1.99099 | 0.98465 |
| H | 3.83733 | 1.72931 | 1.84961 |
| N | -0.61175 | 0.11288 | 1.10270 |
| N | 1.29244 | -1.44721 | -0.59053 |
| N | 1.41202 | 1.50130 | -0.47443 |
| C | -2.17367 | -0.19815 | 2.85310 |
| H | -3.19341 | -0.30608 | 3.19288 |
| C | -2.97626 | 0.11386 | 0.47981 |
| C | -4.26750 | 0.50225 | 0.89273 |
| C | -2.78265 | -0.22305 | -0.89626 |
| C | -5.34777 | 0.53260 | 0.02647 |
| H | -4.41994 | 0.80588 | 1.92092 |
| C | -3.90221 | -0.21438 | -1.75511 |
| C | -5.15929 | 0.15495 | -1.30746 |
| H | -6.32148 | 0.84751 | 0.38099 |
| H | -3.73950 | -0.49499 | -2.78921 |
| H | -5.99333 | 0.16180 | -2.00070 |
| O | -1.61170 | -0.57308 | -1.43126 |
| N | 0.90204 | 2.86224 | -2.29993 |
| H | -0.01415 | 2.44530 | -2.37037 |
| N | 0.56316 | -2.62745 | -2.44630 |
| H | -0.29854 | -2.09446 | -2.40609 |
| H | 0.98683 | 3.76719 | -2.73713 |
| H | 0.48758 | -3.52319 | -2.90416 |
| Cu | -0.05179 | 0.21047 | -0.81213 |

HL-NH2 Fe(II)

ΔG = -2461.86347

| | | | |
|---|---|---|---|
| C | -1.90037 | -0.20421 | 1.44799 |
| C | -1.13287 | -0.10932 | 3.74351 |
| C | 0.18967 | -0.09536 | 3.29397 |
| H | -1.34752 | -0.06192 | 4.80458 |
| C | 0.42035 | -0.13819 | 1.93040 |
| H | 1.01672 | -0.02932 | 3.98905 |
| C | 1.83853 | -0.03190 | 1.35927 |
| H | 2.51997 | -0.02269 | 2.20863 |
| C | 3.31119 | -2.01706 | 0.78415 |
| C | 3.61185 | -3.08225 | -0.07548 |
| H | 3.92140 | -1.81198 | 1.65327 |
| C | 1.71204 | -2.48652 | -1.41793 |
| C | 2.81911 | -3.32299 | -1.17607 |
| H | 4.46459 | -3.71973 | 0.12582 |
| H | 3.02863 | -4.14216 | -1.85234 |
| C | 2.04989 | 1.29074 | 0.60303 |
| C | 3.12451 | 3.39710 | 0.27051 |
| C | 1.33235 | 2.66761 | -1.15169 |
| C | 2.29651 | 3.63818 | -0.80359 |
| H | 3.86482 | 4.13511 | 0.55645 |
| H | 2.36407 | 4.55866 | -1.36996 |
| C | 2.21507 | -1.22917 | 0.49479 |
| C | 2.99824 | 2.20931 | 1.00855 |
| H | 3.62346 | 2.01960 | 1.87078 |
| N | -0.60292 | -0.22605 | 1.04811 |
| N | 1.43082 | -1.45654 | -0.58944 |
| N | 1.25904 | 1.50476 | -0.47319 |
| C | -2.16852 | -0.14353 | 2.82938 |
| H | -3.19075 | -0.09073 | 3.17379 |
| C | -2.98195 | -0.19835 | 0.44652 |
| C | -4.27722 | -0.60883 | 0.83115 |
| C | -2.80241 | 0.26743 | -0.89020 |
| C | -5.35966 | -0.53631 | -0.02577 |
| H | -4.43052 | -1.01678 | 1.82225 |
| C | -3.91995 | 0.36609 | -1.73687 |
| C | -5.17810 | -0.02741 | -1.31606 |
| H | -6.33535 | -0.87224 | 0.30193 |
| H | -3.75783 | 0.74699 | -2.73803 |
| H | -6.01929 | 0.04803 | -1.99557 |
| O | -1.61279 | 0.62618 | -1.39697 |

| | | | |
|---|---|---|---|
| N | 0.46270 | 2.83770 | -2.18735 |
| H | -0.39668 | 2.29796 | -2.18100 |
| N | 0.91417 | -2.66520 | -2.51126 |
| H | -0.00157 | -2.24266 | -2.53888 |
| H | 0.40973 | 3.74582 | -2.62062 |
| H | 1.02227 | -3.50471 | -3.05782 |
| Fe | -0.04962 | -0.10153 | -0.79546 |

HL-NH2 Fe(III)

ΔG = -2461.659596

| | | | |
|---|---|---|---|
| C | -1.88408 | 0.02877 | 1.44968 |
| C | -1.10223 | 0.06163 | 3.75009 |
| C | 0.21406 | 0.03564 | 3.29649 |
| H | -1.31783 | 0.08093 | 4.81088 |
| C | 0.44116 | 0.02409 | 1.93439 |
| H | 1.04897 | 0.02788 | 3.98349 |
| C | 1.86149 | 0.00066 | 1.38899 |
| H | 2.54162 | -0.02439 | 2.23637 |
| C | 3.11503 | -2.13794 | 0.81472 |
| C | 3.27582 | -3.23241 | -0.05442 |
| H | 3.76179 | -1.99606 | 1.66916 |
| C | 1.40609 | -2.45453 | -1.35792 |
| C | 2.43614 | -3.39548 | -1.12898 |
| H | 4.06200 | -3.95486 | 0.12764 |
| H | 2.53640 | -4.23632 | -1.80262 |
| C | 2.15962 | 1.25426 | 0.58052 |
| C | 3.38290 | 3.23293 | 0.06487 |
| C | 1.54662 | 2.51376 | -1.32146 |
| C | 2.58514 | 3.43165 | -1.03428 |
| H | 4.17472 | 3.93705 | 0.28957 |
| H | 2.72743 | 4.28148 | -1.68882 |
| C | 2.10946 | -1.24197 | 0.54608 |
| C | 3.17502 | 2.12049 | 0.90072 |
| H | 3.79291 | 1.94373 | 1.76980 |
| N | -0.57981 | 0.03264 | 1.03856 |
| N | 1.28221 | -1.39233 | -0.52222 |
| N | 1.36246 | 1.44432 | -0.50490 |
| C | -2.13376 | 0.05648 | 2.83535 |
| H | -3.14765 | 0.07229 | 3.19896 |
| C | -3.00622 | -0.01748 | 0.49233 |
| C | -4.33945 | -0.07484 | 0.94994 |
| C | -2.82411 | -0.02617 | -0.91756 |
| C | -5.41622 | -0.13014 | 0.08005 |
| H | -4.55552 | -0.08358 | 2.00724 |
| C | -3.91970 | -0.08289 | -1.79685 |
| C | -5.20864 | -0.13283 | -1.30430 |
| H | -6.42091 | -0.17420 | 0.48134 |
| H | -3.71435 | -0.08602 | -2.85984 |
| H | -6.04971 | -0.17661 | -1.98547 |
| O | -1.61695 | 0.01848 | -1.47921 |
| N | 0.75859 | 2.68076 | -2.40768 |
| H | -0.10432 | 2.17410 | -2.53809 |
| N | 0.55879 | -2.57642 | -2.40540 |
| H | -0.31697 | -2.07394 | -2.42853 |
| H | 0.84651 | 3.53275 | -2.93951 |
| H | 0.58932 | -3.42265 | -2.95282 |
| Fe | -0.00228 | 0.05633 | -0.77489 |

HL-NH2 K(I)

ΔG = -1798.254357

| | | | |
|---|---|---|---|
| C | -2.05662 | 0.24060 | 1.35134 |
| C | -1.15934 | 1.28215 | 3.33395 |
| C | 0.11591 | 1.03174 | 2.84263 |
| H | -1.29508 | 1.77641 | 4.28963 |
| C | 0.24545 | 0.39893 | 1.60267 |
| H | 0.99682 | 1.31234 | 3.40769 |
| C | 1.65681 | 0.09034 | 1.09247 |
| H | 2.26751 | -0.03013 | 1.99089 |
| C | 1.87809 | -2.41835 | 1.11407 |
| C | 1.96111 | -3.63717 | 0.43815 |
| H | 1.91517 | -2.37607 | 2.19536 |
| C | 1.81294 | -2.43246 | -1.62437 |
| C | 1.93097 | -3.65903 | -0.94502 |
| H | 2.06185 | -4.56355 | 0.99258 |
| H | 2.00646 | -4.58804 | -1.49703 |
| C | 2.29446 | 1.24728 | 0.32325 |
| C | 4.23404 | 2.51791 | -0.28156 |
| C | 2.04692 | 3.06774 | -1.08202 |
| C | 3.42664 | 3.34182 | -1.04568 |
| H | 5.30016 | 2.70703 | -0.22355 |
| H | 3.83423 | 4.18249 | -1.59399 |
| C | 1.74957 | -1.25139 | 0.36310 |
| C | 3.66863 | 1.45469 | 0.42574 |
| H | 4.27892 | 0.80705 | 1.04260 |
| N | -0.81147 | 0.02668 | 0.86892 |
| N | 1.71285 | -1.25925 | -0.97764 |
| N | 1.50223 | 2.03453 | -0.41932 |
| C | -2.25738 | 0.87770 | 2.58486 |
| H | -3.26518 | 1.05033 | 2.94105 |
| C | -3.23029 | -0.26399 | 0.58689 |
| C | -4.09596 | -1.16196 | 1.22495 |
| C | -3.51063 | 0.18957 | -0.75395 |
| C | -5.22603 | -1.68490 | 0.59667 |
| H | -3.86306 | -1.47356 | 2.23969 |

| | | | |
|---|---|---|---|
| C | -4.67814 | -0.37183 | -1.36455 |
| C | -5.50387 | -1.27694 | -0.71400 |
| H | -5.86664 | -2.39206 | 1.11085 |
| H | -4.91093 | -0.05030 | -2.37586 |
| H | -6.37693 | -1.66862 | -1.22899 |
| O | -2.78198 | 1.06233 | -1.36649 |
| N | 1.18572 | 3.82778 | -1.85234 |
| H | 0.21814 | 3.81266 | -1.56120 |
| N | 1.74309 | -2.38400 | -3.00461 |
| H | 2.01383 | -1.50464 | -3.42197 |
| H | 1.50964 | 4.74516 | -2.12252 |
| H | 2.08803 | -3.19008 | -3.50512 |
| K | -0.37429 | 0.48894 | -2.00265 |

HL-NH2 Li(I)

ΔG = -1205.868101

| | | | |
|---|---|---|---|
| C | 1.87933 | -0.03314 | 1.36257 |
| C | 1.09064 | -0.37765 | 3.62301 |
| C | -0.22230 | -0.25463 | 3.16631 |
| H | 1.28737 | -0.56607 | 4.67259 |
| C | -0.41910 | -0.04381 | 1.80429 |
| H | -1.06302 | -0.34173 | 3.84317 |
| C | -1.83633 | -0.00622 | 1.21935 |
| H | -2.52859 | -0.03677 | 2.06018 |
| C | -3.13401 | 2.13467 | 0.91211 |
| C | -3.39484 | 3.30284 | 0.18817 |
| H | -3.68953 | 1.89883 | 1.81034 |
| C | -1.67555 | 2.66187 | -1.35308 |
| C | -2.66902 | 3.57893 | -0.95364 |
| H | -4.16264 | 3.99149 | 0.52236 |
| H | -2.84779 | 4.47538 | -1.53464 |
| C | -2.09937 | -1.25991 | 0.37951 |
| C | -3.26786 | -3.29018 | -0.09473 |
| C | -1.38570 | -2.57810 | -1.39676 |
| C | -2.40055 | -3.52470 | -1.14636 |
| H | -4.05499 | -4.00356 | 0.12287 |
| H | -2.48496 | -4.41351 | -1.75962 |
| C | -2.13916 | 1.28004 | 0.45005 |
| C | -3.12216 | -2.14538 | 0.69969 |
| H | -3.77845 | -1.95876 | 1.53975 |
| N | 0.60102 | 0.08213 | 0.94716 |
| N | -1.42481 | 1.53822 | -0.66173 |
| N | -1.27287 | -1.46652 | -0.65456 |
| C | 2.14273 | -0.28690 | 2.72539 |
| H | 3.15725 | -0.43261 | 3.07002 |
| C | 2.97440 | 0.09751 | 0.37303 |
| C | 4.23059 | 0.55110 | 0.82448 |
| C | 2.81739 | -0.24770 | -1.02597 |
| C | 5.33946 | 0.65788 | -0.00289 |
| H | 4.33254 | 0.85409 | 1.86089 |
| C | 3.99219 | -0.15550 | -1.83524 |
| C | 5.20922 | 0.28492 | -1.34847 |
| H | 6.28130 | 1.02775 | 0.38509 |
| H | 3.88642 | -0.43689 | -2.87859 |
| H | 6.06343 | 0.34953 | -2.01606 |
| O | 1.70264 | -0.63822 | -1.56404 |
| N | -0.47969 | -2.71289 | -2.42324 |
| H | 0.38005 | -2.17848 | -2.31582 |
| N | -0.95285 | 2.86594 | -2.50848 |
| H | -0.07984 | 2.36513 | -2.58894 |
| H | -0.36623 | -3.63361 | -2.82014 |
| H | -0.92072 | 3.80804 | -2.86875 |
| Li | 0.07061 | 0.03797 | -0.97042 |

HL-NH2 Mg(II)

ΔG = -1398.337895

| | | | |
|---|---|---|---|
| C | -1.92918 | 0.13708 | 1.37531 |
| C | -1.13157 | 0.43705 | 3.63900 |
| C | 0.17793 | 0.26223 | 3.19066 |
| H | -1.32659 | 0.63144 | 4.68722 |
| C | 0.37706 | 0.04516 | 1.83452 |
| H | 1.01720 | 0.31547 | 3.87163 |
| C | 1.79334 | -0.05635 | 1.25854 |
| H | 2.47094 | -0.03819 | 2.10940 |
| C | 3.03878 | -2.22086 | 1.04374 |
| C | 3.31343 | -3.41476 | 0.36400 |
| H | 3.56463 | -1.96395 | 1.95302 |
| C | 1.65894 | -2.80414 | -1.26895 |
| C | 2.63086 | -3.71164 | -0.79144 |
| H | 4.05809 | -4.10196 | 0.74809 |
| H | 2.81802 | -4.62644 | -1.33919 |
| C | 2.13453 | 1.17685 | 0.41626 |
| C | 3.43155 | 3.13123 | -0.03563 |
| C | 1.52023 | 2.55886 | -1.36854 |
| C | 2.59682 | 3.42777 | -1.09190 |
| H | 4.26045 | 3.78946 | 0.19741 |
| H | 2.74292 | 4.31493 | -1.69474 |
| C | 2.07867 | -1.37072 | 0.52811 |
| C | 3.19609 | 1.99580 | 0.75373 |
| H | 3.82132 | 1.76723 | 1.60601 |
| N | -0.64846 | -0.03242 | 0.96842 |
| N | 1.39215 | -1.65157 | -0.61333 |
| N | 1.33648 | 1.43572 | -0.64417 |
| C | -2.18114 | 0.39172 | 2.73676 |

| | | | |
|---|---|---|---|
| H | -3.18959 | 0.57793 | 3.07685 |
| C | -3.03344 | 0.07244 | 0.38933 |
| C | -4.31245 | -0.31271 | 0.84388 |
| C | -2.87789 | 0.42133 | -0.99606 |
| C | -5.42512 | -0.33438 | 0.01771 |
| H | -4.42978 | -0.62630 | 1.87440 |
| C | -4.03997 | 0.42290 | -1.80699 |
| C | -5.28168 | 0.05332 | -1.32024 |
| H | -6.38646 | -0.65053 | 0.40438 |
| H | -3.91505 | 0.71189 | -2.84502 |
| H | -6.14073 | 0.05374 | -1.98326 |
| O | -1.72450 | 0.75260 | -1.54565 |
| N | 0.64302 | 2.79692 | -2.38998 |
| H | -0.27884 | 2.37702 | -2.32771 |
| N | 1.01366 | -3.07910 | -2.44134 |
| H | 0.13809 | -2.63847 | -2.67475 |
| H | 0.66601 | 3.70844 | -2.82116 |
| H | 1.11380 | -4.01173 | -2.81167 |
| Mg | -0.06379 | -0.11267 | -1.05433 |

HL-NH2 Mn(II)

ΔG = -2349.155382

| | | | |
|---|---|---|---|
| C | 1.90747 | 0.22124 | 1.43480 |
| C | 1.13943 | 0.06710 | 3.72428 |
| C | -0.17591 | 0.03409 | 3.26682 |
| H | 1.35157 | 0.00314 | 4.78474 |
| C | -0.40396 | 0.10509 | 1.90036 |
| H | -1.00678 | -0.06281 | 3.95298 |
| C | -1.82160 | -0.02371 | 1.34564 |
| H | -2.48984 | -0.05704 | 2.20370 |
| C | -3.39422 | 1.87303 | 0.83847 |
| C | -3.78076 | 2.94587 | 0.02877 |
| H | -3.97210 | 1.58943 | 1.70747 |
| C | -1.85319 | 2.53375 | -1.35198 |
| C | -3.01187 | 3.28074 | -1.06453 |
| H | -4.67179 | 3.51599 | 0.26295 |
| H | -3.27537 | 4.11359 | -1.70406 |
| C | -2.00976 | -1.34458 | 0.58992 |
| C | -3.03445 | -3.47621 | 0.26806 |
| C | -1.28437 | -2.69340 | -1.17717 |
| C | -2.21530 | -3.69229 | -0.81990 |
| H | -3.75050 | -4.23486 | 0.56195 |
| H | -2.26551 | -4.61125 | -1.39020 |
| C | -2.25073 | 1.17068 | 0.50428 |
| C | -2.93122 | -2.28750 | 1.00649 |
| H | -3.54966 | -2.11148 | 1.87620 |
| N | 0.61360 | 0.23188 | 1.01860 |
| N | -1.49200 | 1.47766 | -0.58107 |
| N | -1.22457 | -1.53503 | -0.49051 |
| C | 2.17758 | 0.14018 | 2.81059 |
| H | 3.20014 | 0.09989 | 3.15640 |
| C | 3.00486 | 0.24397 | 0.44281 |
| C | 4.26928 | 0.72940 | 0.83454 |
| C | 2.86089 | -0.27760 | -0.88044 |
| C | 5.37563 | 0.67753 | 0.00140 |
| H | 4.38077 | 1.17368 | 1.81606 |
| C | 4.00894 | -0.35434 | -1.69857 |
| C | 5.24135 | 0.11162 | -1.27154 |
| H | 6.32773 | 1.07175 | 0.33518 |
| H | 3.88642 | -0.77647 | -2.68965 |
| H | 6.09763 | 0.04975 | -1.93453 |
| O | 1.70530 | -0.69709 | -1.38933 |
| N | -0.43724 | -2.83583 | -2.24043 |
| H | 0.41044 | -2.27785 | -2.23122 |
| N | -1.10458 | 2.84051 | -2.46061 |
| H | -0.11578 | 2.63975 | -2.47027 |
| H | -0.33491 | -3.76085 | -2.62979 |
| H | -1.32808 | 3.71391 | -2.91479 |
| Mn | 0.08273 | 0.17345 | -0.90796 |

HL-NH2 Na(I)

ΔG = -1360.620042

| | | | |
|---|---|---|---|
| C | -1.95242 | 0.10915 | 1.33816 |
| C | -1.09271 | 0.40412 | 3.57500 |
| C | 0.19462 | 0.24073 | 3.06920 |
| H | -1.24700 | 0.58921 | 4.63233 |
| C | 0.34799 | 0.02000 | 1.69863 |
| H | 1.05950 | 0.29777 | 3.71869 |
| C | 1.76883 | -0.07096 | 1.12527 |
| H | 2.43117 | -0.06299 | 1.99075 |
| C | 3.00197 | -2.25122 | 0.98725 |
| C | 3.30868 | -3.44600 | 0.33022 |
| H | 3.46591 | -2.00392 | 1.93312 |
| C | 1.78929 | -2.80128 | -1.40442 |
| C | 2.70525 | -3.73365 | -0.87735 |
| H | 4.01598 | -4.14318 | 0.76516 |
| H | 2.92119 | -4.64868 | -1.41529 |
| C | 2.13580 | 1.18509 | 0.32488 |
| C | 3.55748 | 3.06612 | -0.09075 |
| C | 1.50907 | 2.73452 | -1.28402 |
| C | 2.66304 | 3.50826 | -1.05015 |
| H | 4.45276 | 3.64161 | 0.11724 |
| H | 2.83162 | 4.42754 | -1.59803 |
| C | 2.08635 | -1.38096 | 0.39959 |

| | | | |
|---|---|---|---|
| C | 3.29555 | 1.89481 | 0.62876 |
| H | 3.96834 | 1.55482 | 1.40566 |
| N | -0.69920 | -0.06813 | 0.86916 |
| N | 1.48926 | -1.65202 | -0.77606 |
| N | 1.28825 | 1.58587 | -0.62926 |
| C | -2.17272 | 0.35607 | 2.70618 |
| H | -3.17771 | 0.52542 | 3.07048 |
| C | -3.10085 | 0.04092 | 0.39828 |
| C | -4.25772 | -0.63800 | 0.81902 |
| C | -3.08625 | 0.69596 | -0.89519 |
| C | -5.40826 | -0.72211 | 0.04178 |
| H | -4.24196 | -1.13536 | 1.78433 |
| C | -4.29885 | 0.60256 | -1.65165 |
| C | -5.41610 | -0.08322 | -1.20576 |
| H | -6.27306 | -1.27227 | 0.39408 |
| H | -4.31109 | 1.09949 | -2.61742 |
| H | -6.30305 | -0.12653 | -1.83176 |
| O | -2.06963 | 1.34568 | -1.36309 |
| N | 0.55430 | 3.08870 | -2.21638 |
| H | -0.37056 | 2.69973 | -2.05129 |
| N | 1.20465 | -3.02731 | -2.63419 |
| H | 0.35224 | -2.52579 | -2.83350 |
| H | 0.55285 | 4.05054 | -2.52237 |
| H | 1.19777 | -3.98104 | -2.96454 |
| Na | -0.27519 | -0.03519 | -1.48730 |

HL-NH2 Ni(II)

ΔG = -2706.439821

| | | | |
|---|---|---|---|
| C | 1.90759 | -0.17717 | 1.47784 |
| C | 1.13945 | -0.08162 | 3.76478 |
| C | -0.17843 | -0.09368 | 3.31042 |
| H | 1.34879 | -0.02308 | 4.82604 |
| C | -0.40968 | -0.16566 | 1.94424 |
| H | -1.01030 | -0.03318 | 3.99932 |
| C | -1.82627 | -0.11069 | 1.37589 |
| H | -2.51161 | -0.14779 | 2.22013 |
| C | -3.03663 | 2.10607 | 1.10886 |
| C | -3.25019 | 3.28424 | 0.37959 |
| H | -3.58158 | 1.90428 | 2.02123 |
| C | -1.57743 | 2.57110 | -1.18788 |
| C | -2.52504 | 3.52923 | -0.76810 |
| H | -3.97815 | 4.00977 | 0.72363 |
| H | -2.66258 | 4.43911 | -1.33890 |
| C | -2.11775 | -1.30331 | 0.48935 |
| C | -3.38859 | -3.21394 | -0.16492 |
| C | -1.46670 | -2.49622 | -1.42327 |
| C | -2.53982 | -3.39073 | -1.23521 |
| H | -4.21208 | -3.90069 | -0.00990 |
| H | -2.67352 | -4.20797 | -1.93223 |
| C | -2.10422 | 1.20387 | 0.62478 |
| C | -3.18110 | -2.15174 | 0.72463 |
| H | -3.83091 | -1.99104 | 1.57365 |
| N | 0.61753 | -0.25531 | 1.07288 |
| N | -1.41101 | 1.42291 | -0.50876 |
| N | -1.27956 | -1.46809 | -0.56139 |
| C | 2.18096 | -0.10113 | 2.85207 |
| H | 3.20425 | -0.02497 | 3.19121 |
| C | 2.96927 | -0.10791 | 0.46208 |
| C | 4.27253 | -0.54476 | 0.76095 |
| C | 2.71592 | 0.47684 | -0.81317 |
| C | 5.31561 | -0.40026 | -0.14097 |
| H | 4.46328 | -1.02155 | 1.71517 |
| C | 3.79249 | 0.63581 | -1.70839 |
| C | 5.06883 | 0.20745 | -1.37755 |
| H | 6.30658 | -0.75787 | 0.11020 |
| H | 3.58990 | 1.09797 | -2.66749 |
| H | 5.87563 | 0.33451 | -2.09111 |
| O | 1.51152 | 0.91443 | -1.18378 |
| N | -0.63400 | -2.62059 | -2.49613 |
| H | 0.28444 | -2.20293 | -2.47260 |
| N | -0.82077 | 2.74499 | -2.31741 |
| H | 0.05640 | 2.23834 | -2.34529 |
| H | -0.68421 | -3.48015 | -3.02176 |
| H | -0.77600 | 3.67915 | -2.69666 |
| Ni | 0.10105 | -0.14462 | -0.76410 |

HL-NH2 V(II)

ΔG = -2142.16889

| | | | |
|---|---|---|---|
| C | 1.86745 | -0.06257 | 1.45671 |
| C | 1.02165 | -0.28661 | 3.72738 |
| C | -0.28677 | -0.19934 | 3.24292 |
| H | 1.20870 | -0.41060 | 4.78645 |
| C | -0.47960 | -0.07204 | 1.88317 |
| H | -1.13660 | -0.24805 | 3.91261 |
| C | -1.87441 | -0.04517 | 1.25732 |
| H | -2.58805 | -0.08557 | 2.07829 |
| C | -3.20941 | 2.08229 | 0.91334 |
| C | -3.43284 | 3.28256 | 0.22320 |
| H | -3.83978 | 1.79444 | 1.74412 |
| C | -1.56120 | 2.77605 | -1.19134 |
| C | -2.61182 | 3.63729 | -0.82544 |
| H | -4.24749 | 3.93401 | 0.51613 |
| H | -2.75998 | 4.56202 | -1.36873 |
| C | -2.12175 | -1.31178 | 0.42140 |

| | | | |
|---|---|---|---|
| C | -3.29385 | -3.34113 | -0.06186 |
| C | -1.36378 | -2.69362 | -1.33578 |
| C | -2.39824 | -3.60941 | -1.07785 |
| H | -4.09854 | -4.03653 | 0.14470 |
| H | -2.47820 | -4.50955 | -1.67417 |
| C | -2.16627 | 1.27160 | 0.51693 |
| C | -3.15516 | -2.17930 | 0.71126 |
| H | -3.83718 | -1.96768 | 1.52408 |
| N | 0.56876 | -0.00207 | 1.00664 |
| N | -1.35997 | 1.60815 | -0.53517 |
| N | -1.25931 | -1.55337 | -0.60923 |
| C | 2.08009 | -0.22909 | 2.83321 |
| H | 3.08629 | -0.32843 | 3.21122 |
| C | 2.98366 | 0.03976 | 0.49141 |
| C | 4.29714 | 0.23805 | 0.97758 |
| C | 2.84268 | -0.04682 | -0.92437 |
| C | 5.40181 | 0.31085 | 0.14907 |
| H | 4.45692 | 0.35959 | 2.03983 |
| C | 3.96975 | 0.01952 | -1.75588 |
| C | 5.23930 | 0.19072 | -1.23351 |
| H | 6.38459 | 0.46816 | 0.57593 |
| H | 3.80808 | -0.06393 | -2.82389 |
| H | 6.09565 | 0.24260 | -1.89549 |
| O | 1.64834 | -0.19559 | -1.55767 |
| N | -0.45364 | -2.88019 | -2.33397 |
| H | 0.41654 | -2.36547 | -2.31942 |
| N | -0.73344 | 3.06729 | -2.23475 |
| H | 0.13674 | 2.57194 | -2.35183 |
| H | -0.43382 | -3.76637 | -2.81266 |
| H | -0.78671 | 3.97579 | -2.66613 |
| V | -0.01745 | 0.04908 | -0.85250 |

HL-NH2 V(III)

ΔG = -2141.996018

| | | | |
|---|---|---|---|
| C | 1.90512 | 0.30241 | 1.42791 |
| C | 1.11494 | 0.52302 | 3.70391 |
| C | -0.19609 | 0.43470 | 3.24223 |
| H | 1.31764 | 0.63657 | 4.76144 |
| C | -0.41111 | 0.26031 | 1.88608 |
| H | -1.03685 | 0.48525 | 3.92097 |
| C | -1.82332 | 0.12101 | 1.32785 |
| H | -2.50456 | 0.18697 | 2.17245 |
| C | -3.24966 | 2.09062 | 0.67565 |
| C | -3.56427 | 3.12458 | -0.22632 |
| H | -3.82928 | 1.93940 | 1.57541 |
| C | -1.71944 | 2.43436 | -1.61026 |
| C | -2.81213 | 3.29963 | -1.35791 |
| H | -4.39991 | 3.78350 | -0.02402 |
| H | -3.02843 | 4.09029 | -2.06416 |
| C | -2.03999 | -1.25089 | 0.69631 |
| C | -3.16994 | -3.34478 | 0.49871 |
| C | -1.32285 | -2.79464 | -0.93884 |
| C | -2.32804 | -3.69754 | -0.52847 |
| H | -3.94058 | -4.03274 | 0.82470 |
| H | -2.40899 | -4.65724 | -1.02189 |
| C | -2.19223 | 1.26618 | 0.38597 |
| C | -3.02585 | -2.10046 | 1.13792 |
| H | -3.67201 | -1.81229 | 1.95524 |
| N | 0.61320 | 0.20817 | 1.00128 |
| N | -1.43407 | 1.42082 | -0.74668 |
| N | -1.21645 | -1.57678 | -0.34168 |
| C | 2.15905 | 0.44347 | 2.79986 |
| H | 3.17760 | 0.47125 | 3.15651 |
| C | 3.01546 | 0.21813 | 0.45687 |
| C | 4.30658 | 0.66568 | 0.79360 |
| C | 2.85250 | -0.34071 | -0.83018 |
| C | 5.37283 | 0.53856 | -0.08504 |
| H | 4.47739 | 1.14325 | 1.74883 |
| C | 3.92268 | -0.48769 | -1.71347 |
| C | 5.18480 | -0.05033 | -1.33843 |
| H | 6.35021 | 0.90425 | 0.20434 |
| H | 3.74001 | -0.93434 | -2.68266 |
| H | 6.01664 | -0.15501 | -2.02444 |
| O | 1.62068 | -0.74836 | -1.23343 |
| N | -0.45973 | -3.10038 | -1.93659 |
| H | 0.42178 | -2.61223 | -2.01333 |
| N | -0.96950 | 2.60970 | -2.71629 |
| H | -0.12318 | 2.09858 | -2.90405 |
| H | -0.47585 | -4.03488 | -2.31520 |
| H | -1.17326 | 3.37949 | -3.33358 |
| V | 0.02252 | -0.07642 | -0.92666 |

HL-NH2 Zn(II)

ΔG = -2977.513327

| | | | |
|---|---|---|---|
| C | -1.88443 | 0.09970 | 1.48198 |
| C | -1.10221 | 0.52669 | 3.73135 |
| C | 0.21751 | 0.40048 | 3.29390 |
| H | -1.30949 | 0.74903 | 4.77155 |
| C | 0.42913 | 0.14858 | 1.94671 |
| H | 1.05035 | 0.51872 | 3.97446 |
| C | 1.83992 | 0.09931 | 1.34856 |
| H | 2.53891 | 0.17352 | 2.17871 |
| C | 3.17049 | -2.02403 | 1.12141 |
| C | 3.45722 | -3.21874 | 0.44533 |

| | | | |
|---|---|---|---|
| H | 3.72713 | -1.73302 | 2.00142 |
| C | 1.71011 | -2.70340 | -1.12279 |
| C | 2.73677 | -3.56393 | -0.67335 |
| H | 4.24500 | -3.87026 | 0.80447 |
| H | 2.93695 | -4.47982 | -1.21433 |
| C | 2.08629 | 1.32111 | 0.45683 |
| C | 3.19592 | 3.36420 | -0.08695 |
| C | 1.35794 | 2.55354 | -1.40122 |
| C | 2.34570 | 3.53170 | -1.15901 |
| H | 3.95661 | 4.10864 | 0.11707 |
| H | 2.41268 | 4.39940 | -1.80260 |
| C | 2.15828 | -1.21839 | 0.63892 |
| C | 3.06679 | 2.24827 | 0.75469 |
| H | 3.70815 | 2.11810 | 1.61567 |
| N | -0.59535 | -0.01039 | 1.09259 |
| N | 1.43712 | -1.55384 | -0.46590 |
| N | 1.27436 | 1.46235 | -0.61452 |
| C | -2.14898 | 0.39556 | 2.83331 |
| H | -3.16408 | 0.54360 | 3.17147 |
| C | -2.97331 | -0.08091 | 0.49159 |
| C | -4.23912 | -0.47665 | 0.97784 |
| C | -2.83604 | 0.14791 | -0.92048 |
| C | -5.35116 | -0.61015 | 0.16326 |
| H | -4.34514 | -0.70860 | 2.03039 |
| C | -3.99718 | 0.03565 | -1.72241 |
| C | -5.22512 | -0.33311 | -1.20299 |
| H | -6.29843 | -0.92874 | 0.58118 |
| H | -3.88089 | 0.23582 | -2.78181 |
| H | -6.08326 | -0.42078 | -1.86099 |
| O | -1.70947 | 0.48320 | -1.53861 |
| N | 0.47812 | 2.64796 | -2.43985 |
| H | -0.38932 | 2.12509 | -2.37445 |
| N | 1.01319 | -3.00820 | -2.25172 |
| H | 0.13607 | -2.55737 | -2.46039 |
| H | 0.41101 | 3.53416 | -2.91667 |
| H | 1.11601 | -3.93525 | -2.63390 |
| Zn | -0.01439 | -0.14544 | -0.88385 |

EDTA-4H

ΔG = -1100.357742

| | | | |
|---|---|---|---|
| C | 1.766618000 | -3.208435000 | 1.143156000 |
| O | 1.847097000 | -3.699685000 | 2.306332000 |
| O | 2.574973000 | -3.404929000 | 0.201722000 |
| C | 0.558290000 | -2.253827000 | 0.939874000 |
| H | -0.303746000 | -2.694183000 | 1.461002000 |
| H | 0.815120000 | -1.344319000 | 1.492745000 |
| N | 0.225135000 | -1.878071000 | -0.429515000 |
| C | -0.483361000 | -0.598246000 | -0.529778000 |
| H | -1.043423000 | -0.585180000 | -1.469205000 |
| H | -1.229406000 | -0.481005000 | 0.269696000 |
| C | -0.350752000 | -2.964383000 | -1.212608000 |
| H | -0.391517000 | -2.653927000 | -2.263118000 |
| H | 0.341518000 | -3.810361000 | -1.178382000 |
| C | -1.766618000 | -3.520233000 | -0.865892000 |
| O | -2.128012000 | -4.502230000 | -1.576867000 |
| O | -2.436306000 | -2.979155000 | 0.049482000 |
| C | -1.766618000 | 3.208435000 | 1.143156000 |
| O | -1.847097000 | 3.699685000 | 2.306332000 |
| O | -2.574973000 | 3.404929000 | 0.201722000 |
| C | -0.558290000 | 2.253827000 | 0.939874000 |
| H | 0.303746000 | 2.694183000 | 1.461002000 |
| H | -0.815120000 | 1.344319000 | 1.492745000 |
| N | -0.225135000 | 1.878071000 | -0.429515000 |
| C | 0.483361000 | 0.598246000 | -0.529778000 |
| H | 1.043423000 | 0.585180000 | -1.469205000 |
| H | 1.229406000 | 0.481005000 | 0.269696000 |
| C | 0.350752000 | 2.964383000 | -1.212608000 |
| H | 0.391517000 | 2.653927000 | -2.263118000 |
| H | -0.341518000 | 3.810361000 | -1.178382000 |
| C | 1.766618000 | 3.520233000 | -0.865892000 |
| O | 2.128012000 | 4.502230000 | -1.576867000 |
| O | 2.436306000 | 2.979155000 | 0.049482000 |

EDTA Al(III)

ΔG = -1342.623362

| | | | |
|---|---|---|---|
| C | -1.452641000 | 2.267944000 | 0.800801000 |
| O | -0.676602000 | 1.510414000 | 1.525453000 |
| O | -2.240399000 | 3.094593000 | 1.239352000 |
| C | -1.305796000 | 2.074756000 | -0.733249000 |
| H | -2.119236000 | 1.436563000 | -1.081261000 |
| H | -1.380391000 | 3.039065000 | -1.240968000 |
| N | 0.000000000 | 1.412269000 | -0.965332000 |
| C | 0.249019000 | 0.722682000 | -2.264541000 |
| H | -0.199540000 | 1.269030000 | -3.098677000 |
| H | 1.328426000 | 0.732984000 | -2.418005000 |
| C | 1.110584000 | 2.367071000 | -0.689960000 |
| H | 1.505075000 | 2.798159000 | -1.611389000 |
| H | 0.747280000 | 3.193329000 | -0.077422000 |
| C | 2.213125000 | 1.669071000 | 0.104356000 |
| O | 3.330433000 | 2.167319000 | 0.191157000 |
| O | 1.834894000 | 0.566809000 | 0.666296000 |
| C | 1.452641000 | -2.267944000 | 0.800801000 |
| O | 0.676602000 | -1.510414000 | 1.525453000 |

| | | | |
|---|---|---|---|
| O | 2.240399000 | -3.094593000 | 1.239352000 |
| C | 1.305796000 | -2.074756000 | -0.733249000 |
| H | 2.119236000 | -1.436563000 | -1.081261000 |
| H | 1.380391000 | -3.039065000 | -1.240968000 |
| N | 0.000000000 | -1.412269000 | -0.965332000 |
| C | -0.249019000 | -0.722682000 | -2.264541000 |
| H | 0.199540000 | -1.269030000 | -3.098677000 |
| H | -1.328426000 | -0.732984000 | -2.418005000 |
| C | -1.110584000 | -2.367071000 | -0.689960000 |
| H | -1.505075000 | -2.798159000 | -1.611389000 |
| H | -0.747280000 | -3.193329000 | -0.077422000 |
| C | -2.213125000 | -1.669071000 | 0.104356000 |
| O | -3.330433000 | -2.167319000 | 0.191157000 |
| O | -1.834894000 | -0.566809000 | 0.666296000 |
| Al | 0.000000000 | 0.000000000 | 0.615776000 |

EDTA Be(II)

ΔG = -1115.033923

| | | | |
|---|---|---|---|
| C | -0.888592000 | -2.024787000 | 1.223534000 |
| O | -1.241350000 | -0.905024000 | 1.774078000 |
| O | -0.805149000 | -3.106009000 | 1.802771000 |
| C | -0.515180000 | -1.888953000 | -0.254367000 |
| H | 0.576896000 | -1.820769000 | -0.280680000 |
| H | -0.833892000 | -2.765147000 | -0.825304000 |
| N | -1.107212000 | -0.628860000 | -0.769761000 |
| C | -0.416235000 | -0.076895000 | -1.973898000 |
| H | -0.613649000 | -0.725667000 | -2.835589000 |
| H | -0.894391000 | 0.881855000 | -2.189796000 |
| C | -2.558881000 | -0.796247000 | -1.084561000 |
| H | -2.734415000 | -0.863560000 | -2.159639000 |
| H | -2.925445000 | -1.723882000 | -0.638489000 |
| C | -3.378418000 | 0.345395000 | -0.473441000 |
| O | -4.525093000 | 0.568344000 | -0.854739000 |
| O | -2.755839000 | 0.976729000 | 0.466853000 |
| C | 0.510671000 | 2.409541000 | 0.908343000 |
| O | -0.356501000 | 1.563263000 | 1.357200000 |
| O | 0.853528000 | 3.421652000 | 1.523756000 |
| C | 1.100114000 | 2.111169000 | -0.476287000 |
| H | 0.310418000 | 2.337543000 | -1.199268000 |
| H | 1.913637000 | 2.819299000 | -0.672863000 |
| N | 1.529401000 | 0.719515000 | -0.616002000 |
| C | 1.100295000 | 0.084072000 | -1.854930000 |
| H | 1.430791000 | 0.637728000 | -2.753743000 |
| H | 1.575001000 | -0.895705000 | -1.899331000 |
| C | 2.962800000 | 0.575146000 | -0.359995000 |
| H | 3.565088000 | 0.998209000 | -1.182703000 |
| H | 3.207195000 | 1.165665000 | 0.528433000 |
| C | 3.504388000 | -0.851847000 | -0.075531000 |
| O | 4.762152000 | -0.923813000 | -0.039338000 |
| O | 2.694145000 | -1.792873000 | 0.114360000 |
| Be | -1.266367000 | 0.395529000 | 0.794144000 |

EDTA B(III)

ΔG = -1124.913382

| | | | |
|---|---|---|---|
| C | -0.694813000 | -2.204090000 | 0.892591000 |
| O | -1.375338000 | -1.227260000 | 1.499642000 |
| O | -0.498086000 | -3.289519000 | 1.380914000 |
| C | -0.185614000 | -1.749455000 | -0.465309000 |
| H | 0.884159000 | -1.524172000 | -0.358991000 |
| H | -0.320110000 | -2.512886000 | -1.229683000 |
| N | -0.961307000 | -0.504707000 | -0.770705000 |
| C | -0.255006000 | 0.510966000 | -1.652127000 |
| H | -0.409470000 | 0.201142000 | -2.686475000 |
| H | -0.794280000 | 1.451840000 | -1.523718000 |
| C | -2.313374000 | -0.808383000 | -1.365675000 |
| H | -2.343308000 | -0.588619000 | -2.430450000 |
| H | -2.552890000 | -1.863665000 | -1.227704000 |
| C | -3.327992000 | 0.023294000 | -0.593319000 |
| O | -4.470851000 | 0.205352000 | -0.930556000 |
| O | -2.781299000 | 0.508424000 | 0.518587000 |
| C | -0.190431000 | 2.200723000 | 0.903204000 |
| O | -0.557367000 | 0.981313000 | 1.347086000 |
| O | -0.895058000 | 3.176466000 | 0.995841000 |
| C | 1.241651000 | 2.249209000 | 0.385029000 |
| H | 1.308092000 | 3.080551000 | -0.334064000 |
| H | 1.846328000 | 2.536339000 | 1.251344000 |
| N | 1.728349000 | 0.980021000 | -0.127471000 |
| C | 1.253429000 | 0.680918000 | -1.473791000 |
| H | 1.530215000 | 1.475929000 | -2.191319000 |
| H | 1.754268000 | -0.227654000 | -1.804521000 |
| C | 3.184314000 | 0.844030000 | -0.030211000 |
| H | 3.708237000 | 1.452502000 | -0.785902000 |
| H | 3.496776000 | 1.225783000 | 0.945096000 |
| C | 3.743383000 | -0.602313000 | -0.124854000 |
| O | 4.998845000 | -0.668652000 | -0.142925000 |
| O | 2.936754000 | -1.567051000 | -0.158344000 |
| B | -1.414281000 | 0.028746000 | 0.745689000 |

EDTA Ca(II)

ΔG = -1777.944127

| | | | |
|---|---|---|---|
| C | -0.195087000 | 3.138674000 | 0.446157000 |
| O | -0.043987000 | 2.382880000 | 1.456799000 |
| O | -0.516273000 | 4.343572000 | 0.465770000 |
| C | 0.000000000 | 2.488892000 | -0.946404000 |
| H | -0.999891000 | 2.239308000 | -1.311523000 |
| H | 0.414299000 | 3.235143000 | -1.638413000 |

| | | | |
|---|---|---|---|
| N | 0.817298000 | 1.268386000 | -0.892491000 |
| C | 0.654475000 | 0.394140000 | -2.071641000 |
| H | 0.717788000 | 0.970287000 | -3.007947000 |
| H | 1.498759000 | -0.299156000 | -2.081531000 |
| C | 2.238216000 | 1.596917000 | -0.683778000 |
| H | 2.753332000 | 1.792569000 | -1.633184000 |
| H | 2.302521000 | 2.516894000 | -0.095949000 |
| C | 3.020595000 | 0.535714000 | 0.119891000 |
| O | 4.257950000 | 0.504388000 | -0.033570000 |
| O | 2.343350000 | -0.187557000 | 0.915719000 |
| C | 0.195087000 | -3.138674000 | 0.446157000 |
| O | 0.043987000 | -2.382880000 | 1.456799000 |
| O | 0.516273000 | -4.343572000 | 0.465770000 |
| C | 0.000000000 | -2.488892000 | -0.946404000 |
| H | 0.999891000 | -2.239308000 | -1.311523000 |
| H | -0.414299000 | -3.235143000 | -1.638413000 |
| N | -0.817298000 | -1.268386000 | -0.892491000 |
| C | -0.654475000 | -0.394140000 | -2.071641000 |
| H | -0.717788000 | -0.970287000 | -3.007947000 |
| H | -1.498759000 | 0.299156000 | -2.081531000 |
| C | -2.238216000 | -1.596917000 | -0.683778000 |
| H | -2.753332000 | -1.792569000 | -1.633184000 |
| H | -2.302521000 | -2.516894000 | -0.095949000 |
| C | -3.020595000 | -0.535714000 | 0.119891000 |
| O | -4.257950000 | -0.504388000 | -0.033570000 |
| O | -2.343350000 | 0.187557000 | 0.915719000 |
| Ca | 0.000000000 | 0.000000000 | 1.239290000 |

EDTA Co(II)

ΔG = -2482.948385

| | | | |
|---|---|---|---|
| C | -1.481875000 | 2.285615000 | 0.846467000 |
| O | -0.716975000 | 1.584573000 | 1.603157000 |
| O | -2.324376000 | 3.108983000 | 1.230673000 |
| C | -1.315938000 | 2.064600000 | -0.684249000 |
| H | -2.109647000 | 1.393207000 | -1.013228000 |
| H | -1.412526000 | 3.016492000 | -1.217088000 |
| N | 0.000000000 | 1.426078000 | -0.910174000 |
| C | 0.252817000 | 0.720979000 | -2.204046000 |
| H | -0.184691000 | 1.268118000 | -3.046361000 |
| H | 1.334041000 | 0.724617000 | -2.346868000 |
| C | 1.089982000 | 2.413275000 | -0.675356000 |
| H | 1.333763000 | 2.939144000 | -1.603167000 |
| H | 0.744522000 | 3.160927000 | 0.039637000 |
| C | 2.349685000 | 1.768228000 | -0.069648000 |
| O | 3.413894000 | 2.416083000 | -0.141931000 |
| O | 2.175451000 | 0.644204000 | 0.485740000 |
| C | 1.481875000 | -2.285615000 | 0.846467000 |
| O | 0.716975000 | -1.584573000 | 1.603157000 |
| O | 2.324376000 | -3.108983000 | 1.230673000 |
| C | 1.315938000 | -2.064600000 | -0.684249000 |
| H | 2.109647000 | -1.393207000 | -1.013228000 |
| H | 1.412526000 | -3.016492000 | -1.217088000 |
| N | 0.000000000 | -1.426078000 | -0.910174000 |
| C | -0.252817000 | -0.720979000 | -2.204046000 |
| H | 0.184691000 | -1.268118000 | -3.046361000 |
| H | -1.334041000 | -0.724617000 | -2.346868000 |
| C | -1.089982000 | -2.413275000 | -0.675356000 |
| H | -1.333763000 | -2.939144000 | -1.603167000 |
| H | -0.744522000 | -3.160927000 | 0.039637000 |
| C | -2.349685000 | -1.768228000 | -0.069648000 |
| O | -3.413894000 | -2.416083000 | -0.141931000 |
| O | -2.175451000 | -0.644204000 | 0.485740000 |
| Co | 0.000000000 | 0.000000000 | 0.508012000 |

EDTA Co(III)

ΔG = -2482.822977

| | | | |
|---|---|---|---|
| C | -0.803201000 | 2.503143000 | 1.083008000 |
| O | -0.210773000 | 1.489871000 | 1.635941000 |
| O | -1.221410000 | 3.487067000 | 1.685709000 |
| C | -0.975771000 | 2.402938000 | -0.447768000 |
| H | -1.987552000 | 2.055838000 | -0.651604000 |
| H | -0.831555000 | 3.375121000 | -0.921737000 |
| N | 0.000000000 | 1.396868000 | -0.949692000 |
| C | -0.355795000 | 0.678591000 | -2.215388000 |
| H | -1.437699000 | 0.552642000 | -2.237141000 |
| H | -0.071293000 | 1.266665000 | -3.090996000 |
| C | 1.377988000 | 1.989671000 | -1.010540000 |
| H | 1.690497000 | 2.120602000 | -2.047520000 |
| H | 1.363145000 | 2.979019000 | -0.552788000 |
| C | 2.402471000 | 1.148344000 | -0.254830000 |
| O | 3.595350000 | 1.429185000 | -0.311687000 |
| O | 1.903263000 | 0.169021000 | 0.425184000 |
| C | 0.803201000 | -2.503143000 | 1.083008000 |
| O | 0.210773000 | -1.489871000 | 1.635941000 |
| O | 1.221410000 | -3.487067000 | 1.685709000 |
| C | 0.975771000 | -2.402938000 | -0.447768000 |
| H | 1.987552000 | -2.055838000 | -0.651604000 |
| H | 0.831555000 | -3.375121000 | -0.921737000 |
| N | 0.000000000 | -1.396868000 | -0.949692000 |
| C | 0.355795000 | -0.678591000 | -2.215388000 |
| H | 1.437699000 | -0.552642000 | -2.237141000 |
| H | 0.071293000 | -1.266665000 | -3.090996000 |
| C | -1.377988000 | -1.989671000 | -1.010540000 |
| H | -1.690497000 | -2.120602000 | -2.047520000 |
| H | -1.363145000 | -2.979019000 | -0.552788000 |
| C | -2.402471000 | -1.148344000 | -0.254830000 |

| | | | |
|---|---|---|---|
| O | -3.595350000 | -1.429185000 | -0.311687000 |
| O | -1.903263000 | -0.169021000 | 0.425184000 |
| Co | 0.000000000 | 0.000000000 | 0.425301000 |

EDTA Cr(II)

ΔG = -2144.673999

| | | | |
|---|---|---|---|
| C | -0.296469000 | 2.883306000 | 0.665401000 |
| O | 0.000000000 | 1.985912000 | 1.523298000 |
| O | -0.668853000 | 4.044133000 | 0.913682000 |
| C | -0.190248000 | 2.464951000 | -0.826800000 |
| H | -1.193505000 | 2.204275000 | -1.171926000 |
| H | 0.165679000 | 3.310349000 | -1.426955000 |
| N | 0.688935000 | 1.285127000 | -0.939342000 |
| C | 0.590856000 | 0.484400000 | -2.192025000 |
| H | 0.532132000 | 1.129638000 | -3.078196000 |
| H | 1.520941000 | -0.081245000 | -2.274686000 |
| C | 2.101879000 | 1.650942000 | -0.680771000 |
| H | 2.637435000 | 1.882067000 | -1.607229000 |
| H | 2.132984000 | 2.547567000 | -0.059198000 |
| C | 2.828675000 | 0.548074000 | 0.101101000 |
| O | 4.069416000 | 0.550524000 | 0.126206000 |
| O | 2.067131000 | -0.283081000 | 0.704833000 |
| C | 0.296469000 | -2.883306000 | 0.665401000 |
| O | 0.000000000 | -1.985912000 | 1.523298000 |
| O | 0.668853000 | -4.044133000 | 0.913682000 |
| C | 0.190248000 | -2.464951000 | -0.826800000 |
| H | 1.193505000 | -2.204275000 | -1.171926000 |
| H | -0.165679000 | -3.310349000 | -1.426955000 |
| N | -0.688935000 | -1.285127000 | -0.939342000 |
| C | -0.590856000 | -0.484400000 | -2.192025000 |
| H | -0.532132000 | -1.129638000 | -3.078196000 |
| H | -1.520941000 | 0.081245000 | -2.274686000 |
| C | -2.101879000 | -1.650942000 | -0.680771000 |
| H | -2.637435000 | -1.882067000 | -1.607229000 |
| H | -2.132984000 | -2.547567000 | -0.059198000 |
| C | -2.828675000 | -0.548074000 | 0.101101000 |
| O | -4.069416000 | -0.550524000 | 0.126206000 |
| O | -2.067131000 | 0.283081000 | 0.704833000 |
| Cr | 0.000000000 | 0.000000000 | 0.637333000 |

EDTA Cr(III)

ΔG = -2144.591144

| | | | |
|---|---|---|---|
| C | -1.477832000 | 2.359038000 | 0.766000000 |
| O | -0.709132000 | 1.639298000 | 1.528320000 |
| O | -2.291079000 | 3.188422000 | 1.158266000 |
| C | -1.303323000 | 2.121830000 | -0.757556000 |
| H | -2.115694000 | 1.482239000 | -1.104503000 |
| H | -1.350309000 | 3.070214000 | -1.298710000 |
| N | 0.000000000 | 1.438107000 | -0.950717000 |
| C | 0.265017000 | 0.714724000 | -2.239752000 |
| H | -0.154860000 | 1.266727000 | -3.085468000 |
| H | 1.347325000 | 0.702973000 | -2.369026000 |
| C | 1.121559000 | 2.389597000 | -0.690656000 |
| H | 1.460476000 | 2.855226000 | -1.618268000 |
| H | 0.778217000 | 3.185426000 | -0.029351000 |
| C | 2.266300000 | 1.670136000 | 0.020801000 |
| O | 3.388232000 | 2.163037000 | 0.057039000 |
| O | 1.917695000 | 0.554496000 | 0.580917000 |
| C | 1.477832000 | -2.359038000 | 0.766000000 |
| O | 0.709132000 | -1.639298000 | 1.528320000 |
| O | 2.291079000 | -3.188422000 | 1.158266000 |
| C | 1.303323000 | -2.121830000 | -0.757556000 |
| H | 2.115694000 | -1.482239000 | -1.104503000 |
| H | 1.350309000 | -3.070214000 | -1.298710000 |
| N | 0.000000000 | -1.438107000 | -0.950717000 |
| C | -0.265017000 | -0.714724000 | -2.239752000 |
| H | 0.154860000 | -1.266727000 | -3.085468000 |
| H | -1.347325000 | -0.702973000 | -2.369026000 |
| C | -1.121559000 | -2.389597000 | -0.690656000 |
| H | -1.460476000 | -2.855226000 | -1.618268000 |
| H | -0.778217000 | -3.185426000 | -0.029351000 |
| C | -2.266300000 | -1.670136000 | 0.020801000 |
| O | -3.388232000 | -2.163037000 | 0.057039000 |
| O | -1.917695000 | -0.554496000 | 0.580917000 |
| Cr | 0.000000000 | 0.000000000 | 0.580917000 |

EDTA Cu(II)

ΔG = -2740.670145

| | | | |
|---|---|---|---|
| C | -0.225914000 | 2.873470000 | 0.376440000 |
| O | -0.094584000 | 1.995823000 | 1.294569000 |
| O | -0.534688000 | 4.063040000 | 0.566018000 |
| C | 0.000000000 | 2.408850000 | -1.077894000 |
| H | -0.988391000 | 2.190711000 | -1.491704000 |
| H | 0.408787000 | 3.251230000 | -1.653661000 |
| N | 0.835191000 | 1.214967000 | -1.152540000 |
| C | 0.656478000 | 0.394735000 | -2.355135000 |
| H | 0.697497000 | 0.996742000 | -3.277048000 |
| H | 1.497790000 | -0.299878000 | -2.403599000 |
| C | 2.236888000 | 1.478545000 | -0.820840000 |
| H | 2.893415000 | 1.320335000 | -1.682863000 |
| H | 2.364681000 | 2.528421000 | -0.538846000 |
| C | 2.800159000 | 0.654071000 | 0.353198000 |
| O | 4.027953000 | 0.704948000 | 0.541404000 |
| O | 1.986638000 | -0.015745000 | 1.076150000 |
| C | 0.225914000 | -2.873470000 | 0.376440000 |
| O | 0.094584000 | -1.995823000 | 1.294569000 |

| | | | |
|---|---|---|---|
| O | 0.534688000 | -4.063040000 | 0.566018000 |
| C | 0.000000000 | -2.408850000 | -1.077894000 |
| H | 0.988391000 | -2.190711000 | -1.491704000 |
| H | -0.408787000 | -3.251230000 | -1.653661000 |
| N | -0.835191000 | -1.214967000 | -1.152540000 |
| C | -0.656478000 | -0.394735000 | -2.355135000 |
| H | -0.697497000 | -0.996742000 | -3.277048000 |
| H | -1.497790000 | 0.299878000 | -2.403599000 |
| C | -2.236888000 | -1.478545000 | -0.820840000 |
| H | -2.893415000 | -1.320335000 | -1.682863000 |
| H | -2.364681000 | -2.528421000 | -0.538846000 |
| C | -2.800159000 | -0.654071000 | 0.353198000 |
| O | -4.027953000 | -0.704948000 | 0.541404000 |
| O | -1.986638000 | 0.015745000 | 1.076150000 |
| Cu | 0.000000000 | 0.000000000 | 0.857639000 |

EDTA Fe(II)

ΔG = -2363.901456

| | | | |
|---|---|---|---|
| C | -0.806342000 | 2.419850000 | -1.012091000 |
| O | -0.564087000 | 1.369057000 | -1.708592000 |
| O | -0.917603000 | 3.572041000 | -1.454515000 |
| C | -0.907778000 | 2.199795000 | 0.509774000 |
| H | 0.089011000 | 2.406586000 | 0.900482000 |
| H | -1.625262000 | 2.892108000 | 0.958873000 |
| N | -1.257144000 | 0.786754000 | 0.798749000 |
| C | -0.738422000 | 0.259979000 | 2.097879000 |
| H | -1.003350000 | 0.929000000 | 2.925340000 |
| H | -1.249487000 | -0.689190000 | 2.274180000 |
| C | -2.724258000 | 0.568185000 | 0.697156000 |
| H | -3.221424000 | 0.733771000 | 1.657127000 |
| H | -3.135004000 | 1.291925000 | -0.011061000 |
| C | -3.055829000 | -0.824459000 | 0.142321000 |
| O | -4.192067000 | -1.282579000 | 0.305212000 |
| O | -2.101954000 | -1.391419000 | -0.505558000 |
| C | 1.116418000 | -2.643763000 | -0.631819000 |
| O | 0.586851000 | -1.861861000 | -1.509057000 |
| O | 1.566310000 | -3.772230000 | -0.852765000 |
| C | 1.141072000 | -2.090088000 | 0.815250000 |
| H | 0.216528000 | -2.401974000 | 1.309055000 |
| H | 1.979599000 | -2.523914000 | 1.369855000 |
| N | 1.210663000 | -0.598819000 | 0.791084000 |
| C | 0.777219000 | 0.051160000 | 2.064742000 |
| H | 1.085859000 | -0.540635000 | 2.934340000 |
| H | 1.283489000 | 1.011909000 | 2.108347000 |
| C | 2.620340000 | -0.244773000 | 0.434809000 |
| H | 3.278916000 | -0.606087000 | 1.236773000 |
| H | 2.880090000 | -0.810041000 | -0.460171000 |
| C | 3.018113000 | 1.229912000 | 0.159743000 |
| O | 4.185226000 | 1.337817000 | -0.296363000 |
| O | 2.217719000 | 2.158416000 | 0.419462000 |
| Fe | -0.351537000 | -0.369143000 | -0.583891000 |

EDTA Fe(III)

ΔG = -2363.750482

| | | | |
|---|---|---|---|
| C | -1.454624000 | 2.304328000 | 0.764023000 |
| O | -0.676279000 | 1.567054000 | 1.496159000 |
| O | -2.246933000 | 3.138676000 | 1.188713000 |
| C | -1.308910000 | 2.075244000 | -0.760300000 |
| H | -2.119225000 | 1.430395000 | -1.099916000 |
| H | -1.357646000 | 3.021819000 | -1.303298000 |
| N | 0.000000000 | 1.397932000 | -0.958141000 |
| C | 0.267278000 | 0.709896000 | -2.264239000 |
| H | -0.154581000 | 1.281207000 | -3.095292000 |
| H | 1.349376000 | 0.698493000 | -2.393512000 |
| C | 1.109956000 | 2.364576000 | -0.689585000 |
| H | 1.462517000 | 2.815532000 | -1.618517000 |
| H | 0.751469000 | 3.168462000 | -0.047482000 |
| C | 2.234485000 | 1.652318000 | 0.050806000 |
| O | 3.357648000 | 2.140768000 | 0.123570000 |
| O | 1.869104000 | 0.538557000 | 0.597597000 |
| C | 1.454624000 | -2.304328000 | 0.764023000 |
| O | 0.676279000 | -1.567054000 | 1.496159000 |
| O | 2.246933000 | -3.138676000 | 1.188713000 |
| C | 1.308910000 | -2.075244000 | -0.760300000 |
| H | 2.119225000 | -1.430395000 | -1.099916000 |
| H | 1.357646000 | -3.021819000 | -1.303298000 |
| N | 0.000000000 | -1.397932000 | -0.958141000 |
| C | -0.267278000 | -0.709896000 | -2.264239000 |
| H | 0.154581000 | -1.281207000 | -3.095292000 |
| H | -1.349376000 | -0.698493000 | -2.393512000 |
| C | -1.109956000 | -2.364576000 | -0.689585000 |
| H | -1.462517000 | -2.815532000 | -1.618517000 |
| H | -0.751469000 | -3.168462000 | -0.047482000 |
| C | -2.234485000 | -1.652318000 | 0.050806000 |
| O | -3.357648000 | -2.140768000 | 0.123570000 |
| O | -1.869104000 | -0.538557000 | 0.597597000 |
| Fe | 0.000000000 | 0.000000000 | 0.493266000 |

EDTA K(I)

ΔG = -1700.279805

| | | | |
|---|---|---|---|
| C | 3.392361000 | -0.867552000 | -0.794682000 |
| O | 2.755068000 | -0.453995000 | -1.798094000 |
| O | 4.570322000 | -1.317305000 | -0.790264000 |
| C | 2.664765000 | -0.908552000 | 0.576821000 |
| H | 2.235047000 | -1.912881000 | 0.647992000 |
| H | 3.426741000 | -0.835396000 | 1.370957000 |

| | | | |
|---|---|---|---|
| N | 1.584649000 | 0.063462000 | 0.755940000 |
| C | 0.687426000 | -0.338679000 | 1.847475000 |
| H | 0.553615000 | -1.419939000 | 1.791492000 |
| H | 1.152222000 | -0.131797000 | 2.830903000 |
| C | 2.122099000 | 1.415379000 | 0.941568000 |
| H | 2.383767000 | 1.595803000 | 1.997056000 |
| H | 3.055318000 | 1.488374000 | 0.373619000 |
| C | 1.270073000 | 2.614002000 | 0.439376000 |
| O | 1.410406000 | 3.685197000 | 1.090896000 |
| O | 0.572081000 | 2.458263000 | -0.595398000 |
| C | -3.392351000 | 0.867571000 | -0.794682000 |
| O | -2.755060000 | 0.454011000 | -1.798094000 |
| O | -4.570305000 | 1.317340000 | -0.790264000 |
| C | -2.664759000 | 0.908553000 | 0.576825000 |
| H | -2.235036000 | 1.912879000 | 0.648008000 |
| H | -3.426739000 | 0.835393000 | 1.370957000 |
| N | -1.584650000 | -0.063469000 | 0.755938000 |
| C | -0.687427000 | 0.338656000 | 1.847480000 |
| H | -0.553616000 | 1.419917000 | 1.791512000 |
| H | -1.152224000 | 0.131761000 | 2.830904000 |
| C | -2.122109000 | -1.415384000 | 0.941552000 |
| H | -2.383795000 | -1.595812000 | 1.997035000 |
| H | -3.055321000 | -1.488371000 | 0.373588000 |
| C | -1.270083000 | -2.614008000 | 0.439366000 |
| O | -1.410417000 | -3.685202000 | 1.090887000 |
| O | -0.572087000 | -2.458271000 | -0.595406000 |
| K | 0.000000000 | -0.000005000 | -1.644625000 |

EDTA Li(I)

ΔG = -1107.883008

| | | | |
|---|---|---|---|
| C | -2.395817000 | 1.201730000 | 1.087010000 |
| O | -1.548372000 | 0.785611000 | 1.937073000 |
| O | -3.555082000 | 1.602023000 | 1.333739000 |
| C | -1.960760000 | 1.138076000 | -0.396049000 |
| H | -2.216559000 | 0.126476000 | -0.723180000 |
| H | -2.555413000 | 1.848224000 | -0.989097000 |
| N | -0.514210000 | 1.345987000 | -0.572501000 |
| C | 0.000000000 | 0.767385000 | -1.824668000 |
| H | -0.577896000 | 1.112040000 | -2.699879000 |
| H | 1.016699000 | 1.139083000 | -1.948173000 |
| C | -0.208589000 | 2.779574000 | -0.477918000 |
| H | -0.636114000 | 3.328503000 | -1.333106000 |
| H | -0.706659000 | 3.171324000 | 0.414829000 |
| C | 1.280419000 | 3.194699000 | -0.349128000 |
| O | 1.482529000 | 4.431780000 | -0.499230000 |
| O | 2.142124000 | 2.320902000 | -0.088587000 |
| C | 2.395817000 | -1.201730000 | 1.087010000 |
| O | 1.548372000 | -0.785611000 | 1.937073000 |
| O | 3.555082000 | -1.602023000 | 1.333739000 |
| C | 1.960760000 | -1.138076000 | -0.396049000 |
| H | 2.216559000 | -0.126476000 | -0.723180000 |
| H | 2.555413000 | -1.848224000 | -0.989097000 |
| N | 0.514210000 | -1.345987000 | -0.572501000 |
| C | 0.000000000 | -0.767385000 | -1.824668000 |
| H | 0.577896000 | -1.112040000 | -2.699879000 |
| H | -1.016699000 | -1.139083000 | -1.948173000 |
| C | 0.208589000 | -2.779574000 | -0.477918000 |
| H | 0.636114000 | -3.328503000 | -1.333106000 |
| H | 0.706659000 | -3.171324000 | 0.414829000 |
| C | -1.280419000 | -3.194699000 | -0.349128000 |
| O | -1.482529000 | -4.431780000 | -0.499230000 |
| O | -2.142124000 | -2.320902000 | -0.088587000 |
| Li | 0.000000000 | 0.000000000 | 1.057774000 |

EDTA Mg(II)

ΔG = -1300.400923

| | | | |
|---|---|---|---|
| C | -1.755553000 | 2.314459000 | 0.580830000 |
| O | -1.099980000 | 1.695484000 | 1.480550000 |
| O | -2.685615000 | 3.119171000 | 0.769079000 |
| C | -1.357747000 | 2.012630000 | -0.886441000 |
| H | -2.075329000 | 1.282241000 | -1.270135000 |
| H | -1.468997000 | 2.922932000 | -1.488963000 |
| N | 0.000000000 | 1.453738000 | -0.954677000 |
| C | 0.312027000 | 0.697577000 | -2.183435000 |
| H | -0.006856000 | 1.235818000 | -3.087413000 |
| H | 1.399360000 | 0.610170000 | -2.240387000 |
| C | 1.022975000 | 2.479707000 | -0.676605000 |
| H | 1.480581000 | 2.856588000 | -1.597461000 |
| H | 0.552524000 | 3.338164000 | -0.190655000 |
| C | 2.124365000 | 1.997229000 | 0.287569000 |
| O | 3.172918000 | 2.666007000 | 0.341883000 |
| O | 1.854806000 | 0.966566000 | 0.983007000 |
| C | 1.755553000 | -2.314459000 | 0.580830000 |
| O | 1.099980000 | -1.695484000 | 1.480550000 |
| O | 2.685615000 | -3.119171000 | 0.769079000 |
| C | 1.357747000 | -2.012630000 | -0.886441000 |
| H | 2.075329000 | -1.282241000 | -1.270135000 |
| H | 1.468997000 | -2.922932000 | -1.488963000 |
| N | 0.000000000 | -1.453738000 | -0.954677000 |
| C | -0.312027000 | -0.697577000 | -2.183435000 |
| H | 0.006856000 | -1.235818000 | -3.087413000 |
| H | -1.399360000 | -0.610170000 | -2.240387000 |
| C | -1.022975000 | -2.479707000 | -0.676605000 |
| H | -1.480581000 | -2.856588000 | -1.597461000 |
| H | -0.552524000 | -3.338164000 | -0.190655000 |
| C | -2.124365000 | -1.997229000 | 0.287569000 |

| | | | |
|---|---|---|---|
| O | -3.172918000 | -2.666007000 | 0.341883000 |
| O | -1.854806000 | -0.966566000 | 0.983007000 |
| Mg | 0.000000000 | 0.000000000 | 0.871684000 |

EDTA Mn(II)

ΔG = -2251.190128

| | | | |
|---|---|---|---|
| C | -1.529869000 | 2.357161000 | 0.919424000 |
| O | -0.884544000 | 1.576279000 | 1.707300000 |
| O | -2.269975000 | 3.293911000 | 1.232872000 |
| C | -1.340172000 | 2.056478000 | -0.603481000 |
| H | -2.109499000 | 1.332711000 | -0.880063000 |
| H | -1.464685000 | 2.979041000 | -1.185155000 |
| N | 0.000000000 | 1.454695000 | -0.804493000 |
| C | 0.255261000 | 0.723692000 | -2.073527000 |
| H | -0.180839000 | 1.256669000 | -2.933825000 |
| H | 1.337307000 | 0.720554000 | -2.210476000 |
| C | 1.056939000 | 2.478575000 | -0.610002000 |
| H | 1.138311000 | 3.121153000 | -1.497559000 |
| H | 0.770457000 | 3.107877000 | 0.234443000 |
| C | 2.433709000 | 1.846465000 | -0.273712000 |
| O | 3.438412000 | 2.536367000 | -0.533426000 |
| O | 2.365148000 | 0.698167000 | 0.245558000 |
| C | 1.529869000 | -2.357161000 | 0.919424000 |
| O | 0.884544000 | -1.576279000 | 1.707300000 |
| O | 2.269975000 | -3.293911000 | 1.232872000 |
| C | 1.340172000 | -2.056478000 | -0.603481000 |
| H | 2.109499000 | -1.332711000 | -0.880063000 |
| H | 1.464685000 | -2.979041000 | -1.185155000 |
| N | 0.000000000 | -1.454695000 | -0.804493000 |
| C | -0.255261000 | -0.723692000 | -2.073527000 |
| H | 0.180839000 | -1.256669000 | -2.933825000 |
| H | -1.337307000 | -0.720554000 | -2.210476000 |
| C | -1.056939000 | -2.478575000 | -0.610002000 |
| H | -1.138311000 | -3.121153000 | -1.497559000 |
| H | -0.770457000 | -3.107877000 | 0.234443000 |
| C | -2.433709000 | -1.846465000 | -0.273712000 |
| O | -3.438412000 | -2.536367000 | -0.533426000 |
| O | -2.365148000 | -0.698167000 | 0.245558000 |
| Mn | 0.000000000 | 0.000000000 | 0.698675000 |

EDTA Na(I)

ΔG = -1262.648833

| | | | |
|---|---|---|---|
| C | -0.223763000 | 3.167909000 | 0.538721000 |
| O | 0.000000000 | 2.491859000 | 1.576611000 |
| O | -0.636917000 | 4.357540000 | 0.492917000 |
| C | -0.021687000 | 2.483138000 | -0.841381000 |
| H | -1.021754000 | 2.204685000 | -1.185397000 |
| H | 0.352172000 | 3.238051000 | -1.553364000 |
| N | 0.822430000 | 1.292283000 | -0.802749000 |
| C | 0.653203000 | 0.401595000 | -1.956676000 |
| H | 0.708493000 | 0.957754000 | -2.910725000 |
| H | 1.500506000 | -0.288942000 | -1.962264000 |
| C | 2.229383000 | 1.633792000 | -0.593474000 |
| H | 2.740999000 | 1.865915000 | -1.540863000 |
| H | 2.277065000 | 2.541006000 | 0.016089000 |
| C | 3.060669000 | 0.579600000 | 0.181659000 |
| O | 4.301080000 | 0.589140000 | -0.042124000 |
| O | 2.453144000 | -0.154224000 | 1.004019000 |
| C | 0.223763000 | -3.167909000 | 0.538721000 |
| O | 0.000000000 | -2.491859000 | 1.576611000 |
| O | 0.636917000 | -4.357540000 | 0.492917000 |
| C | 0.021687000 | -2.483138000 | -0.841381000 |
| H | 1.021754000 | -2.204685000 | -1.185397000 |
| H | -0.352172000 | -3.238051000 | -1.553364000 |
| N | -0.822430000 | -1.292283000 | -0.802749000 |
| C | -0.653203000 | -0.401595000 | -1.956676000 |
| H | -0.708493000 | -0.957754000 | -2.910725000 |
| H | -1.500506000 | 0.288942000 | -1.962264000 |
| C | -2.229383000 | -1.633792000 | -0.593474000 |
| H | -2.740999000 | -1.865915000 | -1.540863000 |
| H | -2.277065000 | -2.541006000 | 0.016089000 |
| C | -3.060669000 | -0.579600000 | 0.181659000 |
| O | -4.301080000 | -0.589140000 | -0.042124000 |
| O | -2.453144000 | 0.154224000 | 1.004019000 |
| Na | 0.000000000 | 0.000000000 | 1.187508000 |

EDTA Ni(II)

ΔG = -2608.484744

| | | | |
|---|---|---|---|
| C | -0.926894000 | 2.415074000 | 1.336422000 |
| O | -0.313992000 | 1.417187000 | 1.895749000 |
| O | -1.400834000 | 3.376620000 | 1.940374000 |
| C | -1.067009000 | 2.304127000 | -0.190737000 |
| H | -2.035595000 | 1.847940000 | -0.396401000 |
| H | -1.014227000 | 3.287654000 | -0.664287000 |
| N | 0.000000000 | 1.385545000 | -0.688285000 |
| C | -0.321616000 | 0.694407000 | -1.981567000 |
| H | -1.403137000 | 0.613362000 | -2.049824000 |
| H | 0.035970000 | 1.283365000 | -2.831103000 |
| C | 1.276617000 | 2.169437000 | -0.777554000 |
| H | 1.205686000 | 2.828547000 | -1.646989000 |
| H | 1.329526000 | 2.803883000 | 0.110409000 |
| C | 2.626333000 | 1.405843000 | -0.819958000 |
| O | 3.539118000 | 2.003775000 | -1.439567000 |
| O | 2.710184000 | 0.322301000 | -0.192076000 |
| C | 0.926894000 | -2.415074000 | 1.336422000 |
| O | 0.313992000 | -1.417187000 | 1.895749000 |

| | | | |
|---|---|---|---|
| O | 1.400834000 | -3.376620000 | 1.940374000 |
| C | 1.067009000 | -2.304127000 | -0.190737000 |
| H | 2.035595000 | -1.847940000 | -0.396401000 |
| H | 1.014227000 | -3.287654000 | -0.664287000 |
| N | 0.000000000 | -1.385545000 | -0.688285000 |
| C | 0.321616000 | -0.694407000 | -1.981567000 |
| H | 1.403137000 | -0.613362000 | -2.049824000 |
| H | -0.035970000 | -1.283365000 | -2.831103000 |
| C | -1.276617000 | -2.169437000 | -0.777554000 |
| H | -1.205686000 | -2.828547000 | -1.646989000 |
| H | -1.329526000 | -2.803883000 | 0.110409000 |
| C | -2.626333000 | -1.405843000 | -0.819958000 |
| O | -3.539118000 | -2.003775000 | -1.439567000 |
| O | -2.710184000 | -0.322301000 | -0.192076000 |
| Ni | 0.000000000 | 0.000000000 | 0.661480000 |

EDTA V(II)

ΔG = -2044.230718

| | | | |
|---|---|---|---|
| C | -0.233302000 | 2.936012000 | 0.580076000 |
| O | 0.000000000 | 2.051859000 | 1.472105000 |
| O | -0.576529000 | 4.112550000 | 0.785175000 |
| C | -0.086465000 | 2.467593000 | -0.889955000 |
| H | -1.086657000 | 2.230058000 | -1.260742000 |
| H | 0.310923000 | 3.284403000 | -1.504158000 |
| N | 0.758196000 | 1.259752000 | -0.945233000 |
| C | 0.627289000 | 0.434189000 | -2.173514000 |
| H | 0.636603000 | 1.057688000 | -3.077441000 |
| H | 1.512249000 | -0.203630000 | -2.222296000 |
| C | 2.179971000 | 1.594251000 | -0.698201000 |
| H | 2.726230000 | 1.755128000 | -1.633515000 |
| H | 2.234730000 | 2.527268000 | -0.133280000 |
| C | 2.885784000 | 0.527470000 | 0.154515000 |
| O | 4.128188000 | 0.510581000 | 0.167995000 |
| O | 2.116245000 | -0.241514000 | 0.822639000 |
| C | 0.233302000 | -2.936012000 | 0.580076000 |
| O | 0.000000000 | -2.051859000 | 1.472105000 |
| O | 0.576529000 | -4.112550000 | 0.785175000 |
| C | 0.086465000 | -2.467593000 | -0.889955000 |
| H | 1.086657000 | -2.230058000 | -1.260742000 |
| H | -0.310923000 | -3.284403000 | -1.504158000 |
| N | -0.758196000 | -1.259752000 | -0.945233000 |
| C | -0.627289000 | -0.434189000 | -2.173514000 |
| H | -0.636603000 | -1.057688000 | -3.077441000 |
| H | -1.512249000 | 0.203630000 | -2.222296000 |
| C | -2.179971000 | -1.594251000 | -0.698201000 |
| H | -2.726230000 | -1.755128000 | -1.633515000 |
| H | -2.234730000 | -2.527268000 | -0.133280000 |
| C | -2.885784000 | -0.527470000 | 0.154515000 |
| O | -4.128188000 | -0.510581000 | 0.167995000 |
| O | -2.116245000 | 0.241514000 | 0.822639000 |
| V | 0.000000000 | 0.000000000 | 0.750194000 |

EDTA V(III)

ΔG = -2044.090265

| | | | |
|---|---|---|---|
| C | -2.123270000 | 1.875022000 | -0.559059000 |
| O | -1.526600000 | 1.052757000 | -1.381704000 |
| O | -2.785718000 | 2.845401000 | -0.892407000 |
| C | -1.908404000 | 1.513491000 | 0.917295000 |
| H | -1.163456000 | 2.194352000 | 1.333105000 |
| H | -2.835458000 | 1.641681000 | 1.484051000 |
| N | -1.406455000 | 0.119688000 | 0.961690000 |
| C | -0.710177000 | -0.259332000 | 2.222718000 |
| H | -1.256200000 | 0.110764000 | 3.096386000 |
| H | -0.714782000 | -1.348686000 | 2.278528000 |
| C | -2.530117000 | -0.834560000 | 0.713381000 |
| H | -2.950662000 | -1.194153000 | 1.654622000 |
| H | -3.329332000 | -0.319158000 | 0.177897000 |
| C | -2.087831000 | -2.001354000 | -0.172068000 |
| O | -2.747877000 | -3.041548000 | -0.188353000 |
| O | -1.050752000 | -1.755147000 | -0.892540000 |
| C | 2.123263000 | -1.875028000 | -0.559058000 |
| O | 1.526589000 | -1.052763000 | -1.381704000 |
| O | 2.785705000 | -2.845409000 | -0.892407000 |
| C | 1.908408000 | -1.513489000 | 0.917297000 |
| H | 1.163460000 | -2.194346000 | 1.333112000 |
| H | 2.835466000 | -1.641681000 | 1.484046000 |
| N | 1.406465000 | -0.119685000 | 0.961689000 |
| C | 0.710191000 | 0.259347000 | 2.222714000 |
| H | 1.256214000 | -0.110740000 | 3.096386000 |
| H | 0.714797000 | 1.348702000 | 2.278514000 |
| C | 2.530126000 | 0.834560000 | 0.713367000 |
| H | 2.950685000 | 1.194150000 | 1.654603000 |
| H | 3.329332000 | 0.319155000 | 0.177872000 |
| C | 2.087831000 | 2.001354000 | -0.172077000 |
| O | 2.747870000 | 3.041552000 | -0.188361000 |
| O | 1.050752000 | 1.755142000 | -0.892547000 |
| V | -0.000001000 | 0.000000000 | -0.752158000 |

EDTA Zn(II)

ΔG = -2879.561167

| | | | |
|---|---|---|---|
| C | 2.884977000 | -0.982581000 | 1.085463000 |
| O | 2.239743000 | -1.506826000 | 0.115758000 |
| O | 3.916429000 | -1.412205000 | 1.613972000 |
| C | 2.267589000 | 0.302767000 | 1.703836000 |
| H | 1.720511000 | -0.029356000 | 2.591294000 |
| H | 3.081740000 | 0.948082000 | 2.057587000 |

| | | | |
|---|---|---|---|
| N | 1.348600000 | 1.014303000 | 0.809548000 |
| C | 0.138623000 | 1.530880000 | 1.472195000 |
| H | 0.384353000 | 2.027430000 | 2.425277000 |
| H | -0.314870000 | 2.286280000 | 0.829122000 |
| C | 2.020788000 | 1.975114000 | -0.078842000 |
| H | 1.842893000 | 3.009979000 | 0.236298000 |
| H | 3.100616000 | 1.809561000 | -0.023646000 |
| C | 1.669598000 | 1.872000000 | -1.592071000 |
| O | 2.050610000 | 2.806863000 | -2.299740000 |
| O | 1.059714000 | 0.816058000 | -1.986227000 |
| C | -3.502757000 | 1.078583000 | 0.444777000 |
| O | -4.577610000 | 1.385861000 | -0.293160000 |
| O | -3.390383000 | 2.104674000 | 1.300242000 |
| C | -2.258216000 | 0.634935000 | -0.315108000 |
| H | -2.563543000 | 0.057459000 | -1.189905000 |
| H | -1.707764000 | 1.514280000 | -0.687253000 |
| N | -1.291935000 | -0.222235000 | 0.460963000 |
| C | -0.883159000 | 0.418171000 | 1.729799000 |
| H | -1.744066000 | 0.835166000 | 2.262360000 |
| H | -0.431768000 | -0.354099000 | 2.360574000 |
| C | -1.831522000 | -1.589728000 | 0.617517000 |
| H | -1.382243000 | -2.053913000 | 1.501745000 |
| H | -2.914616000 | -1.571256000 | 0.766915000 |
| C | -1.489974000 | -2.520128000 | -0.580133000 |
| O | -2.161176000 | -3.546182000 | -0.705294000 |
| O | -0.503073000 | -2.162668000 | -1.323004000 |
| Zn | 0.578747000 | -0.607626000 | -0.677630000 |

NTP

ΔG = -856.858734

| | | | |
|---|---|---|---|
| O | -2.385782000 | 4.270424000 | 1.428849000 |
| O | 3.854655000 | -1.661436000 | -0.033913000 |
| O | -3.339688000 | -2.438816000 | 1.644167000 |
| C | -1.003363000 | -0.847586000 | 1.206990000 |
| C | -1.426350000 | -1.970025000 | 0.257806000 |
| C | -2.514987000 | -2.915577000 | 0.817777000 |
| C | 1.337363000 | -0.498428000 | 0.635845000 |
| C | 3.685357000 | -0.496032000 | -0.483871000 |
| C | -0.052164000 | 1.388585000 | 1.297924000 |
| C | -1.213245000 | 2.273523000 | 0.842936000 |
| C | -1.271673000 | 3.677416000 | 1.489085000 |
| N | -0.018110000 | 0.078407000 | 0.623616000 |
| O | 4.572219000 | 0.221787000 | -1.026547000 |
| O | -0.215492000 | 4.142522000 | 1.996507000 |
| O | -2.513992000 | -4.098309000 | 0.374255000 |
| C | 2.267018000 | 0.116926000 | -0.411075000 |
| H | -0.086337000 | 1.257151000 | 2.394905000 |
| H | 0.879533000 | 1.912597000 | 1.086058000 |
| H | -1.138599000 | 2.423262000 | -0.241590000 |
| H | -2.175794000 | 1.785144000 | 1.013468000 |
| H | 1.788141000 | -0.405331000 | 1.641051000 |
| H | 1.812485000 | -0.009135000 | -1.401907000 |
| H | 2.375336000 | 1.194401000 | -0.264641000 |
| H | 1.259703000 | -1.566981000 | 0.436739000 |
| H | -1.830510000 | -1.519206000 | -0.657778000 |
| H | -0.572603000 | -2.577745000 | -0.049909000 |
| H | -0.617136000 | -1.278988000 | 2.149424000 |
| H | -1.894231000 | -0.281027000 | 1.475814000 |

NTP Al(III)

ΔG = -1099.072397

| | | | |
|---|---|---|---|
| Al | 0.122183000 | -0.185893000 | -0.894401000 |
| O | -0.411880000 | 1.417531000 | -1.383907000 |
| O | 1.781233000 | -0.554987000 | -1.332485000 |
| O | -1.005148000 | -1.486647000 | -1.257385000 |
| C | -0.970821000 | -0.965043000 | 1.656453000 |
| C | -2.234383000 | -1.167455000 | 0.805046000 |
| C | -2.034729000 | -1.898475000 | -0.520117000 |
| C | 1.467233000 | -0.607801000 | 1.574675000 |
| C | 2.890120000 | -0.486400000 | -0.599622000 |
| C | -0.022189000 | 1.359451000 | 1.510696000 |
| C | -1.110873000 | 2.181628000 | 0.812752000 |
| C | -0.931866000 | 2.421820000 | -0.684742000 |
| N | 0.128113000 | -0.084945000 | 1.070245000 |
| O | 3.982851000 | -0.716690000 | -1.070981000 |
| O | -1.271879000 | 3.459923000 | -1.211012000 |
| O | -2.776063000 | -2.782315000 | -0.892130000 |
| C | 2.711339000 | -0.069637000 | 0.858170000 |
| H | -0.205812000 | 1.355889000 | 2.587057000 |
| H | 0.938226000 | 1.846722000 | 1.348914000 |
| H | -2.103056000 | 1.747206000 | 0.961240000 |
| H | -1.139394000 | 3.157273000 | 1.299564000 |
| H | 1.534247000 | -0.375660000 | 2.640126000 |
| H | 2.763519000 | 1.023182000 | 0.892486000 |
| H | 3.581845000 | -0.424489000 | 1.411088000 |
| H | 1.440003000 | -1.692620000 | 1.475518000 |
| H | -2.931720000 | -1.751837000 | 1.406063000 |
| H | -2.740768000 | -0.223100000 | 0.588003000 |
| H | -0.525023000 | -1.941084000 | 1.842052000 |
| H | -1.251888000 | -0.546783000 | 2.624190000 |

NTP Be(II)

ΔG = -871.529719

| | | | |
|---|---|---|---|
| Be | 0.118668000 | -0.174950000 | -0.717336000 |
| O | -0.584340000 | 1.167640000 | -1.283168000 |

| | | | |
|---|---|---|---|
| O | 1.627064000 | -0.297429000 | -1.253679000 |
| O | -0.708496000 | -1.499922000 | -1.120608000 |
| C | -1.046827000 | -0.798575000 | 1.668233000 |
| C | -2.204833000 | -1.070368000 | 0.698468000 |
| C | -1.795453000 | -1.885856000 | -0.527684000 |
| C | 1.392720000 | -0.725703000 | 1.555099000 |
| C | 2.749068000 | -0.290905000 | -0.610595000 |
| C | 0.121119000 | 1.374755000 | 1.461956000 |
| C | -1.033902000 | 2.175580000 | 0.864587000 |
| C | -1.106995000 | 2.177162000 | -0.665329000 |
| N | 0.131146000 | -0.069673000 | 1.070756000 |
| O | 3.849549000 | -0.324359000 | -1.165213000 |
| O | -1.670232000 | 3.109661000 | -1.242507000 |
| O | -2.477647000 | -2.838903000 | -0.907653000 |
| C | 2.680942000 | -0.208390000 | 0.916243000 |
| H | 0.100682000 | 1.448770000 | 2.555469000 |
| H | 1.058449000 | 1.815178000 | 1.123338000 |
| H | -1.999255000 | 1.825644000 | 1.243674000 |
| H | -0.943661000 | 3.210970000 | 1.198553000 |
| H | 1.454971000 | -0.621790000 | 2.644745000 |
| H | 2.885199000 | 0.829383000 | 1.196674000 |
| H | 3.512357000 | -0.789308000 | 1.320854000 |
| H | 1.292096000 | -1.789410000 | 1.332970000 |
| H | -2.984510000 | -1.609180000 | 1.238846000 |
| H | -2.650596000 | -0.137292000 | 0.342601000 |
| H | -0.684752000 | -1.755908000 | 2.044377000 |
| H | -1.409111000 | -0.238056000 | 2.533885000 |

NTP B(III)

ΔG = -881.410107

| | | | |
|---|---|---|---|
| B | 0.070017000 | -0.111478000 | -0.570820000 |
| O | -0.813407000 | 0.912263000 | -1.127370000 |
| O | 1.395077000 | 0.084144000 | -1.142987000 |
| O | -0.406500000 | -1.434507000 | -0.963549000 |
| C | -1.139258000 | -0.681278000 | 1.662048000 |
| C | -2.178932000 | -1.142897000 | 0.636939000 |
| C | -1.561801000 | -1.931236000 | -0.500073000 |
| C | 1.315592000 | -0.807270000 | 1.527181000 |
| C | 2.590013000 | -0.145983000 | -0.595523000 |
| C | 0.211101000 | 1.420170000 | 1.460810000 |
| C | -0.918552000 | 2.265271000 | 0.886111000 |
| C | -1.144743000 | 2.096038000 | -0.603963000 |
| N | 0.103788000 | -0.029452000 | 1.046022000 |
| O | 3.593425000 | -0.126910000 | -1.272910000 |
| O | -1.671548000 | 2.946947000 | -1.284698000 |
| O | -2.040060000 | -2.939350000 | -0.967430000 |
| C | 2.626027000 | -0.342532000 | 0.906823000 |
| H | 0.217689000 | 1.463688000 | 2.550491000 |
| H | 1.167705000 | 1.795433000 | 1.103064000 |
| H | -1.872944000 | 2.041671000 | 1.373853000 |
| H | -0.714502000 | 3.316966000 | 1.086807000 |
| H | 1.352980000 | -0.728214000 | 2.614131000 |
| H | 2.972269000 | 0.598645000 | 1.343358000 |
| H | 3.397129000 | -1.079755000 | 1.134461000 |
| H | 1.129164000 | -1.848393000 | 1.266835000 |
| H | -2.912419000 | -1.769045000 | 1.143494000 |
| H | -2.719479000 | -0.297654000 | 0.203598000 |
| H | -0.793456000 | -1.538145000 | 2.236130000 |
| H | -1.580958000 | 0.021626000 | 2.364722000 |

NTP Ca(II)

ΔG = -1534.405089

| | | | |
|---|---|---|---|
| Ca | 0.188943000 | -0.293071000 | -1.537647000 |
| O | -0.799160000 | 1.738000000 | -1.320203000 |
| O | 2.395905000 | -0.612004000 | -1.144740000 |
| O | -1.271631000 | -1.966682000 | -1.045634000 |
| C | -0.995974000 | -0.722849000 | 1.700645000 |
| C | -2.272446000 | -0.905675000 | 0.856020000 |
| C | -2.184863000 | -2.062869000 | -0.151772000 |
| C | 1.401830000 | -0.699092000 | 1.586659000 |
| C | 3.255906000 | -0.290074000 | -0.256210000 |
| C | 0.195047000 | 1.405806000 | 1.375635000 |
| C | -1.044208000 | 2.236941000 | 1.033871000 |
| C | -1.392460000 | 2.460764000 | -0.448993000 |
| N | 0.166408000 | -0.046507000 | 1.047574000 |
| O | 4.463993000 | -0.087423000 | -0.476940000 |
| O | -2.242467000 | 3.332099000 | -0.708270000 |
| O | -2.981124000 | -3.013711000 | -0.052067000 |
| C | 2.769327000 | -0.131057000 | 1.194179000 |
| H | 0.381739000 | 1.519113000 | 2.456385000 |
| H | 1.049185000 | 1.845883000 | 0.859176000 |
| H | -1.939349000 | 1.848035000 | 1.527810000 |
| H | -0.891538000 | 3.228047000 | 1.471692000 |
| H | 1.351948000 | -0.678079000 | 2.687255000 |
| H | 2.858006000 | 0.927834000 | 1.453366000 |
| H | 3.507028000 | -0.634756000 | 1.826145000 |
| H | 1.357905000 | -1.749153000 | 1.287687000 |
| H | -3.099197000 | -1.106480000 | 1.540478000 |
| H | -2.518719000 | 0.009433000 | 0.312090000 |
| H | -0.666252000 | -1.714224000 | 2.018551000 |
| H | -1.251577000 | -0.182275000 | 2.621915000 |

NTP Co(II)

ΔG = -2239.423535

| | | | |
|---|---|---|---|
| Co | 0.133854000 | -0.276550000 | -0.944932000 |
| O | -0.546707000 | 1.637212000 | -1.116555000 |

| | | | |
|---|---|---|---|
| O | 2.022653000 | -0.033422000 | -1.206869000 |
| O | -1.523364000 | -1.197678000 | -1.253446000 |
| C | -0.999901000 | -1.044523000 | 1.671802000 |
| C | -2.350679000 | -0.839465000 | 0.973448000 |
| C | -2.471932000 | -1.462754000 | -0.417610000 |
| C | 1.398680000 | -1.202570000 | 1.437235000 |
| C | 3.028834000 | -0.313876000 | -0.446064000 |
| C | 0.385921000 | 1.022421000 | 1.590030000 |
| C | -0.700957000 | 2.051537000 | 1.278900000 |
| C | -0.958955000 | 2.424934000 | -0.194245000 |
| N | 0.200618000 | -0.371690000 | 1.070813000 |
| O | 4.200401000 | -0.268506000 | -0.836065000 |
| O | -1.570466000 | 3.486092000 | -0.407274000 |
| O | -3.457009000 | -2.153067000 | -0.702513000 |
| C | 2.769813000 | -0.664844000 | 1.024959000 |
| H | 0.510603000 | 0.963477000 | 2.679339000 |
| H | 1.321610000 | 1.392178000 | 1.175028000 |
| H | -1.658126000 | 1.784226000 | 1.732661000 |
| H | -0.401003000 | 2.973901000 | 1.783922000 |
| H | 1.403853000 | -1.344605000 | 2.525706000 |
| H | 3.037577000 | 0.221899000 | 1.607204000 |
| H | 3.504363000 | -1.422786000 | 1.307218000 |
| H | 1.240198000 | -2.183276000 | 0.984311000 |
| H | -3.115197000 | -1.284307000 | 1.613061000 |
| H | -2.600345000 | 0.218239000 | 0.876954000 |
| H | -0.788126000 | -2.115018000 | 1.686062000 |
| H | -1.082806000 | -0.725462000 | 2.717053000 |

NTP Co(III)

ΔG = -2239.22284

| | | | |
|---|---|---|---|
| Co | 0.080071000 | -0.258316000 | -0.897428000 |
| O | -0.474737000 | 1.469072000 | -0.933425000 |
| O | 1.880603000 | 0.150717000 | -1.122193000 |
| O | -1.584303000 | -0.960742000 | -1.261036000 |
| C | -1.068979000 | -1.162948000 | 1.557010000 |
| C | -2.412922000 | -0.723289000 | 0.979830000 |
| C | -2.624661000 | -1.098777000 | -0.479909000 |
| C | 1.314630000 | -1.521667000 | 1.172648000 |
| C | 2.930140000 | -0.313748000 | -0.494769000 |
| C | 0.532424000 | 0.770552000 | 1.676688000 |
| C | -0.432455000 | 1.926371000 | 1.466264000 |
| C | -0.665594000 | 2.349689000 | 0.023289000 |
| N | 0.186184000 | -0.525141000 | 0.987182000 |
| O | 4.064923000 | -0.159054000 | -0.925399000 |
| O | -1.050581000 | 3.475571000 | -0.250620000 |
| O | -3.703770000 | -1.499051000 | -0.892128000 |
| C | 2.716645000 | -1.021108000 | 0.844308000 |
| H | 0.617461000 | 0.547948000 | 2.743513000 |
| H | 1.512348000 | 1.072469000 | 1.320982000 |
| H | -1.405250000 | 1.749563000 | 1.928859000 |
| H | -0.017793000 | 2.791741000 | 1.987327000 |
| H | 1.283446000 | -1.849828000 | 2.215524000 |
| H | 3.094157000 | -0.350986000 | 1.620814000 |
| H | 3.379451000 | -1.888853000 | 0.863030000 |
| H | 1.064939000 | -2.390988000 | 0.557824000 |
| H | -3.190802000 | -1.209546000 | 1.570640000 |
| H | -2.584796000 | 0.349281000 | 1.075715000 |
| H | -0.960959000 | -2.236868000 | 1.409347000 |
| H | -1.049862000 | -0.975942000 | 2.632954000 |

NTP Cr(II)

ΔG = -1901.138845

| | | | |
|---|---|---|---|
| Cr | 0.121480000 | -0.286493000 | -1.082600000 |
| O | -0.458638000 | 1.709706000 | -1.096503000 |
| O | 2.095015000 | -0.060904000 | -1.223796000 |
| O | -1.654619000 | -1.142190000 | -1.290702000 |
| C | -1.005439000 | -1.093785000 | 1.625844000 |
| C | -2.375008000 | -0.828967000 | 0.979867000 |
| C | -2.577616000 | -1.401214000 | -0.425063000 |
| C | 1.386115000 | -1.272927000 | 1.386499000 |
| C | 3.078171000 | -0.352965000 | -0.442938000 |
| C | 0.405775000 | 0.955299000 | 1.599457000 |
| C | -0.651500000 | 2.022319000 | 1.312167000 |
| C | -0.882337000 | 2.460292000 | -0.147943000 |
| N | 0.198863000 | -0.417347000 | 1.036760000 |
| O | 4.265313000 | -0.296552000 | -0.788741000 |
| O | -1.481337000 | 3.535141000 | -0.324758000 |
| O | -3.600349000 | -2.045633000 | -0.686000000 |
| C | 2.774230000 | -0.744655000 | 1.011598000 |
| H | 0.520894000 | 0.865472000 | 2.688905000 |
| H | 1.353150000 | 1.318539000 | 1.204912000 |
| H | -1.621108000 | 1.766324000 | 1.744978000 |
| H | -0.334376000 | 2.918272000 | 1.853647000 |
| H | 1.381987000 | -1.451592000 | 2.470693000 |
| H | 3.045555000 | 0.117761000 | 1.627886000 |
| H | 3.488987000 | -1.524246000 | 1.287140000 |
| H | 1.221675000 | -2.237955000 | 0.902424000 |
| H | -3.127383000 | -1.271434000 | 1.635686000 |
| H | -2.597249000 | 0.237939000 | 0.927512000 |
| H | -0.817305000 | -2.168120000 | 1.579055000 |
| H | -1.059818000 | -0.830620000 | 2.689300000 |

NTP Cr(III)

ΔG = -1901.030028

| | | | |
|---|---|---|---|
| Cr | 0.083660000 | -0.253277000 | -1.035576000 |
| O | -0.427837000 | 1.546705000 | -0.969278000 |

| | | | |
|---|---|---|---|
| O | 1.981465000 | 0.009005000 | -1.184346000 |
| O | -1.661344000 | -0.987828000 | -1.286550000 |
| C | -1.041004000 | -1.178134000 | 1.555575000 |
| C | -2.412623000 | -0.797872000 | 0.990276000 |
| C | -2.660708000 | -1.187030000 | -0.463378000 |
| C | 1.352195000 | -1.443769000 | 1.240127000 |
| C | 3.017885000 | -0.362087000 | -0.478138000 |
| C | 0.474892000 | 0.823963000 | 1.659498000 |
| C | -0.521411000 | 1.953887000 | 1.427344000 |
| C | -0.712456000 | 2.407737000 | -0.016851000 |
| N | 0.190688000 | -0.495943000 | 0.986800000 |
| O | 4.167797000 | -0.236136000 | -0.875876000 |
| O | -1.129302000 | 3.526293000 | -0.270410000 |
| O | -3.738490000 | -1.634942000 | -0.826517000 |
| C | 2.753897000 | -0.929803000 | 0.917472000 |
| H | 0.557782000 | 0.628259000 | 2.732734000 |
| H | 1.449524000 | 1.156747000 | 1.311365000 |
| H | -1.505139000 | 1.736824000 | 1.847893000 |
| H | -0.156217000 | 2.818955000 | 1.985154000 |
| H | 1.317664000 | -1.723659000 | 2.297169000 |
| H | 3.078911000 | -0.172870000 | 1.635995000 |
| H | 3.436938000 | -1.770613000 | 1.058514000 |
| H | 1.142354000 | -2.347710000 | 0.664434000 |
| H | -3.158332000 | -1.304903000 | 1.604777000 |
| H | -2.621581000 | 0.269120000 | 1.080948000 |
| H | -0.894385000 | -2.247872000 | 1.405763000 |
| H | -1.032498000 | -0.998167000 | 2.633751000 |

NTP Cu(II)

ΔG = -2497.136548

| | | | |
|---|---|---|---|
| Cu | 0.174503000 | -0.268772000 | -0.885018000 |
| O | -0.571261000 | 1.675956000 | -1.207919000 |
| O | 2.031598000 | -0.054157000 | -1.214781000 |
| O | -1.342626000 | -1.357823000 | -1.211962000 |
| C | -0.958552000 | -0.974581000 | 1.755840000 |
| C | -2.278824000 | -0.956269000 | 0.971183000 |
| C | -2.267729000 | -1.694875000 | -0.368297000 |
| C | 1.466251000 | -0.927948000 | 1.586116000 |
| C | 3.040816000 | -0.231766000 | -0.421853000 |
| C | 0.245571000 | 1.205027000 | 1.557470000 |
| C | -0.933860000 | 2.096499000 | 1.165411000 |
| C | -1.204839000 | 2.359452000 | -0.333792000 |
| N | 0.202475000 | -0.239844000 | 1.158855000 |
| O | 4.205284000 | -0.270245000 | -0.825687000 |
| O | -2.035162000 | 3.248991000 | -0.595115000 |
| O | -3.129969000 | -2.538583000 | -0.621035000 |
| C | 2.785305000 | -0.332597000 | 1.088019000 |
| H | 0.366336000 | 1.243129000 | 2.648242000 |
| H | 1.145823000 | 1.627301000 | 1.115345000 |
| H | -1.867005000 | 1.772045000 | 1.631044000 |
| H | -0.728975000 | 3.074437000 | 1.611529000 |
| H | 1.495496000 | -0.943575000 | 2.683397000 |
| H | 2.949333000 | 0.667599000 | 1.500145000 |
| H | 3.584469000 | -0.949432000 | 1.504264000 |
| H | 1.385396000 | -1.962955000 | 1.249448000 |
| H | -3.035861000 | -1.421583000 | 1.604543000 |
| H | -2.614107000 | 0.063643000 | 0.776314000 |
| H | -0.649750000 | -2.013450000 | 1.880360000 |
| H | -1.136830000 | -0.574624000 | 2.760304000 |

NTP Fe(II)

ΔG = -2120.369928

| | | | |
|---|---|---|---|
| Fe | 0.139783000 | -0.236832000 | -0.915757000 |
| O | -0.642763000 | 1.597003000 | -1.247352000 |
| O | 2.079364000 | -0.326023000 | -1.216684000 |
| O | -1.265793000 | -1.601464000 | -1.122739000 |
| C | -0.999256000 | -0.831396000 | 1.721917000 |
| C | -2.304697000 | -0.900537000 | 0.920543000 |
| C | -2.246535000 | -1.811329000 | -0.308664000 |
| C | 1.413223000 | -0.870087000 | 1.552396000 |
| C | 3.075508000 | -0.325298000 | -0.397169000 |
| C | 0.262092000 | 1.295180000 | 1.471080000 |
| C | -0.918214000 | 2.185156000 | 1.089852000 |
| C | -1.177231000 | 2.413304000 | -0.409067000 |
| N | 0.179707000 | -0.149542000 | 1.066489000 |
| O | 4.257901000 | -0.301224000 | -0.757141000 |
| O | -1.895478000 | 3.369596000 | -0.732525000 |
| O | -3.119583000 | -2.667781000 | -0.488211000 |
| C | 2.766978000 | -0.323360000 | 1.104092000 |
| H | 0.399644000 | 1.333795000 | 2.558653000 |
| H | 1.161307000 | 1.705015000 | 1.013454000 |
| H | -1.850668000 | 1.843419000 | 1.546931000 |
| H | -0.730141000 | 3.167185000 | 1.531925000 |
| H | 1.390153000 | -0.876482000 | 2.648818000 |
| H | 2.934477000 | 0.693819000 | 1.469733000 |
| H | 3.531758000 | -0.931658000 | 1.593193000 |
| H | 1.312740000 | -1.904571000 | 1.218859000 |
| H | -3.078396000 | -1.275218000 | 1.593327000 |
| H | -2.628233000 | 0.087722000 | 0.586459000 |
| H | -0.690243000 | -1.852169000 | 1.949910000 |
| H | -1.185111000 | -0.337087000 | 2.680346000 |

NTP Fe(III)

ΔG = -2120.186371

| | | | |
|---|---|---|---|
| Fe | 0.074797000 | -0.219388000 | -0.903351000 |
| O | -0.408047000 | 1.541614000 | -1.040971000 |

| | | | |
|---|---|---|---|
| O | 1.899372000 | -0.100500000 | -1.192728000 |
| O | -1.549276000 | -1.013213000 | -1.272334000 |
| C | -1.092628000 | -1.033345000 | 1.637272000 |
| C | -2.427237000 | -0.737377000 | 0.950128000 |
| C | -2.547365000 | -1.294795000 | -0.460663000 |
| C | 1.297283000 | -1.366279000 | 1.345808000 |
| C | 2.941033000 | -0.449962000 | -0.466524000 |
| C | 0.456844000 | 0.936571000 | 1.649410000 |
| C | -0.540984000 | 2.046815000 | 1.347328000 |
| C | -0.691808000 | 2.442588000 | -0.117078000 |
| N | 0.159434000 | -0.411175000 | 1.045593000 |
| O | 4.075790000 | -0.391944000 | -0.910930000 |
| O | -1.080767000 | 3.553392000 | -0.432018000 |
| O | -3.519850000 | -1.931404000 | -0.829763000 |
| C | 2.698207000 | -0.896137000 | 0.971881000 |
| H | 0.532619000 | 0.799100000 | 2.731946000 |
| H | 1.434837000 | 1.244745000 | 1.288196000 |
| H | -1.535681000 | 1.831217000 | 1.743958000 |
| H | -0.204993000 | 2.937794000 | 1.881459000 |
| H | 1.269348000 | -1.583182000 | 2.417468000 |
| H | 3.040439000 | -0.085943000 | 1.620976000 |
| H | 3.377747000 | -1.728070000 | 1.169312000 |
| H | 1.059824000 | -2.293920000 | 0.822470000 |
| H | -3.209244000 | -1.189403000 | 1.561618000 |
| H | -2.645735000 | 0.330888000 | 0.904189000 |
| H | -0.935329000 | -2.111421000 | 1.628074000 |
| H | -1.142435000 | -0.721526000 | 2.682903000 |

NTP K(I)

ΔG = -1456.764343

| | | | |
|---|---|---|---|
| K | 0.139517000 | -0.235488000 | -1.754900000 |
| O | -0.696003000 | 2.178144000 | -1.267036000 |
| O | 2.585406000 | -0.743410000 | -1.107014000 |
| O | -1.643897000 | -2.150550000 | -1.422458000 |
| C | -1.052906000 | -0.817342000 | 1.639855000 |
| C | -2.216574000 | -1.085500000 | 0.661446000 |
| C | -1.989091000 | -2.352739000 | -0.221643000 |
| C | 1.354406000 | -0.674229000 | 1.567660000 |
| C | 3.302633000 | -0.192207000 | -0.228193000 |
| C | 0.040059000 | 1.375030000 | 1.438958000 |
| C | -1.234066000 | 2.161075000 | 1.094381000 |
| C | -1.466471000 | 2.634829000 | -0.378104000 |
| N | 0.094315000 | -0.056620000 | 1.089749000 |
| O | 4.470023000 | 0.242767000 | -0.351707000 |
| O | -2.413052000 | 3.440981000 | -0.522613000 |
| O | -2.160745000 | -3.454229000 | 0.349675000 |
| C | 2.700597000 | -0.026134000 | 1.205543000 |
| H | 0.209223000 | 1.477238000 | 2.535212000 |
| H | 0.874579000 | 1.869742000 | 0.940341000 |
| H | -2.135135000 | 1.628721000 | 1.412065000 |
| H | -1.218013000 | 3.075712000 | 1.699457000 |
| H | 1.315857000 | -0.729008000 | 2.678338000 |
| H | 2.712248000 | 1.042149000 | 1.439046000 |
| H | 3.444698000 | -0.464737000 | 1.882076000 |
| H | 1.359865000 | -1.703383000 | 1.198914000 |
| H | -3.115945000 | -1.270465000 | 1.260531000 |
| H | -2.401616000 | -0.210036000 | 0.036226000 |
| H | -0.693891000 | -1.791853000 | 1.981788000 |
| H | -1.428284000 | -0.299795000 | 2.540671000 |

NTP Li(I)

ΔG = -864.376645

| | | | |
|---|---|---|---|
| Li | 0.155086000 | -0.144506000 | -1.073810000 |
| O | -0.625312000 | 1.587054000 | -1.405866000 |
| O | 2.031409000 | -0.355924000 | -1.372510000 |
| O | -0.924555000 | -1.719111000 | -1.368221000 |
| C | -1.004431000 | -0.859469000 | 1.578798000 |
| C | -2.162696000 | -1.144919000 | 0.601386000 |
| C | -1.796076000 | -2.119063000 | -0.534252000 |
| C | 1.409852000 | -0.669355000 | 1.482006000 |
| C | 3.005398000 | -0.198624000 | -0.576851000 |
| C | 0.047256000 | 1.352614000 | 1.398754000 |
| C | -1.178925000 | 2.116936000 | 0.891741000 |
| C | -1.338152000 | 2.297385000 | -0.634753000 |
| N | 0.128948000 | -0.074976000 | 1.027921000 |
| O | 4.210658000 | -0.116558000 | -0.915887000 |
| O | -2.185336000 | 3.144881000 | -1.006201000 |
| O | -2.371816000 | -3.233152000 | -0.567245000 |
| C | 2.708115000 | -0.064961000 | 0.931904000 |
| H | 0.083895000 | 1.453095000 | 2.499038000 |
| H | 0.935479000 | 1.850232000 | 1.007761000 |
| H | -2.106379000 | 1.675868000 | 1.270188000 |
| H | -1.142900000 | 3.119189000 | 1.329822000 |
| H | 1.467666000 | -0.629072000 | 2.584709000 |
| H | 2.788550000 | 0.998122000 | 1.181225000 |
| H | 3.533104000 | -0.543316000 | 1.467445000 |
| H | 1.380937000 | -1.727314000 | 1.208713000 |
| H | -2.987429000 | -1.570259000 | 1.178478000 |
| H | -2.518084000 | -0.213394000 | 0.154673000 |
| H | -0.617610000 | -1.819804000 | 1.929861000 |
| H | -1.407559000 | -0.355780000 | 2.469120000 |

NTP Mg(II)

ΔG = -1056.867946

| | | | |
|---|---|---|---|
| Mg | 0.183655000 | -0.150163000 | -1.142281000 |
| O | -0.945811000 | 1.443574000 | -1.390774000 |

| | | | |
|---|---|---|---|
| O | 2.134047000 | −0.278422000 | −1.287546000 |
| O | −0.776941000 | −1.875701000 | −1.189574000 |
| C | −0.909509000 | −0.867416000 | 1.674093000 |
| C | −2.066573000 | −1.307131000 | 0.756453000 |
| C | −1.661744000 | −2.292780000 | −0.348793000 |
| C | 1.473208000 | −0.395030000 | 1.587538000 |
| C | 3.090073000 | −0.045466000 | −0.455738000 |
| C | −0.114347000 | 1.453830000 | 1.407899000 |
| C | −1.424234000 | 2.066377000 | 0.904066000 |
| C | −1.605562000 | 2.229943000 | −0.614341000 |
| N | 0.132839000 | 0.021976000 | 1.061433000 |
| O | 4.288573000 | −0.053600000 | −0.763640000 |
| O | −2.395469000 | 3.094350000 | −1.015650000 |
| O | −2.187651000 | −3.412087000 | −0.394744000 |
| C | 2.710928000 | 0.291725000 | 0.994630000 |
| H | −0.081348000 | 1.561031000 | 2.501644000 |
| H | 0.712459000 | 2.039640000 | 1.004561000 |
| H | −2.290871000 | 1.513351000 | 1.279315000 |
| H | −1.507300000 | 3.062028000 | 1.346237000 |
| H | 1.488179000 | −0.241432000 | 2.675876000 |
| H | 2.637752000 | 1.381746000 | 1.064246000 |
| H | 3.560306000 | 0.017854000 | 1.624025000 |
| H | 1.557722000 | −1.470136000 | 1.416301000 |
| H | −2.819708000 | −1.784532000 | 1.385665000 |
| H | −2.542133000 | −0.443265000 | 0.284530000 |
| H | −0.409089000 | −1.766378000 | 2.038175000 |
| H | −1.327122000 | −0.371015000 | 2.557338000 |

NTP Mn(II)

ΔG = −2007.66298

| | | | |
|---|---|---|---|
| Mn | 0.232416000 | −0.087413000 | −0.952540000 |
| O | −1.354600000 | 1.010451000 | −1.378613000 |
| O | 2.173826000 | −0.444984000 | −1.088776000 |
| O | −0.521679000 | −2.029065000 | −0.910889000 |
| C | −0.693786000 | −0.737520000 | 1.812912000 |
| C | −1.827064000 | −1.401948000 | 1.010174000 |
| C | −1.375860000 | −2.432409000 | −0.038128000 |
| C | 1.559480000 | 0.186402000 | 1.683062000 |
| C | 3.102092000 | 0.117457000 | −0.386038000 |
| C | −0.343490000 | 1.665784000 | 1.328094000 |
| C | −1.754627000 | 1.968188000 | 0.823478000 |
| C | −2.036747000 | 1.858439000 | −0.682215000 |
| N | 0.174481000 | 0.276313000 | 1.104248000 |
| O | 4.301070000 | 0.085435000 | −0.686810000 |
| O | −2.935060000 | 2.560878000 | −1.160243000 |
| O | −1.856477000 | −3.575718000 | −0.007421000 |
| C | 2.663496000 | 0.873762000 | 0.875030000 |
| H | −0.315034000 | 1.869406000 | 2.406732000 |
| H | 0.356646000 | 2.354210000 | 0.853156000 |
| H | −2.494624000 | 1.347350000 | 1.337636000 |
| H | −1.985527000 | 2.994194000 | 1.118818000 |
| H | 1.541483000 | 0.595807000 | 2.700701000 |
| H | 2.377695000 | 1.888497000 | 0.580068000 |
| H | 3.537935000 | 0.982633000 | 1.519089000 |
| H | 1.802905000 | −0.872914000 | 1.765345000 |
| H | −2.469943000 | −1.909356000 | 1.731929000 |
| H | −2.444134000 | −0.660763000 | 0.498978000 |
| H | −0.035455000 | −1.526875000 | 2.176209000 |
| H | −1.121318000 | −0.255962000 | 2.698908000 |

NTP Na(I)

ΔG = −1019.130644

| | | | |
|---|---|---|---|
| Na | 0.174696000 | −0.120978000 | −1.500385000 |
| O | −0.296427000 | 2.110850000 | −1.270186000 |
| O | 2.290995000 | −0.913451000 | −1.257120000 |
| O | −1.512670000 | −1.667848000 | −1.570543000 |
| C | −1.140258000 | −0.777085000 | 1.576046000 |
| C | −2.302343000 | −0.918154000 | 0.572862000 |
| C | −2.042992000 | −2.021071000 | −0.473342000 |
| C | 1.268148000 | −0.928007000 | 1.475872000 |
| C | 3.052734000 | −0.363489000 | −0.409441000 |
| C | 0.206591000 | 1.271179000 | 1.453865000 |
| C | −0.949656000 | 2.192991000 | 1.061150000 |
| C | −1.163700000 | 2.506613000 | −0.438077000 |
| N | 0.088363000 | −0.141410000 | 1.037960000 |
| O | 4.143263000 | 0.206456000 | −0.665817000 |
| O | −2.196853000 | 3.167057000 | −0.715397000 |
| O | −2.352898000 | −3.197910000 | −0.158627000 |
| C | 2.655224000 | −0.412536000 | 1.083563000 |
| H | 0.325389000 | 1.326985000 | 2.552811000 |
| H | 1.119025000 | 1.675004000 | 1.014765000 |
| H | −1.901231000 | 1.846936000 | 1.472280000 |
| H | −0.766691000 | 3.158798000 | 1.547488000 |
| H | 1.259777000 | −1.020779000 | 2.577766000 |
| H | 2.860450000 | 0.567608000 | 1.519900000 |
| H | 3.378333000 | −1.086599000 | 1.559274000 |
| H | 1.143314000 | −1.936096000 | 1.073081000 |
| H | −3.197771000 | −1.186276000 | 1.141605000 |
| H | −2.491225000 | 0.033790000 | 0.073577000 |
| H | −0.886241000 | −1.778802000 | 1.932930000 |
| H | −1.489108000 | −0.223822000 | 2.459569000 |

NTP Ni(II)

ΔG = −2364.937718

| | | | |
|---|---|---|---|
| Ni | 0.255064000 | −0.425342000 | −0.887668000 |
| O | −0.601524000 | 1.814438000 | −1.193758000 |

| | | | |
|---|---|---|---|
| O | 2.005614000 | 0.061756000 | -1.183987000 |
| O | -1.359101000 | -1.250926000 | -1.161383000 |
| C | -0.848023000 | -1.045089000 | 1.700462000 |
| C | -2.230633000 | -0.944565000 | 1.055873000 |
| C | -2.345023000 | -1.501614000 | -0.358678000 |
| C | 1.557362000 | -0.989379000 | 1.492403000 |
| C | 3.062222000 | -0.067332000 | -0.444672000 |
| C | 0.345338000 | 1.147940000 | 1.443626000 |
| C | -0.880835000 | 2.022744000 | 1.196877000 |
| C | -1.353684000 | 2.225744000 | -0.263676000 |
| N | 0.286982000 | -0.307866000 | 1.042874000 |
| O | 4.203492000 | 0.078238000 | -0.884925000 |
| O | -2.449540000 | 2.818655000 | -0.402948000 |
| O | -3.349670000 | -2.122292000 | -0.708988000 |
| C | 2.866468000 | -0.344654000 | 1.047143000 |
| H | 0.582195000 | 1.167788000 | 2.514572000 |
| H | 1.185641000 | 1.580015000 | 0.906793000 |
| H | -1.730112000 | 1.730579000 | 1.815874000 |
| H | -0.600400000 | 3.017966000 | 1.561623000 |
| H | 1.544777000 | -1.029096000 | 2.587922000 |
| H | 3.038547000 | 0.602803000 | 1.566023000 |
| H | 3.677511000 | -1.002799000 | 1.366378000 |
| H | 1.503976000 | -2.014394000 | 1.124528000 |
| H | -2.924890000 | -1.496662000 | 1.692383000 |
| H | -2.595703000 | 0.082817000 | 1.023087000 |
| H | -0.558570000 | -2.096383000 | 1.728870000 |
| H | -0.906951000 | -0.699715000 | 2.738284000 |

NTP V(II)

ΔG = -1800.685837

| | | | |
|---|---|---|---|
| V | 0.111201000 | -0.304990000 | -1.129177000 |
| O | -0.540206000 | 1.626673000 | -1.131753000 |
| O | 2.148155000 | -0.153752000 | -1.235374000 |
| O | -1.699600000 | -1.248326000 | -1.278377000 |
| C | -1.028394000 | -0.961947000 | 1.654272000 |
| C | -2.386435000 | -0.734835000 | 0.966219000 |
| C | -2.589067000 | -1.458981000 | -0.370086000 |
| C | 1.359138000 | -1.206980000 | 1.416889000 |
| C | 3.108364000 | -0.420722000 | -0.420739000 |
| C | 0.426748000 | 1.047289000 | 1.556470000 |
| C | -0.629492000 | 2.109179000 | 1.247806000 |
| C | -0.873813000 | 2.474931000 | -0.225228000 |
| N | 0.191808000 | -0.336250000 | 1.039331000 |
| O | 4.305948000 | -0.416406000 | -0.740853000 |
| O | -1.399179000 | 3.572791000 | -0.468790000 |
| O | -3.584863000 | -2.179784000 | -0.529726000 |
| C | 2.764028000 | -0.719545000 | 1.047738000 |
| H | 0.555713000 | 0.994259000 | 2.646947000 |
| H | 1.371438000 | 1.388525000 | 1.135486000 |
| H | -1.595338000 | 1.863644000 | 1.696970000 |
| H | -0.309333000 | 3.025439000 | 1.751025000 |
| H | 1.340925000 | -1.363515000 | 2.504616000 |
| H | 3.032679000 | 0.172839000 | 1.621015000 |
| H | 3.458726000 | -1.492315000 | 1.387018000 |
| H | 1.178584000 | -2.179080000 | 0.951449000 |
| H | -3.154182000 | -1.084993000 | 1.659136000 |
| H | -2.578786000 | 0.325638000 | 0.795662000 |
| H | -0.849521000 | -2.038355000 | 1.689888000 |
| H | -1.099690000 | -0.623455000 | 2.695462000 |

NTP V(III)

ΔG = -1800.530723

| | | | |
|---|---|---|---|
| V | 0.097783000 | -0.011387000 | -1.029691000 |
| O | -0.809598000 | 1.577872000 | -1.181670000 |
| O | 1.989474000 | 0.267457000 | -1.106606000 |
| O | -0.071338000 | -1.864169000 | -1.053846000 |
| C | -1.098871000 | -1.147210000 | 1.607860000 |
| C | -1.842191000 | -1.970812000 | 0.547648000 |
| C | -0.927946000 | -2.675690000 | -0.437257000 |
| C | 1.208517000 | -0.353374000 | 1.692233000 |
| C | 2.870566000 | 0.482996000 | -0.151196000 |
| C | -0.626509000 | 1.278331000 | 1.597424000 |
| C | -1.908434000 | 1.794797000 | 0.946214000 |
| C | -1.754802000 | 2.231205000 | -0.501982000 |
| N | -0.152733000 | -0.063335000 | 1.102453000 |
| O | 4.056497000 | 0.649675000 | -0.395064000 |
| O | -2.430714000 | 3.096443000 | -1.012766000 |
| O | -0.965401000 | -3.867395000 | -0.664328000 |
| C | 2.360133000 | 0.567701000 | 1.285753000 |
| H | -0.772988000 | 1.204932000 | 2.679251000 |
| H | 0.168481000 | 2.001378000 | 1.427547000 |
| H | -2.710412000 | 1.049711000 | 0.980797000 |
| H | -2.268575000 | 2.653926000 | 1.514184000 |
| H | 1.114070000 | -0.323761000 | 2.783619000 |
| H | 2.129703000 | 1.618776000 | 1.483173000 |
| H | 3.203047000 | 0.328776000 | 1.936679000 |
| H | 1.453781000 | -1.380236000 | 1.417937000 |
| H | -2.440484000 | -2.720116000 | 1.067431000 |
| H | -2.535956000 | -1.346210000 | -0.024765000 |
| H | -0.516845000 | -1.824823000 | 2.232436000 |
| H | -1.837849000 | -0.683847000 | 2.261484000 |

NTP Zn(II)

ΔG = -2635.899421

| | | | |
|---|---|---|---|
| Zn | 0.018624000 | -0.248619000 | -0.860741000 |
| O | 0.118982000 | 1.673343000 | -1.291553000 |

| | | | |
|---|---|---|---|
| O | 1.799817000 | -1.074419000 | -1.102640000 |
| O | -3.488177000 | -1.656507000 | -0.997632000 |
| C | -1.121821000 | -0.906959000 | 1.860205000 |
| C | -2.434813000 | -0.893006000 | 1.068373000 |
| C | -2.402993000 | -1.734983000 | -0.192180000 |
| C | 1.300134000 | -0.617462000 | 1.803897000 |
| C | 2.799072000 | -0.921762000 | -0.295674000 |
| C | -0.118630000 | 1.364314000 | 1.702356000 |
| C | -0.938218000 | 2.300416000 | 0.799427000 |
| C | -0.320132000 | 2.647151000 | -0.560164000 |
| N | -0.003193000 | -0.075571000 | 1.295633000 |
| O | 3.936488000 | -1.338659000 | -0.541676000 |
| O | -0.275743000 | 3.829271000 | -0.918444000 |
| O | -1.375335000 | -1.627476000 | -1.068211000 |
| C | 2.545871000 | -0.173835000 | 1.020860000 |
| H | -0.535502000 | 1.400532000 | 2.715474000 |
| H | 0.891948000 | 1.768133000 | 1.770446000 |
| H | -1.938461000 | 1.899945000 | 0.617748000 |
| H | -1.075741000 | 3.234862000 | 1.346180000 |
| H | 1.415682000 | -0.330957000 | 2.856607000 |
| H | 2.508568000 | 0.897137000 | 0.794435000 |
| H | 3.418189000 | -0.321132000 | 1.658825000 |
| H | 1.235683000 | -1.705719000 | 1.775526000 |
| H | -3.218083000 | -1.288239000 | 1.722693000 |
| H | -2.744456000 | 0.131883000 | 0.820036000 |
| H | -0.766521000 | -1.938156000 | 1.900892000 |
| H | -1.306756000 | -0.588404000 | 2.892561000 |

10-HBQS

ΔG = -1482.392923

| | | | |
|---|---|---|---|
| C | -3.707604000 | -3.020999000 | 0.225199000 |
| C | -2.395583000 | -3.394560000 | 0.025846000 |
| C | -1.403337000 | -2.410953000 | -0.138102000 |
| C | -1.779410000 | -1.028216000 | -0.099917000 |
| C | -3.989918000 | -1.648916000 | 0.258491000 |
| C | -0.037478000 | -2.778726000 | -0.339526000 |
| C | -0.761854000 | -0.001981000 | -0.276383000 |
| C | 0.596028000 | -0.432559000 | -0.451047000 |
| C | 0.920055000 | -1.831531000 | -0.485746000 |
| C | 1.610810000 | 0.529143000 | -0.617527000 |
| C | 1.341477000 | 1.884927000 | -0.657936000 |
| C | 0.038202000 | 2.320525000 | -0.493947000 |
| C | -1.061841000 | 1.433537000 | -0.296794000 |
| H | 0.217669000 | -3.832770000 | -0.373256000 |
| H | -4.498108000 | -3.749695000 | 0.355352000 |
| H | -2.110403000 | -4.441075000 | -0.006024000 |
| H | -5.010380000 | -1.310108000 | 0.420345000 |
| H | 1.950587000 | -2.120730000 | -0.639306000 |
| H | 2.147699000 | 2.591604000 | -0.815086000 |
| H | -0.185226000 | 3.381236000 | -0.523226000 |
| N | -3.083432000 | -0.693120000 | 0.105360000 |
| O | -2.242385000 | 1.904669000 | -0.158972000 |
| O | -4.990339000 | 1.504963000 | 0.540678000 |
| H | -4.149540000 | 1.039060000 | 0.342015000 |
| H | -4.743297000 | 2.440492000 | 0.472456000 |
| O | -3.626616000 | 4.112723000 | 0.116180000 |
| H | -2.945609000 | 3.393169000 | 0.005456000 |
| H | -3.329685000 | 4.646231000 | 0.860343000 |
| O | 2.935658000 | 0.067988000 | -0.873046000 |
| S | 4.077038000 | 0.173047000 | 0.278916000 |
| O | 3.511329000 | -0.777193000 | 1.461808000 |
| O | 4.178652000 | 1.532546000 | 0.774702000 |
| O | 5.218457000 | -0.519017000 | -0.269392000 |
| H | 2.912376000 | -0.285906000 | 2.049446000 |

10-HBQS Al(III)

ΔG = -1724.498528

| | | | |
|---|---|---|---|
| C | -3.494051000 | 2.883755000 | 0.174152000 |
| C | -2.182145000 | 3.271695000 | -0.010707000 |
| C | -1.162677000 | 2.315947000 | -0.156005000 |
| C | -1.483848000 | 0.929831000 | -0.112836000 |
| C | -3.769645000 | 1.529803000 | 0.213436000 |
| C | 0.189321000 | 2.735509000 | -0.350338000 |
| C | -0.431463000 | -0.057411000 | -0.264795000 |
| C | 0.908995000 | 0.423988000 | -0.444856000 |
| C | 1.182558000 | 1.827533000 | -0.489986000 |
| C | 1.952866000 | -0.509604000 | -0.592170000 |
| C | 1.734042000 | -1.868669000 | -0.594383000 |
| C | 0.437960000 | -2.345112000 | -0.416970000 |
| C | -0.628126000 | -1.474163000 | -0.251695000 |
| H | 0.396736000 | 3.797747000 | -0.386095000 |
| H | -4.295663000 | 3.599491000 | 0.288089000 |
| H | -1.920611000 | 4.322613000 | -0.046150000 |
| H | -4.784754000 | 1.180950000 | 0.359955000 |
| H | 2.200351000 | 2.153333000 | -0.649284000 |
| H | 2.557860000 | -2.555986000 | -0.733056000 |
| H | 0.234984000 | -3.407945000 | -0.411074000 |
| N | -2.814245000 | 0.582846000 | 0.078236000 |
| O | -1.845392000 | -2.035688000 | -0.090278000 |
| H | -4.960022000 | -2.234496000 | 1.794545000 |
| H | -4.097872000 | -1.137509000 | 2.536243000 |
| H | -5.494394000 | -1.643315000 | -1.142248000 |
| H | -4.265230000 | -2.282158000 | -1.908834000 |
| O | -4.289242000 | -1.540997000 | 1.672182000 |
| O | -4.535199000 | -1.796238000 | -1.110841000 |

| | | | |
|---|---|---|---|
| Al | -3.323074000 | -1.221353000 | 0.142532000 |
| O | 3.247370000 | -0.012873000 | -0.841663000 |
| S | 4.368899000 | 0.013178000 | 0.356497000 |
| O | 4.801791000 | -1.533346000 | 0.475019000 |
| O | 3.735307000 | 0.322617000 | 1.615641000 |
| O | 5.449467000 | 0.797530000 | -0.191028000 |
| H | 5.390356000 | -1.792560000 | -0.255956000 |

10-HBQS Be(II)

ΔG = -1497.006004

| | | | |
|---|---|---|---|
| C | -3.816219000 | 2.571772000 | -0.191981000 |
| C | -2.534953000 | 3.061799000 | -0.030256000 |
| C | -1.454690000 | 2.177655000 | 0.123887000 |
| C | -1.703395000 | 0.776055000 | 0.106176000 |
| C | -3.995626000 | 1.193513000 | -0.205978000 |
| C | -0.118417000 | 2.657467000 | 0.303409000 |
| C | -0.612170000 | -0.152596000 | 0.283090000 |
| C | 0.705296000 | 0.377637000 | 0.451914000 |
| C | 0.916589000 | 1.796111000 | 0.458242000 |
| C | 1.769205000 | -0.523285000 | 0.636913000 |
| C | 1.576200000 | -1.889183000 | 0.694019000 |
| C | 0.295835000 | -2.407479000 | 0.529424000 |
| C | -0.807179000 | -1.572822000 | 0.317438000 |
| H | 0.046431000 | 3.728386000 | 0.317217000 |
| H | -4.668633000 | 3.227093000 | -0.306926000 |
| H | -2.348964000 | 4.129699000 | -0.017114000 |
| H | -4.983365000 | 0.765559000 | -0.323996000 |
| H | 1.921020000 | 2.170073000 | 0.598674000 |
| H | 2.418411000 | -2.547475000 | 0.863874000 |
| H | 0.126968000 | -3.476389000 | 0.566887000 |
| N | -2.985336000 | 0.322908000 | -0.070659000 |
| O | -1.998550000 | -2.122855000 | 0.164983000 |
| H | -5.118700000 | -2.511901000 | 0.661298000 |
| H | -4.602955000 | -1.534639000 | 1.766805000 |
| H | -4.535248000 | -1.495383000 | -2.197182000 |
| H | -3.214527000 | -2.337778000 | -2.222513000 |
| O | -4.632453000 | -1.689682000 | 0.812633000 |
| O | -3.858040000 | -1.906859000 | -1.644263000 |
| Be | -3.281268000 | -1.346385000 | -0.145650000 |
| O | 3.053413000 | 0.019173000 | 0.879986000 |
| S | 4.219973000 | -0.061551000 | -0.258460000 |
| O | 3.626487000 | 0.857022000 | -1.447234000 |
| O | 4.364686000 | -1.422315000 | -0.733804000 |
| O | 5.319655000 | 0.674367000 | 0.310700000 |
| H | 3.137986000 | 0.322091000 | -2.096246000 |

10-HBQS B(III)

ΔG = -1506.824275

| | | | |
|---|---|---|---|
| C | -3.930161000 | 2.404009000 | 0.272396000 |
| C | -2.682138000 | 2.964672000 | 0.051720000 |
| C | -1.556659000 | 2.144644000 | -0.122511000 |
| C | -1.727032000 | 0.740170000 | -0.070303000 |
| C | -4.053698000 | 1.028196000 | 0.306859000 |
| C | -0.244355000 | 2.673901000 | -0.347989000 |
| C | -0.606559000 | -0.129008000 | -0.214654000 |
| C | 0.683420000 | 0.431808000 | -0.428802000 |
| C | 0.828210000 | 1.854619000 | -0.501557000 |
| C | 1.761565000 | -0.464453000 | -0.555434000 |
| C | 1.592921000 | -1.832933000 | -0.486739000 |
| C | 0.323102000 | -2.375076000 | -0.257517000 |
| C | -0.755058000 | -1.526196000 | -0.123594000 |
| H | -0.127999000 | 3.749284000 | -0.394059000 |
| H | -4.809466000 | 3.015258000 | 0.417049000 |
| H | -2.565252000 | 4.041213000 | 0.016418000 |
| H | -5.008323000 | 0.552422000 | 0.483174000 |
| H | 1.809928000 | 2.270614000 | -0.680529000 |
| H | 2.445373000 | -2.487950000 | -0.608219000 |
| H | 0.178467000 | -3.445186000 | -0.192767000 |
| N | -2.985876000 | 0.212973000 | 0.132481000 |
| O | -1.992528000 | -2.054456000 | 0.111007000 |
| H | -4.542283000 | -2.555943000 | 1.232351000 |
| H | -3.743865000 | -1.568276000 | 2.191893000 |
| H | -4.773503000 | -1.307892000 | -1.459976000 |
| H | -3.563284000 | -2.310839000 | -1.769099000 |
| O | -4.086274000 | -1.695759000 | 1.287949000 |
| O | -4.022742000 | -1.797662000 | -1.080954000 |
| B | -3.150938000 | -1.305724000 | 0.116782000 |
| O | 3.023322000 | 0.078941000 | -0.847624000 |
| S | 4.185232000 | 0.154841000 | 0.313805000 |
| O | 4.731989000 | -1.357367000 | 0.362264000 |
| O | 3.573136000 | 0.376983000 | 1.600908000 |
| O | 5.182963000 | 1.032927000 | -0.246432000 |
| H | 5.312045000 | -1.547323000 | -0.396578000 |

10-HBQS Ca(I)

ΔG = -2159.907469

| | | | |
|---|---|---|---|
| C | -2.907773000 | 3.487885000 | -0.293612000 |
| C | -1.561265000 | 3.719997000 | -0.124374000 |
| C | -0.682777000 | 2.641001000 | 0.072935000 |
| C | -1.198098000 | 1.304510000 | 0.102956000 |
| C | -3.336582000 | 2.160006000 | -0.277338000 |
| C | 0.715463000 | 2.885558000 | 0.237343000 |
| C | -0.279634000 | 0.196895000 | 0.342059000 |
| C | 1.118070000 | 0.505764000 | 0.477344000 |
| C | 1.580478000 | 1.862335000 | 0.421471000 |
| C | 2.038368000 | -0.534238000 | 0.704519000 |

| | | | |
|---|---|---|---|
| C | 1.644553000 | -1.848541000 | 0.855694000 |
| C | 0.300305000 | -2.161919000 | 0.741020000 |
| C | -0.694202000 | -1.190139000 | 0.476751000 |
| H | 1.066758000 | 3.911029000 | 0.209401000 |
| H | -3.618281000 | 4.289999000 | -0.444398000 |
| H | -1.160860000 | 4.727757000 | -0.140999000 |
| H | -4.393551000 | 1.955138000 | -0.422257000 |
| H | 2.636684000 | 2.055961000 | 0.543534000 |
| H | 2.380201000 | -2.616716000 | 1.059357000 |
| H | -0.032577000 | -3.186629000 | 0.855628000 |
| N | -2.539204000 | 1.105937000 | -0.095020000 |
| O | -1.923158000 | -1.583587000 | 0.382506000 |
| H | -4.185044000 | -2.181172000 | 2.442062000 |
| H | -5.663929000 | -1.947319000 | 2.055526000 |
| H | -4.105990000 | -2.655477000 | -3.008388000 |
| H | -2.722302000 | -2.976631000 | -2.401110000 |
| O | -4.758250000 | -1.855234000 | 1.738526000 |
| O | -3.518780000 | -2.448713000 | -2.272266000 |
| Ca | -3.883297000 | -1.023400000 | -0.358245000 |
| O | 3.402723000 | -0.186328000 | 0.901401000 |
| S | 4.506802000 | -0.489255000 | -0.256174000 |
| O | 4.070812000 | 0.504367000 | -1.456187000 |
| O | 4.399001000 | -1.860705000 | -0.713265000 |
| O | 5.737610000 | 0.045804000 | 0.270562000 |
| H | 3.429311000 | 0.079071000 | -2.050337000 |

10-HBQS Co(II)

ΔG = -2864.921469

| | | | |
|---|---|---|---|
| C | -2.927356000 | 3.196109000 | -0.428709000 |
| C | -1.620445000 | 3.495179000 | -0.112328000 |
| C | -0.703865000 | 2.457399000 | 0.117390000 |
| C | -1.135167000 | 1.100020000 | 0.017468000 |
| C | -3.309888000 | 1.858942000 | -0.442772000 |
| C | 0.655110000 | 2.755412000 | 0.451165000 |
| C | -0.155817000 | 0.040330000 | 0.134264000 |
| C | 1.200496000 | 0.395821000 | 0.436990000 |
| C | 1.564124000 | 1.768676000 | 0.628210000 |
| C | 2.166523000 | -0.622029000 | 0.523782000 |
| C | 1.857034000 | -1.952061000 | 0.320023000 |
| C | 0.554528000 | -2.301201000 | -0.006713000 |
| C | -0.464740000 | -1.339907000 | -0.105313000 |
| H | 0.938306000 | 3.795870000 | 0.559955000 |
| H | -3.661111000 | 3.964010000 | -0.632806000 |
| H | -1.283116000 | 4.522930000 | -0.043124000 |
| H | -4.338746000 | 1.589609000 | -0.625115000 |
| H | 2.585753000 | 2.005468000 | 0.889195000 |
| H | 2.624842000 | -2.709354000 | 0.410812000 |
| H | 0.290504000 | -3.336507000 | -0.181912000 |
| N | -2.468410000 | 0.842507000 | -0.200800000 |
| O | -1.662208000 | -1.767753000 | -0.437008000 |
| H | -5.862874000 | -0.603863000 | -0.132346000 |
| H | -5.366678000 | -0.296423000 | 1.304710000 |
| H | -4.678705000 | -3.152148000 | -0.285909000 |
| H | -3.145028000 | -3.366175000 | -0.101989000 |
| O | -5.113603000 | -0.259743000 | 0.372260000 |
| O | -3.896419000 | -2.817745000 | 0.172375000 |
| Co | -3.256067000 | -0.928253000 | -0.063037000 |
| O | 3.475969000 | -0.255915000 | 0.920902000 |
| S | 4.712994000 | -0.283854000 | -0.142909000 |
| O | 4.344126000 | 0.913544000 | -1.163028000 |
| O | 4.727172000 | -1.539043000 | -0.866292000 |
| O | 5.845023000 | 0.175784000 | 0.620448000 |
| H | 3.837936000 | 0.584386000 | -1.925328000 |

10-HBQS Co(III)

ΔG = -2864.674486

| | | | |
|---|---|---|---|
| C | -3.401812000 | 3.018408000 | -0.040533000 |
| C | -2.109710000 | 3.483313000 | -0.080369000 |
| C | -1.036773000 | 2.570316000 | -0.131039000 |
| C | -1.316653000 | 1.171878000 | -0.154778000 |
| C | -3.613275000 | 1.637519000 | -0.018237000 |
| C | 0.313215000 | 3.024162000 | -0.150776000 |
| C | -0.230362000 | 0.244063000 | -0.258507000 |
| C | 1.098758000 | 0.741406000 | -0.258416000 |
| C | 1.349062000 | 2.143903000 | -0.198587000 |
| C | 2.172327000 | -0.188149000 | -0.330096000 |
| C | 1.971370000 | -1.561572000 | -0.392443000 |
| C | 0.676897000 | -2.047522000 | -0.394817000 |
| C | -0.426057000 | -1.170611000 | -0.365534000 |
| H | 0.497264000 | 4.090933000 | -0.119300000 |
| H | -4.252516000 | 3.684361000 | -0.001937000 |
| H | -1.899772000 | 4.545899000 | -0.068648000 |
| H | -4.617550000 | 1.240897000 | 0.058814000 |
| H | 2.370842000 | 2.494731000 | -0.200351000 |
| H | 2.808190000 | -2.242655000 | -0.457543000 |
| H | 0.480594000 | -3.109376000 | -0.463352000 |
| N | -2.618589000 | 0.747487000 | -0.069425000 |
| O | -1.607935000 | -1.709396000 | -0.498125000 |
| H | -4.674168000 | -1.711865000 | 1.802330000 |
| H | -3.913369000 | -0.428594000 | 2.274906000 |
| H | -4.548056000 | -3.078769000 | -0.915945000 |
| H | -3.237264000 | -3.586206000 | -0.249419000 |
| O | -4.284165000 | -0.876688000 | 1.500393000 |
| O | -3.925240000 | -2.906140000 | -0.194067000 |
| Co | -3.194238000 | -1.065886000 | -0.091962000 |
| O | 3.423827000 | 0.385092000 | -0.396957000 |

| | | | |
|---|---|---|---|
| S | 4.769191000 | -0.220686000 | 0.364577000 |
| O | 5.174384000 | -1.443017000 | -0.588518000 |
| O | 4.374183000 | -0.817578000 | 1.613176000 |
| O | 5.716135000 | 0.856645000 | 0.255299000 |
| H | 5.601872000 | -1.135912000 | -1.408664000 |

10-HBQS Cr(II)

ΔG = -2526.630837

| | | | |
|---|---|---|---|
| C | -2.990500000 | -3.293850000 | 0.396345000 |
| C | -1.669231000 | -3.574759000 | 0.092320000 |
| C | -0.763342000 | -2.518008000 | -0.089648000 |
| C | -1.207992000 | -1.165914000 | 0.031068000 |
| C | -3.378323000 | -1.967960000 | 0.485363000 |
| C | 0.606846000 | -2.791516000 | -0.398876000 |
| C | -0.254091000 | -0.083885000 | -0.115754000 |
| C | 1.112913000 | -0.420961000 | -0.404336000 |
| C | 1.504110000 | -1.790941000 | -0.557671000 |
| C | 2.059386000 | 0.609919000 | -0.542729000 |
| C | 1.725905000 | 1.944152000 | -0.432171000 |
| C | 0.412006000 | 2.281528000 | -0.139155000 |
| C | -0.583534000 | 1.308823000 | 0.030223000 |
| H | 0.910379000 | -3.826777000 | -0.505168000 |
| H | -3.719683000 | -4.076497000 | 0.559517000 |
| H | -1.320347000 | -4.596215000 | -0.001202000 |
| H | -4.407309000 | -1.717791000 | 0.720875000 |
| H | 2.534280000 | -2.013734000 | -0.796704000 |
| H | 2.478784000 | 2.709374000 | -0.567858000 |
| H | 0.121545000 | 3.319585000 | -0.036309000 |
| N | -2.543325000 | -0.920324000 | 0.291569000 |
| O | -1.792389000 | 1.752253000 | 0.334068000 |
| H | -4.150456000 | 1.720006000 | -2.037767000 |
| H | -3.596526000 | 0.275530000 | -2.265735000 |
| H | -5.113045000 | 2.977489000 | 0.966770000 |
| H | -3.688974000 | 3.486344000 | 0.632792000 |
| O | -3.965296000 | 0.875613000 | -1.605357000 |
| O | -4.339050000 | 2.804516000 | 0.415187000 |
| Cr | -3.417958000 | 0.856565000 | 0.361580000 |
| O | 3.380137000 | 0.247025000 | -0.908626000 |
| S | 4.603661000 | 0.352757000 | 0.163820000 |
| O | 4.246212000 | -0.798696000 | 1.239343000 |
| O | 4.587039000 | 1.642252000 | 0.824338000 |
| O | 5.753010000 | -0.122470000 | -0.563570000 |
| H | 3.724805000 | -0.440424000 | 1.977848000 |

10-HBQS Cr(III)

ΔG = -2526.490067

| | | | |
|---|---|---|---|
| C | -3.308229000 | 3.131998000 | -0.013905000 |
| C | -2.003388000 | 3.546934000 | -0.066499000 |
| C | -0.966287000 | 2.589793000 | -0.113096000 |
| C | -1.292569000 | 1.198738000 | -0.112720000 |
| C | -3.562776000 | 1.756536000 | 0.019617000 |
| C | 0.388459000 | 3.013000000 | -0.158530000 |
| C | -0.220867000 | 0.239778000 | -0.211832000 |
| C | 1.116999000 | 0.716138000 | -0.253235000 |
| C | 1.403660000 | 2.107943000 | -0.214965000 |
| C | 2.193231000 | -0.220975000 | -0.346016000 |
| C | 1.989506000 | -1.594377000 | -0.429558000 |
| C | 0.697415000 | -2.066156000 | -0.408616000 |
| C | -0.421726000 | -1.190744000 | -0.295654000 |
| H | 0.598009000 | 4.075669000 | -0.146414000 |
| H | -4.134699000 | 3.828502000 | 0.017708000 |
| H | -1.748390000 | 4.599921000 | -0.073307000 |
| H | -4.580656000 | 1.399937000 | 0.097090000 |
| H | 2.432568000 | 2.434952000 | -0.243654000 |
| H | 2.823250000 | -2.276006000 | -0.524622000 |
| H | 0.487762000 | -3.125254000 | -0.481216000 |
| N | -2.612844000 | 0.819669000 | -0.020956000 |
| O | -1.577995000 | -1.749941000 | -0.283060000 |
| H | -6.091782000 | -0.862223000 | 0.181360000 |
| H | -5.559194000 | -0.795776000 | 1.644442000 |
| H | -4.764326000 | -3.509507000 | -0.281501000 |
| H | -3.237123000 | -3.785345000 | -0.457290000 |
| O | -5.305794000 | -0.691330000 | 0.717389000 |
| O | -3.882256000 | -3.165755000 | -0.092005000 |
| Cr | -3.334511000 | -1.150248000 | 0.081043000 |
| O | 3.446520000 | 0.345338000 | -0.421095000 |
| S | 4.782964000 | -0.256607000 | 0.362296000 |
| O | 5.195849000 | -1.488627000 | -0.574792000 |
| O | 4.371353000 | -0.840937000 | 1.611476000 |
| O | 5.734105000 | 0.817157000 | 0.254160000 |
| H | 5.630508000 | -1.189760000 | -1.394204000 |

10-HBQS Cu(II)

ΔG = -3122.634709

| | | | |
|---|---|---|---|
| C | -2.882206000 | 3.232100000 | -0.391777000 |
| C | -1.555570000 | 3.524776000 | -0.159418000 |
| C | -0.632664000 | 2.490176000 | 0.060278000 |
| C | -1.074397000 | 1.131372000 | 0.035284000 |
| C | -3.268004000 | 1.897557000 | -0.361397000 |
| C | 0.741531000 | 2.794615000 | 0.311162000 |
| C | -0.104787000 | 0.064464000 | 0.191370000 |
| C | 1.264033000 | 0.430595000 | 0.427507000 |
| C | 1.647752000 | 1.809340000 | 0.503763000 |
| C | 2.226457000 | -0.581967000 | 0.583413000 |
| C | 1.902324000 | -1.921863000 | 0.526571000 |
| C | 0.589045000 | -2.287413000 | 0.271602000 |

| | | | |
|---|---|---|---|
| C | -0.426030000 | -1.333207000 | 0.093254000 |
| H | 1.037439000 | 3.836379000 | 0.347815000 |
| H | -3.619408000 | 4.000984000 | -0.577991000 |
| H | -1.206910000 | 4.550981000 | -0.146146000 |
| H | -4.303251000 | 1.624498000 | -0.514476000 |
| H | 2.681806000 | 2.049896000 | 0.704551000 |
| H | 2.666043000 | -2.674451000 | 0.673178000 |
| H | 0.312114000 | -3.332047000 | 0.209460000 |
| N | -2.412420000 | 0.888887000 | -0.141810000 |
| O | -1.626831000 | -1.803704000 | -0.170678000 |
| H | -5.743523000 | -1.046145000 | 0.611480000 |
| H | -5.183010000 | 0.182045000 | 1.367948000 |
| H | -4.650054000 | -2.847525000 | -1.230813000 |
| H | -3.365822000 | -3.358749000 | -0.525860000 |
| O | -5.109052000 | -0.319249000 | 0.544057000 |
| O | -4.101253000 | -2.734058000 | -0.442500000 |
| Cu | -3.241938000 | -0.894314000 | -0.058232000 |
| O | 3.547279000 | -0.191263000 | 0.908454000 |
| S | 4.755563000 | -0.329031000 | -0.180776000 |
| O | 4.358671000 | 0.765555000 | -1.300099000 |
| O | 4.746295000 | -1.648363000 | -0.779064000 |
| O | 5.907909000 | 0.196692000 | 0.505146000 |
| H | 3.839664000 | 0.363390000 | -2.017534000 |

10-HBQS Fe(II)

ΔG = -2745.870268

| | | | |
|---|---|---|---|
| C | -2.936917000 | -3.193932000 | 0.444114000 |
| C | -1.622168000 | -3.496847000 | 0.162039000 |
| C | -0.700750000 | -2.466349000 | -0.083304000 |
| C | -1.135126000 | -1.106931000 | -0.039521000 |
| C | -3.316478000 | -1.857493000 | 0.416416000 |
| C | 0.665813000 | -2.775507000 | -0.369645000 |
| C | -0.159690000 | -0.046357000 | -0.186866000 |
| C | 1.204092000 | -0.414135000 | -0.440978000 |
| C | 1.576554000 | -1.792818000 | -0.558416000 |
| C | 2.170919000 | 0.599331000 | -0.567909000 |
| C | 1.854749000 | 1.937682000 | -0.457885000 |
| C | 0.543136000 | 2.300554000 | -0.184439000 |
| C | -0.475354000 | 1.346392000 | -0.045665000 |
| H | 0.953569000 | -3.818440000 | -0.430862000 |
| H | -3.671189000 | -3.957491000 | 0.661299000 |
| H | -1.281329000 | -4.525419000 | 0.135072000 |
| H | -4.344976000 | -1.583036000 | 0.607527000 |
| H | 2.605524000 | -2.036215000 | -0.781168000 |
| H | 2.622559000 | 2.690641000 | -0.578590000 |
| H | 0.271961000 | 3.343862000 | -0.084079000 |
| N | -2.469895000 | -0.847557000 | 0.157003000 |
| O | -1.684129000 | 1.806953000 | 0.211980000 |
| H | -5.770580000 | 0.985091000 | -0.789642000 |
| H | -5.178994000 | -0.372976000 | -1.238237000 |
| H | -4.793034000 | 3.019510000 | 0.876538000 |
| H | -3.328851000 | 3.403133000 | 0.488643000 |
| O | -5.119014000 | 0.309917000 | -0.556526000 |
| O | -4.057423000 | 2.795697000 | 0.291034000 |
| Fe | -3.300775000 | 0.940899000 | 0.087611000 |
| O | 3.487985000 | 0.213436000 | -0.913549000 |
| S | 4.703634000 | 0.308051000 | 0.171984000 |
| O | 4.319770000 | -0.835704000 | 1.245751000 |
| O | 4.693657000 | 1.599948000 | 0.827321000 |
| O | 5.852692000 | -0.183082000 | -0.544393000 |
| H | 3.805640000 | -0.466819000 | 1.984258000 |

10-HBQS Fe(III)

ΔG = -2745.638817

| | | | |
|---|---|---|---|
| C | -3.353222000 | -3.046169000 | 0.085854000 |
| C | -2.050224000 | -3.487959000 | 0.106422000 |
| C | -0.992196000 | -2.559349000 | 0.145055000 |
| C | -1.291682000 | -1.166670000 | 0.169353000 |
| C | -3.586240000 | -1.673176000 | 0.051799000 |
| C | 0.364888000 | -2.997501000 | 0.163824000 |
| C | -0.218435000 | -0.220649000 | 0.303272000 |
| C | 1.120220000 | -0.705757000 | 0.317517000 |
| C | 1.386928000 | -2.105364000 | 0.230054000 |
| C | 2.178904000 | 0.226414000 | 0.453460000 |
| C | 1.962264000 | 1.584347000 | 0.602683000 |
| C | 0.658749000 | 2.058793000 | 0.607140000 |
| C | -0.427470000 | 1.183308000 | 0.467920000 |
| H | 0.561508000 | -4.061850000 | 0.124584000 |
| H | -4.191904000 | -3.728018000 | 0.068215000 |
| H | -1.822608000 | -4.547088000 | 0.096497000 |
| H | -4.597143000 | -1.292078000 | -0.004379000 |
| H | 2.413328000 | -2.442730000 | 0.240646000 |
| H | 2.792954000 | 2.265987000 | 0.725175000 |
| H | 0.451448000 | 3.112983000 | 0.735079000 |
| N | -2.601535000 | -0.763661000 | 0.075072000 |
| O | -1.627858000 | 1.728181000 | 0.539675000 |
| H | -5.263507000 | 1.484282000 | -1.570168000 |
| H | -4.567172000 | 0.181372000 | -2.072868000 |
| H | -4.624336000 | 3.244902000 | 0.473355000 |
| H | -3.096821000 | 3.579023000 | 0.450319000 |
| O | -4.671649000 | 0.764609000 | -1.306775000 |
| O | -3.776864000 | 2.999973000 | 0.074667000 |
| Fe | -3.170351000 | 1.091865000 | -0.042405000 |
| O | 3.456215000 | -0.322223000 | 0.515412000 |
| S | 4.694104000 | 0.201824000 | -0.441732000 |
| O | 5.190818000 | 1.515738000 | 0.336613000 |

| | | | |
|---|---|---|---|
| O | 4.166798000 | 0.677180000 | -1.695336000 |
| O | 5.672179000 | -0.850599000 | -0.338153000 |
| H | 5.686419000 | 1.286685000 | 1.143339000 |

10-HBQS K(I)

ΔG = -2082.298144

| | | | |
|---|---|---|---|
| C | -2.591321000 | -3.925840000 | -0.002468000 |
| C | -1.231955000 | -4.032874000 | 0.195542000 |
| C | -0.445178000 | -2.871440000 | 0.302704000 |
| C | -1.078737000 | -1.588882000 | 0.220973000 |
| C | -3.125268000 | -2.635643000 | -0.112987000 |
| C | 0.969595000 | -2.967112000 | 0.476101000 |
| C | -0.277016000 | -0.380059000 | 0.374079000 |
| C | 1.144650000 | -0.541366000 | 0.502192000 |
| C | 1.732018000 | -1.850584000 | 0.552127000 |
| C | 1.965733000 | 0.595936000 | 0.613607000 |
| C | 1.447001000 | 1.874266000 | 0.657658000 |
| C | 0.075650000 | 2.048773000 | 0.572758000 |
| C | -0.840146000 | 0.969084000 | 0.435280000 |
| H | 1.421311000 | -3.951502000 | 0.536705000 |
| H | -3.230190000 | -4.795892000 | -0.087545000 |
| H | -0.751369000 | -5.003058000 | 0.266233000 |
| H | -4.191004000 | -2.517268000 | -0.298750000 |
| H | 2.802779000 | -1.934343000 | 0.678900000 |
| H | 2.107670000 | 2.726442000 | 0.765827000 |
| H | -0.342611000 | 3.047599000 | 0.618597000 |
| N | -2.418543000 | -1.516468000 | -0.014057000 |
| O | -2.103693000 | 1.221976000 | 0.388930000 |
| H | -4.714433000 | 3.202218000 | 0.183008000 |
| H | -5.805207000 | 3.552839000 | -0.870652000 |
| H | -3.307988000 | 3.515569000 | 2.067688000 |
| H | -2.713798000 | 2.655211000 | 0.889502000 |
| O | -5.413969000 | 2.815690000 | -0.391439000 |
| O | -3.230709000 | 3.488551000 | 1.108185000 |
| O | 3.366277000 | 0.411352000 | 0.789417000 |
| S | 4.371853000 | 0.536279000 | -0.487280000 |
| O | 4.453930000 | 2.131390000 | -0.741510000 |
| O | 3.758847000 | 0.008692000 | -1.685449000 |
| O | 5.644656000 | 0.052543000 | 0.002761000 |
| H | 4.989371000 | 2.565163000 | -0.054969000 |
| K | -4.081959000 | 0.513934000 | -1.120644000 |

10-HBQS Li(I)

ΔG = -1489.900444

| | | | |
|---|---|---|---|
| C | -3.384386000 | 3.412421000 | -0.248275000 |
| C | -2.048253000 | 3.668781000 | -0.028025000 |
| C | -1.144768000 | 2.603480000 | 0.132857000 |
| C | -1.630110000 | 1.258335000 | 0.067350000 |
| C | -3.783473000 | 2.074255000 | -0.298869000 |
| C | 0.241972000 | 2.859559000 | 0.362402000 |
| C | -0.699244000 | 0.148781000 | 0.231271000 |
| C | 0.685874000 | 0.471400000 | 0.435635000 |
| C | 1.118376000 | 1.838373000 | 0.507150000 |
| C | 1.622213000 | -0.567103000 | 0.593674000 |
| C | 1.252757000 | -1.896890000 | 0.595369000 |
| C | -0.078736000 | -2.225063000 | 0.399101000 |
| C | -1.092667000 | -1.253929000 | 0.207625000 |
| H | 0.576782000 | 3.889506000 | 0.419587000 |
| H | -4.108775000 | 4.206200000 | -0.377589000 |
| H | -1.676152000 | 4.686386000 | 0.023694000 |
| H | -4.828646000 | 1.830094000 | -0.468170000 |
| H | 2.165144000 | 2.041577000 | 0.684140000 |
| H | 1.998162000 | -2.667875000 | 0.746279000 |
| H | -0.380935000 | -3.265826000 | 0.396111000 |
| N | -2.959036000 | 1.039616000 | -0.149028000 |
| O | -2.311799000 | -1.656926000 | 0.030396000 |
| H | -4.845379000 | -3.000715000 | -0.255846000 |
| H | -6.157475000 | -2.201472000 | 0.050258000 |
| H | -3.126535000 | -4.635628000 | -0.679066000 |
| H | -2.836308000 | -3.264537000 | 0.027836000 |
| O | -5.309776000 | -2.146116000 | -0.401945000 |
| O | -3.429815000 | -4.064136000 | 0.034431000 |
| Li | -3.896089000 | -0.757246000 | -0.214690000 |
| O | 2.970279000 | -0.214543000 | 0.878760000 |
| S | 4.125181000 | -0.373358000 | -0.256627000 |
| O | 3.704569000 | 0.719597000 | -1.372792000 |
| O | 4.078431000 | -1.696061000 | -0.848464000 |
| O | 5.318819000 | 0.140597000 | 0.368318000 |
| H | 3.095739000 | 0.336757000 | -2.027169000 |

10-HBQS Mg(II)

ΔG = -1682.354643

| | | | |
|---|---|---|---|
| C | -3.279300000 | 3.057366000 | -0.357094000 |
| C | -1.955375000 | 3.393998000 | -0.176845000 |
| C | -0.993918000 | 2.392654000 | 0.037920000 |
| C | -1.394802000 | 1.019479000 | 0.073975000 |
| C | -3.608712000 | 1.706092000 | -0.313459000 |
| C | 0.376975000 | 2.752703000 | 0.219113000 |
| C | -0.389892000 | -0.012569000 | 0.297023000 |
| C | 0.975644000 | 0.413525000 | 0.450376000 |
| C | 1.321047000 | 1.804788000 | 0.414931000 |
| C | 1.980499000 | -0.547166000 | 0.662863000 |
| C | 1.703187000 | -1.895101000 | 0.768214000 |
| C | 0.393782000 | -2.321571000 | 0.622121000 |
| C | -0.675563000 | -1.433109000 | 0.379685000 |
| H | 0.641334000 | 3.803700000 | 0.196800000 |

| | | | |
|---|---|---|---|
| H | -4.046571000 | 3.800825000 | -0.526211000 |
| H | -1.636512000 | 4.430163000 | -0.198574000 |
| H | -4.640320000 | 1.399335000 | -0.447707000 |
| H | 2.355152000 | 2.086052000 | 0.553939000 |
| H | 2.500648000 | -2.602151000 | 0.958407000 |
| H | 0.150694000 | -3.374605000 | 0.694783000 |
| N | -2.723348000 | 0.723209000 | -0.106327000 |
| O | -1.863385000 | -1.947951000 | 0.249541000 |
| H | -6.181896000 | -1.420948000 | 0.722926000 |
| H | -5.495705000 | -0.387622000 | 1.655267000 |
| H | -5.033873000 | -2.665828000 | -1.964310000 |
| H | -3.718317000 | -3.420614000 | -1.623205000 |
| O | -5.408023000 | -0.850753000 | 0.812544000 |
| O | -4.307731000 | -2.714435000 | -1.330753000 |
| Mg | -3.588122000 | -1.212380000 | -0.107749000 |
| O | 3.304382000 | -0.084728000 | 0.883846000 |
| S | 4.449621000 | -0.280643000 | -0.258712000 |
| O | 3.917758000 | 0.643603000 | -1.474056000 |
| O | 4.495331000 | -1.661317000 | -0.697009000 |
| O | 5.607569000 | 0.390963000 | 0.275457000 |
| H | 3.343680000 | 0.138494000 | -2.074970000 |

10-HBQS Mn(II)

ΔG = -2633.170148

| | | | |
|---|---|---|---|
| C | -2.910098000 | 3.232453000 | -0.410398000 |
| C | -1.590747000 | 3.518271000 | -0.135394000 |
| C | -0.682216000 | 2.475709000 | 0.106769000 |
| C | -1.132133000 | 1.119031000 | 0.070074000 |
| C | -3.301698000 | 1.899040000 | -0.384447000 |
| C | 0.688345000 | 2.776658000 | 0.379818000 |
| C | -0.162788000 | 0.047610000 | 0.225957000 |
| C | 1.207585000 | 0.411783000 | 0.462708000 |
| C | 1.592716000 | 1.788460000 | 0.563684000 |
| C | 2.175398000 | -0.600000000 | 0.594135000 |
| C | 1.857549000 | -1.940334000 | 0.516851000 |
| C | 0.543192000 | -2.303392000 | 0.266745000 |
| C | -0.481390000 | -1.353000000 | 0.112032000 |
| H | 0.984560000 | 3.817830000 | 0.432823000 |
| H | -3.636711000 | 4.004914000 | -0.622653000 |
| H | -1.234796000 | 4.541904000 | -0.111605000 |
| H | -4.334035000 | 1.636999000 | -0.572873000 |
| H | 2.625810000 | 2.025133000 | 0.774071000 |
| H | 2.626334000 | -2.691614000 | 0.643060000 |
| H | 0.268285000 | -3.347934000 | 0.190080000 |
| N | -2.471006000 | 0.875893000 | -0.129181000 |
| O | -1.682751000 | -1.821942000 | -0.136026000 |
| H | -6.030338000 | -0.919826000 | 0.333218000 |
| H | -5.470953000 | 0.336455000 | 1.042164000 |
| H | -4.824936000 | -3.202509000 | -0.771908000 |
| H | -3.308025000 | -3.469536000 | -0.487644000 |
| O | -5.289706000 | -0.299965000 | 0.337828000 |
| O | -4.049836000 | -2.877120000 | -0.298379000 |
| Mn | -3.357608000 | -0.953236000 | -0.074935000 |
| O | 3.500922000 | -0.208692000 | 0.907246000 |
| S | 4.686699000 | -0.316880000 | -0.207934000 |
| O | 4.235870000 | 0.760179000 | -1.324700000 |
| O | 4.703181000 | -1.639222000 | -0.800189000 |
| O | 5.840952000 | 0.244129000 | 0.446939000 |
| H | 3.693505000 | 0.345235000 | -2.017133000 |

10-HBQS Na(I)

ΔG = -1644.661552

| | | | |
|---|---|---|---|
| C | -2.941814000 | -3.727424000 | 0.262370000 |
| C | -1.587176000 | -3.890918000 | 0.075058000 |
| C | -0.762638000 | -2.765566000 | -0.101971000 |
| C | -1.346161000 | -1.456118000 | -0.084320000 |
| C | -3.436029000 | -2.418709000 | 0.261753000 |
| C | 0.641707000 | -2.930651000 | -0.302664000 |
| C | -0.490261000 | -0.286955000 | -0.262816000 |
| C | 0.917087000 | -0.520654000 | -0.445294000 |
| C | 1.445516000 | -1.855149000 | -0.469233000 |
| C | 1.788043000 | 0.570008000 | -0.626823000 |
| C | 1.334486000 | 1.872688000 | -0.675848000 |
| C | -0.015396000 | 2.119135000 | -0.495438000 |
| C | -0.974062000 | 1.093252000 | -0.270330000 |
| H | 1.049083000 | -3.935698000 | -0.322129000 |
| H | -3.609719000 | -4.567732000 | 0.403175000 |
| H | -1.139284000 | -4.878930000 | 0.060955000 |
| H | -4.501986000 | -2.258710000 | 0.402836000 |
| H | 2.506493000 | -1.988289000 | -0.626898000 |
| H | 2.029924000 | 2.685164000 | -0.847588000 |
| H | -0.381443000 | 3.138680000 | -0.527773000 |
| N | -2.691951000 | -1.329209000 | 0.097211000 |
| O | -2.206802000 | 1.428660000 | -0.091000000 |
| H | -4.780702000 | 3.202055000 | 0.273562000 |
| H | -6.234248000 | 2.843486000 | -0.163329000 |
| H | -2.780244000 | 4.368633000 | 0.805179000 |
| H | -2.671733000 | 3.014574000 | 0.009739000 |
| O | -5.500431000 | 2.538975000 | 0.379962000 |
| O | -3.158028000 | 3.889512000 | 0.059973000 |
| Na | -4.241116000 | 0.560553000 | 0.113502000 |
| O | 3.162202000 | 0.295596000 | -0.879923000 |
| S | 4.280416000 | 0.576293000 | 0.267439000 |
| O | 3.892589000 | -0.475627000 | 1.434671000 |
| O | 4.151493000 | 1.924640000 | 0.784998000 |
| O | 5.515503000 | 0.095231000 | -0.301451000 |

| | | | |
|---|---|---|---|
| H | 3.231105000 | -0.099057000 | 2.039739000 |

10-HBQS Ni(II)

ΔG = -2990.453579

| | | | |
|---|---|---|---|
| C | -2.944437000 | 3.164874000 | -0.463920000 |
| C | -1.644760000 | 3.482332000 | -0.134095000 |
| C | -0.716576000 | 2.457156000 | 0.105808000 |
| C | -1.133218000 | 1.097245000 | 0.003180000 |
| C | -3.315062000 | 1.824020000 | -0.474262000 |
| C | 0.638719000 | 2.762285000 | 0.450073000 |
| C | -0.148416000 | 0.049190000 | 0.116801000 |
| C | 1.200445000 | 0.406134000 | 0.435666000 |
| C | 1.553126000 | 1.780602000 | 0.635152000 |
| C | 2.163635000 | -0.615064000 | 0.521911000 |
| C | 1.851807000 | -1.941857000 | 0.302756000 |
| C | 0.551412000 | -2.288572000 | -0.044118000 |
| C | -0.456707000 | -1.320844000 | -0.150061000 |
| H | 0.914820000 | 3.804319000 | 0.560794000 |
| H | -3.685517000 | 3.922768000 | -0.678102000 |
| H | -1.323202000 | 4.514946000 | -0.063655000 |
| H | -4.339428000 | 1.544742000 | -0.663393000 |
| H | 2.570771000 | 2.023794000 | 0.905946000 |
| H | 2.616722000 | -2.701767000 | 0.395253000 |
| H | 0.290369000 | -3.321331000 | -0.236816000 |
| N | -2.460455000 | 0.822882000 | -0.217233000 |
| O | -1.652000000 | -1.727606000 | -0.533945000 |
| H | -5.752088000 | -0.601611000 | 0.066519000 |
| H | -5.130553000 | -0.378732000 | 1.473523000 |
| H | -4.628131000 | -3.073361000 | -0.282339000 |
| H | -3.097559000 | -3.327692000 | -0.126834000 |
| O | -4.965577000 | -0.275434000 | 0.525744000 |
| O | -3.832423000 | -2.772025000 | 0.177573000 |
| Ni | -3.200115000 | -0.924216000 | -0.063273000 |
| O | 3.468830000 | -0.254010000 | 0.931149000 |
| S | 4.714255000 | -0.275761000 | -0.125213000 |
| O | 4.344574000 | 0.920589000 | -1.145122000 |
| O | 4.737131000 | -1.530396000 | -0.848843000 |
| O | 5.837947000 | 0.187702000 | 0.647460000 |
| H | 3.854474000 | 0.588427000 | -1.916679000 |

10-HBQS V(II)

ΔG = -2426.183701

| | | | |
|---|---|---|---|
| C | -3.131815000 | 3.156246000 | 0.174863000 |
| C | -1.810965000 | 3.515377000 | 0.028397000 |
| C | -0.833280000 | 2.520826000 | -0.151568000 |
| C | -1.229188000 | 1.154313000 | -0.196636000 |
| C | -3.457847000 | 1.803047000 | 0.136904000 |
| C | 0.546771000 | 2.871414000 | -0.285757000 |
| C | -0.216419000 | 0.140735000 | -0.384955000 |
| C | 1.150570000 | 0.536607000 | -0.485552000 |
| C | 1.502239000 | 1.922460000 | -0.440104000 |
| C | 2.130128000 | -0.468252000 | -0.644270000 |
| C | 1.819795000 | -1.807299000 | -0.732013000 |
| C | 0.483549000 | -2.201007000 | -0.646423000 |
| C | -0.502428000 | -1.244556000 | -0.479039000 |
| H | 0.812018000 | 3.921339000 | -0.257524000 |
| H | -3.914135000 | 3.887641000 | 0.323577000 |
| H | -1.510313000 | 4.555830000 | 0.055801000 |
| H | -4.491933000 | 1.502233000 | 0.269200000 |
| H | 2.540719000 | 2.203943000 | -0.542652000 |
| H | 2.600476000 | -2.542928000 | -0.871667000 |
| H | 0.202850000 | -3.243122000 | -0.719400000 |
| N | -2.556535000 | 0.828906000 | -0.049175000 |
| O | -1.786065000 | -1.665560000 | -0.427780000 |
| H | -3.549361000 | -2.094428000 | 2.348091000 |
| H | -3.183195000 | -0.576442000 | 2.501467000 |
| H | -5.753227000 | -2.318770000 | 0.445785000 |
| H | -5.531580000 | -2.383573000 | -1.106993000 |
| O | -3.475711000 | -1.253350000 | 1.874661000 |
| O | -5.119973000 | -2.142678000 | -0.264614000 |
| V | -3.372890000 | -1.073308000 | -0.132497000 |
| O | 3.460261000 | -0.047729000 | -0.809419000 |
| S | 4.543270000 | -0.199887000 | 0.417224000 |
| O | 4.924954000 | -1.761424000 | 0.358296000 |
| O | 3.873958000 | -0.021726000 | 1.682486000 |
| O | 5.662393000 | 0.607350000 | -0.002783000 |
| H | 5.532389000 | -1.948319000 | -0.379473000 |

10-HBQS V(III)

ΔG = -2425.986245

| | | | |
|---|---|---|---|
| C | -2.944267000 | 3.300964000 | -0.384542000 |
| C | -1.615193000 | 3.569904000 | -0.128825000 |
| C | -0.711531000 | 2.510693000 | 0.053024000 |
| C | -1.170611000 | 1.159529000 | -0.034815000 |
| C | -3.337343000 | 1.970462000 | -0.444351000 |
| C | 0.662518000 | 2.789271000 | 0.333578000 |
| C | -0.218933000 | 0.073410000 | 0.135865000 |
| C | 1.151914000 | 0.416711000 | 0.399237000 |
| C | 1.554846000 | 1.787904000 | 0.506676000 |
| C | 2.099039000 | -0.609872000 | 0.566851000 |
| C | 1.762009000 | -1.945885000 | 0.505340000 |
| C | 0.444802000 | -2.290139000 | 0.238812000 |
| C | -0.557230000 | -1.324952000 | 0.050163000 |
| H | 0.971133000 | 3.825658000 | 0.408361000 |
| H | -3.673605000 | 4.086281000 | -0.531571000 |
| H | -1.253672000 | 4.589555000 | -0.063118000 |

| | | | |
|---|---|---|---|
| H | -4.378026000 | 1.731235000 | -0.633202000 |
| H | 2.589349000 | 2.010137000 | 0.725822000 |
| H | 2.514990000 | -2.707286000 | 0.661778000 |
| H | 0.151560000 | -3.330927000 | 0.180387000 |
| N | -2.506068000 | 0.925889000 | -0.278927000 |
| O | -1.771368000 | -1.773617000 | -0.196582000 |
| H | -5.195604000 | -1.247913000 | 1.711079000 |
| H | -4.525217000 | 0.133793000 | 1.966329000 |
| H | -5.071332000 | -3.190187000 | -1.008488000 |
| H | -3.718572000 | -3.634677000 | -0.395605000 |
| O | -4.715412000 | -0.525019000 | 1.286240000 |
| O | -4.371468000 | -2.922161000 | -0.399515000 |
| V | -3.470921000 | -0.933419000 | -0.374016000 |
| O | 3.420762000 | -0.236950000 | 0.918877000 |
| S | 4.644292000 | -0.372869000 | -0.149954000 |
| O | 4.288178000 | 0.750066000 | -1.255952000 |
| O | 4.626252000 | -1.679440000 | -0.776040000 |
| O | 5.793927000 | 0.120552000 | 0.564845000 |
| H | 3.766890000 | 0.372202000 | -1.984710000 |

10-HBQS Zn(II)

ΔG = -3261.512847

| | | | |
|---|---|---|---|
| C | -2.834633000 | 3.307211000 | -0.288732000 |
| C | -1.490648000 | 3.566604000 | -0.132670000 |
| C | -0.580678000 | 2.512535000 | 0.049025000 |
| C | -1.046908000 | 1.159224000 | 0.059360000 |
| C | -3.239418000 | 1.978899000 | -0.250005000 |
| C | 0.804389000 | 2.805675000 | 0.236957000 |
| C | -0.089350000 | 0.069718000 | 0.232475000 |
| C | 1.291916000 | 0.437601000 | 0.422987000 |
| C | 1.699293000 | 1.812082000 | 0.427657000 |
| C | 2.260090000 | -0.562555000 | 0.620106000 |
| C | 1.940325000 | -1.903918000 | 0.656453000 |
| C | 0.624157000 | -2.276945000 | 0.448510000 |
| C | -0.409731000 | -1.345445000 | 0.223204000 |
| H | 1.116569000 | 3.843473000 | 0.233321000 |
| H | -3.563463000 | 4.093711000 | -0.429582000 |
| H | -1.115766000 | 4.583783000 | -0.142622000 |
| H | -4.289301000 | 1.730644000 | -0.352632000 |
| H | 2.743038000 | 2.041892000 | 0.585566000 |
| H | 2.707801000 | -2.645458000 | 0.836912000 |
| H | 0.344267000 | -3.323051000 | 0.457693000 |
| N | -2.397023000 | 0.949410000 | -0.086298000 |
| O | -1.596595000 | -1.858144000 | 0.007828000 |
| H | -5.541952000 | -1.758395000 | 0.942749000 |
| H | -4.924049000 | -0.878083000 | 2.066305000 |
| H | -4.820457000 | -1.450901000 | -2.147347000 |
| H | -3.756063000 | -2.569471000 | -2.028483000 |
| O | -4.984493000 | -0.986113000 | 1.107875000 |
| O | -4.300536000 | -1.960572000 | -1.511937000 |
| Zn | -3.246016000 | -0.906209000 | -0.006479000 |
| O | 3.589297000 | -0.149105000 | 0.891785000 |
| S | 4.767288000 | -0.363091000 | -0.214874000 |
| O | 4.277498000 | 0.557773000 | -1.449331000 |
| O | 4.812852000 | -1.747197000 | -0.641250000 |
| O | 5.914226000 | 0.301040000 | 0.350488000 |
| H | 3.711598000 | 0.056140000 | -2.061065000 |
